# Isomerization of pirazolopyrimidines to pyrazolopyridines by ring-opening/closing reaction in aqueous NaOH†

**DOI:** 10.1039/d4ra06345g

**Published:** 2025-01-22

**Authors:** Carlos Cifuentes, Nestor Bravo, Daniel Restrepo, Mario Macías, Jaime Portilla

**Affiliations:** a Department of Chemistry, Universidad de Los Andes Carrera 1 No. 18A-10 Bogotá 111711 Colombia jportill@uniandes.edu.co

## Abstract

An isomerization reaction of 7-aryl-3-formylpyrazolo[1,5-*a*]pyrimidines to 5-aroyl-NH-pyrazolo[3,4-*b*]pyridines proceeding with high yields in aqueous NaOH under microwave conditions is reported. This unprecedented transformation occurs by adding and eliminating a water molecule *via* an ANRORC mechanism (adding the nucleophile, ring-opening, and ring-closing) studied using DFT calculations. The product's utility was proved as they have aroyl and NH groups that simple methods and readily available reagents easily modified; likewise, their optical properties were studied, highlighting their high potential as highly emissive modular dyes (*φ*_F_ up to 99%). NMR, HRMS, and X-ray diffraction analysis resolved the products' structures.

## Abstract 2

Herein, a NaOH-promoted isomerization of pirazolopyrimidines to pyrazolopyridines, arising *via* an ANRORC mechanism studied using DFT, and the products' synthetic and photophysical utility are provided.

## Graphical abstract

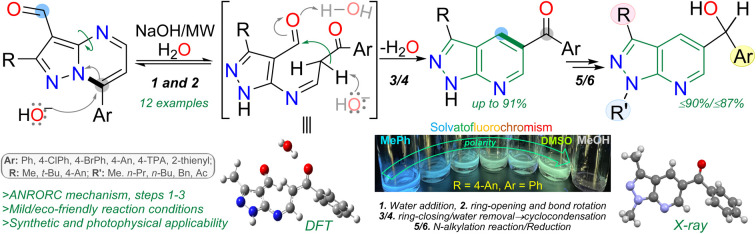

## Introduction

**Fig. 1:**
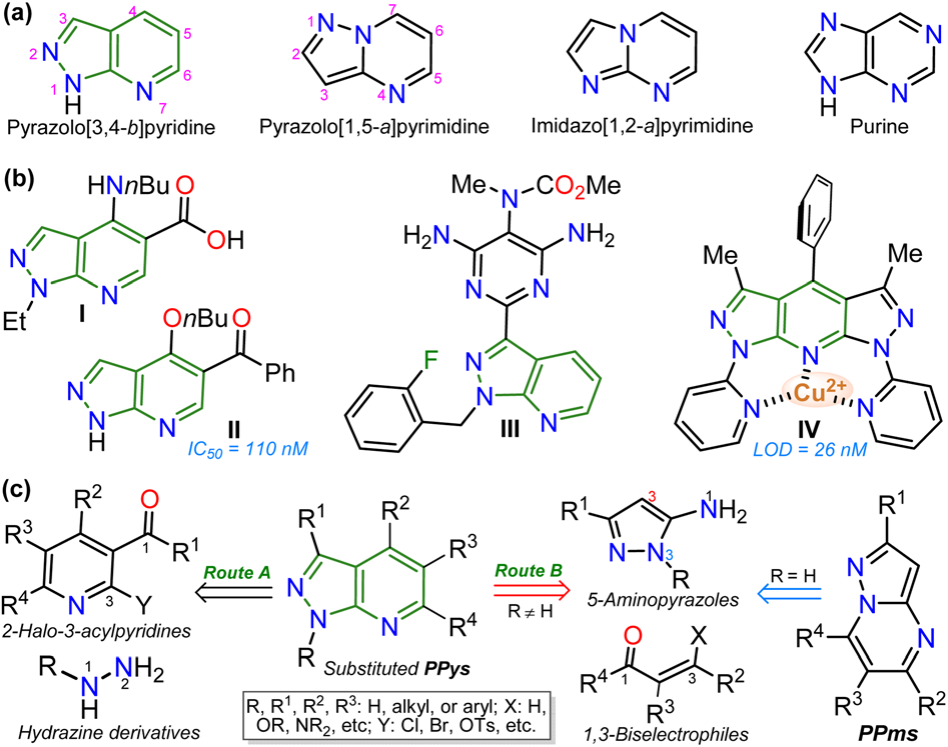
(a) Structure of aza-heteroaromatic 5 : 6 fused rings with heteroaromatic character. (b) Examples of biological and physically relevant pyrazolo[3,4-*b*]pyridines. (c) Synthesis general of pyrazolo[3,4-*b*]pyridines.

Exploring efficient methods to yield aza-heterocyclic compounds (N–HCs) is essential in organic and medicinal chemistry; this focus is inspired by the high potential to incorporate diverse functionalities into these compounds to expand their scope and pertinency.^1–3^ Along these lines, heteroaromatic rings with three or more nitrogen atoms in 5 : 6 fused rings have shown particular interest as they are structural analogs of purines found in various biological and photophysically relevant compounds (Fig. 1a).^4–7^ Especially pyrazolo[3,4-*b*]pyridines (PPys), such as the anxiolytic drug tracazolate (I), the 5-benzoyl derivative (II, inhibitor of CDK2 protein), and the antihypertensive agent riociguat (III) are N–HCs that have been explored for their therapeutic potential.^8–11^ Likewise, some PPys have been highlighted for their fluorescent properties (typical property of 5 : 6 fused ring^12,13^) in chemosensors discovery (*e.g.*, probe IV),^14–16^ (Fig. 1b).

**Scheme 1:**
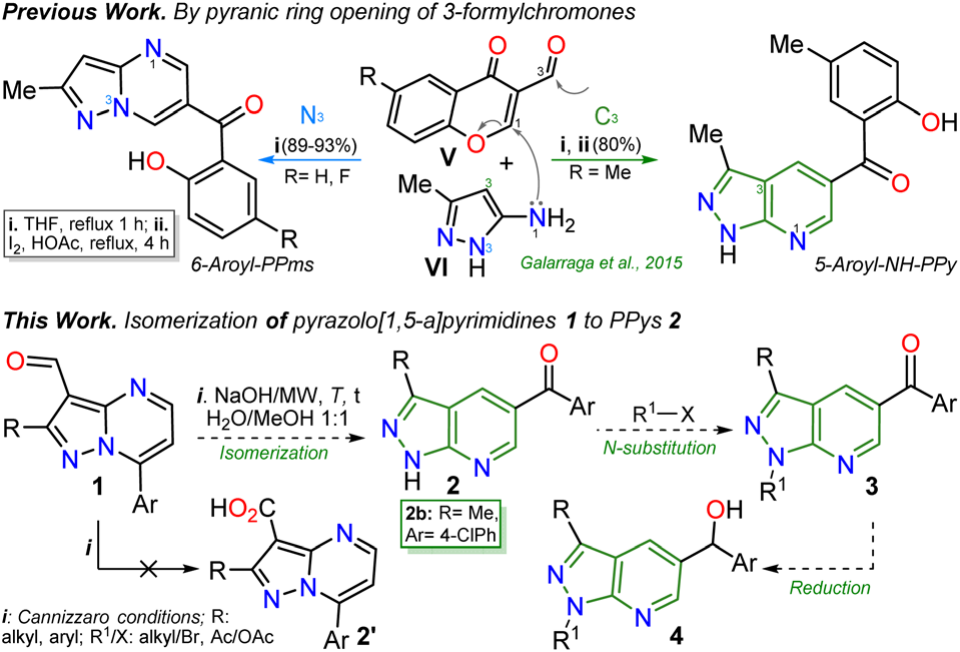
Synthesis of 5-aroyl-NH-pyrazolopyridines studied in previous work (at the top) and in this work (at the bottom).

The pyrazolo[3,4-*b*]pyridine (PPy) ring formation is achieved through cyclization reactions of 1,3-biselectrophilic pyridines (*e.g.*, 3-acyl-2-halopyridines, route A) with hydrazine derivatives and, to a greater extent, from *N*-substituted aminopyrazoles with 1,3-biselectrophilic reagents (*e.g.*, β-alkoxyenones, route B).^6,15–19^ However, by using NH-5-aminopyrazoles in route B, the reaction mainly yields pyrazolo[1,5-*a*]pyrimidines,^5,20^ and route A is limited by the poor availability of substrates^6,21^ (Fig. 1c). As a result, access to *N*-unsubstituted PPys is limited since most existing examples are route A-based,^6,20,21^ and rarer are PPys substituted with an aroyl group at position 5.^8,22–24^ Most reports on obtaining 5-aroyl-NH-pyrazolo[3,4-*b*]pyridines are found in pharmaceutical patents,^25^ and due to this, finding one article with one derivative was only possible.^20^ In that work, the authors react 3-formylchromones V with 5-amino-3-methylpyrazole (VI) to access the 5-aroyl group by a reaction proceeding with the opening of the pyranic ring in V; however, this method has limitations such as the poor availability of substrate, the recurrence of a phenolic moiety in products, and the competence with pyrazolo[1,5-*a*]pyrimidines (Scheme 1a).^20^ Importantly, 5-benzoyl-NH-pyrazolo[3,4-*b*]pyridines (similar to II in Fig. 1) were obtained in poor global yields (up to ∼5%) using synthetic strategies implying more than seven steps with a deprotection protocol for the NH-pyrazolic group.^11^

**Scheme 2:**
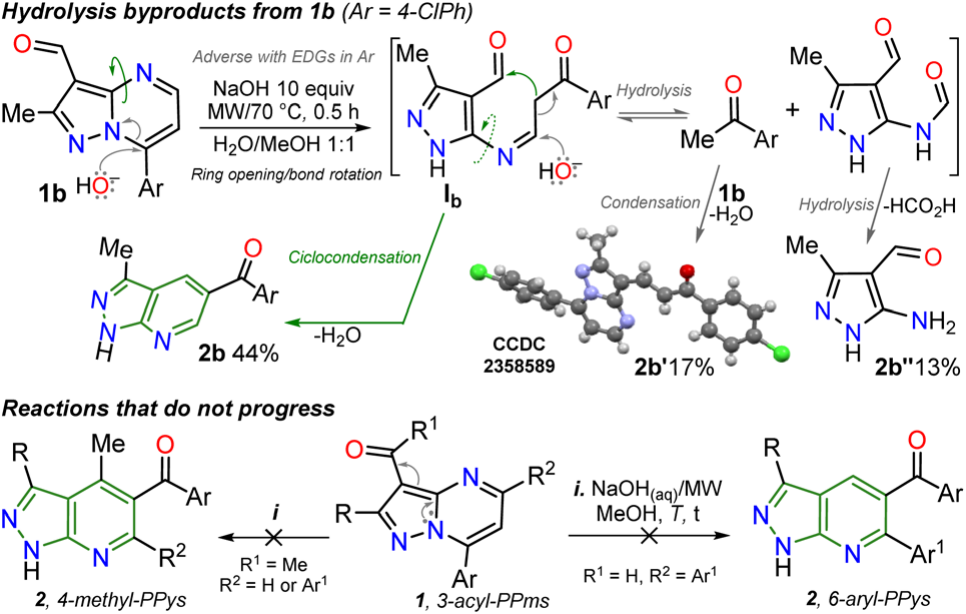
Highlighted results for the studied isomerization reaction of 1b.

Inspired by the synthetic applications of pyrazolo[1,5-*a*]pyrimidines under the microwave conditions (MWC) and our investigations in this area,^5,26–28^ the Cannizzaro reaction^26–28^ on the 3-formyl derivative 1b was utilized to access the respective carboxylic acid 2′. This acid type could be used in further studies as an N,O-donor ligand in recognition or coordination chemistry, an unusual line in these fused pyrazoles.^5^ However, an unexpected but fascinating result was found in the used reaction conditions (excess NaOH) as the isomerization product 2b was obtained instead of the desired carboxylic acid 2′. Therefore, we aimed to thoroughly study this isomerization reaction to get a novel family of pyrazolo[3,4-*b*]pyridines employing an optimized synthetic approach; we also proposed to examine the practical applicability of this protocol using functionalization strategies and the appropriate photophysical study of some representative derivatives (Scheme 2b). These investigations were proposed as isomerization products would be functionalized with easily modifiable aroyl and NH groups, and 5 : 6 fused rings have evidenced relevant fluorescent properties.^12–16^

## Results and discussion

### Synthesis

It is important to note that this is the first report on this isomerization reaction class, and due to the strange nature of the transformation, it wasn't easy to establish the structure of 2b; we achieved it by X-ray diffraction (XRD) analysis (Scheme 2, see ESI† for more details). From these exciting findings, we wonder whether the isomerization could be optimized using the 3-formylpyraolo[1,5-*a*]pyrimidine 1b (R/Ar = Me/4-ClPh, Scheme 1b) as a model substrate and a high excess NaOH (10 equiv.) at 70 °C for 30 minutes as initial conditions.

The reaction does not progress when developed in water as 1b is insoluble; thus, the reaction was carried out in water/methanol to improve the substrate solubility under microwave irradiation, obtaining 2b in 44% yield (Table 1, entry 1 *versus* 2). However, the excess NaOH used also favours the formation of enone 2b′ and the pyrazole derivative 2b′′. This result could be evidence of the ring-opening intermediate Ib that also delivered 2b, also suffers total hydrolysis – *e.g.*, Ab is converted to 4-chloroacetophenone that reacts with a second molecule of 1b – (Scheme 2a). It should be noted that the reaction (*i.e.*, NaOH with substrate 2b) only works well in H_2_O/MeOH due to its better solubility behavior; indeed, the reaction worsens in H_2_O/EtOH and does not advance in H_2_O/MeCN.

**Table 1:** Optimization for the isomerization reaction of 1b to 2b^a^

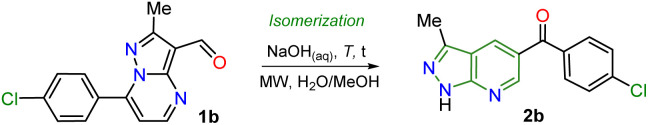					
Entry	NaOH (equiv.)	H_2_O/MeOH	*t* (min)	*T* (°C)	2b, yield (%)
1	10	1 : 0	30	70	NR
2	10	1 : 1	30	70	44
3	10	1 : 1	30	100	68
4	10	1 : 1	30	120	75
5	5	1 : 1	30	100	85
6	5	1 : 1	10	100	87
7	2	1 : 1	5	100	90
8	1	1 : 1	5	100	52
9	1.5	1 : 1	5	100	63
10	2	5 : 1	5	100	Traces
11	2	3 : 1	5	100	44
*12*	*2*	*2* : *1*	*5*	*100*	*91*
13^b^	2	2 : 1	150	Reflux	61

a Reaction conditions: 52 mg of 1b (0.19 mmol) and NaOH (7.6–76 mg) in 0.7 mL of solvent under microwave conditions; reactions run in a 10 mL sealed tube.

b Reaction under conventional heating at reflux with a heating mantle in 2 mL of solvent.

We continued the isomerization reaction study by increasing the reaction temperature, and good yields were obtained despite the slight presence of 2b′ and 2b′′. Thus, the amount of base and the reaction time were decreased (entries 3–4 *versus* 5–9), finding excellent results with 2 equivalents of NaOH at 100 °C for 5 minutes as 2b was obtained in 90% yield (Table 1, entry 7). To achieve a greener protocol, we tried to reduce the MeOH amount, noticing that 1b was wholly dissolved in a mixture with up to a 2 : 1 ratio of H_2_O/MeOH (entries 10–12). Finally, obtaining the pyrazolo[1,5-*a*]pyrimidine 2b under conventional heating at reflux was also possible but in moderate yield (Table 1, entry 13).

**Scheme 3:**
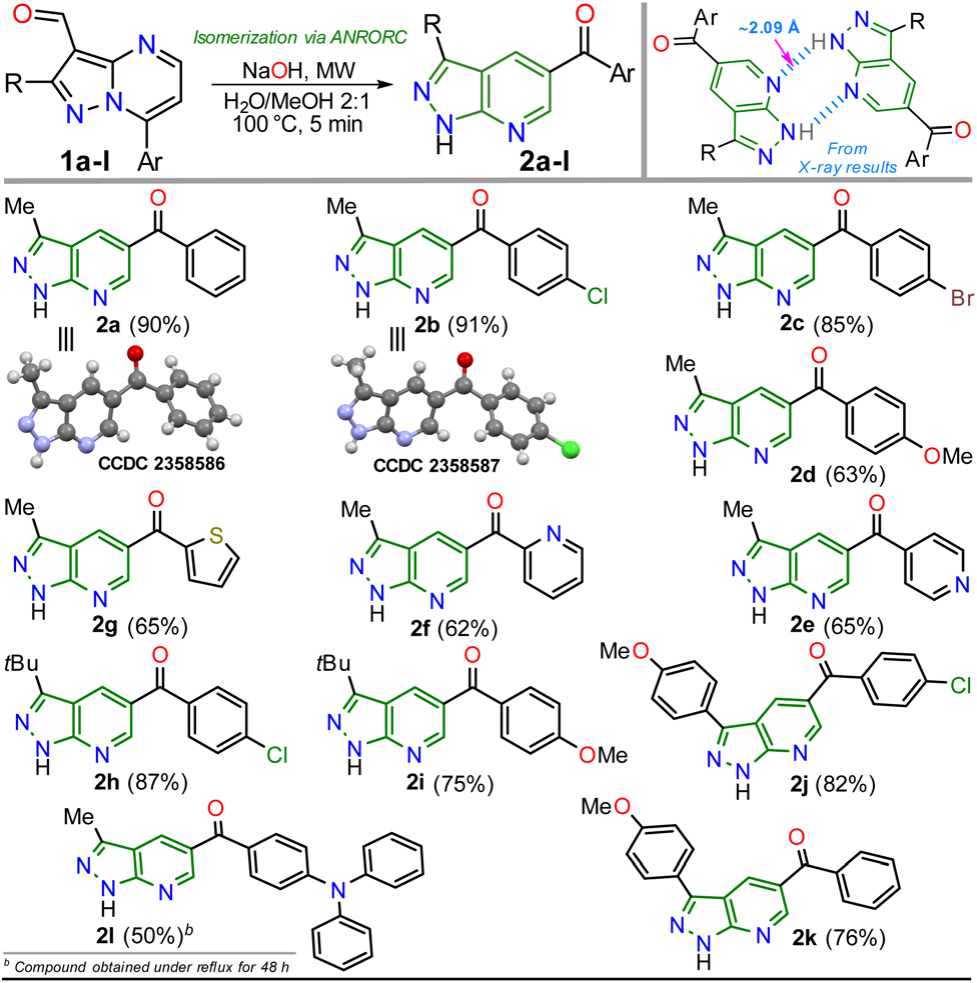
Scope of the isomerization reaction using substrates 1a–l.

With the optimal conditions established (Table 1, entry 12), we next explored the scope of the isomerization reaction using substrates 1a–l. Remarkably, excellent tolerance for substituent at positions 2 (Me, *t*Bu, and aryl) and 7 (aryl and hetaryl), with different stereoelectronic natures, was observed. However, substrates bearing electron-donating groups (EDGs) at position C7 (*i.e.*, 1d, 1g, 1i, and 1l) increase the electron density at this carbon atom^5^ (please see ^13^C NMR data of 1a–l (ref. 26–28) and LUMO orbital of 1a in ESI†) to an adverse initial nucleophilic attack decreasing reaction yields (Scheme 2a). For example, the conversion from 1l to 2l was only possible under reflux conditions for 48 hours, obtaining the lowest yield (50%) as the substrate's triphenylamine group is highly electron-donating. Curiously, although there was complete conversion, the reaction also offered moderate yields (62–65%) with highly electron-withdrawing groups (EWGs) at position C7 of substrate (as the pyridine ring in 1e and 1f); this result is due to the extraction process since 2e and 2f are slightly more soluble in aqueous medium than the other PPys (Scheme 3). Unfortunately, highly insoluble substrates (*i.e.*, 7-Ar = 4-O_2_NPh, 4-NCPh, or 4-tolyl) or containing another aryl group at position 5 (*i.e.*, 5-Ar = Ph or 4-ClPh) or an acetyl group instead of formyl are resistant to any change under the optimized reaction conditions; this result is probably as they are very polar or more stable molecules by a better π-conjugation^5,28^ (Scheme 2b).

**Scheme 4:**
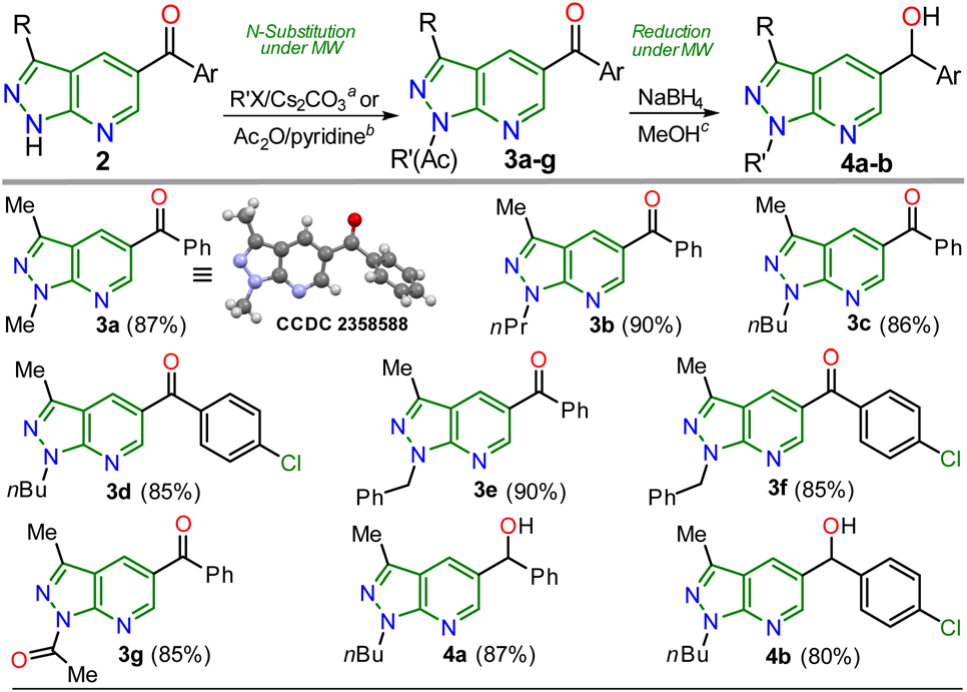
Synthesis of *N*-substituted 5-aroylpyrazolo[3,4-*b*]pyridines 3a–g and reduction products (alcohols) 4a–b.

Once the 5-aroyl-NH-pyrazolo[3,4-*b*]pyridines 2a–l were obtained, we developed functionalization reactions to access new derivatives with this relevant heterocyclic core, obtaining strategic compounds with a high potential in medicinal and organic chemistry.^20–24^ In this way, the *N*-alkylation reaction on the pyrazole moiety was conducted using alkyl halides (2 equiv.) and caesium carbonate (2 equiv.) in MeCN (2 mL) at 80 °C for 15 minutes under MWC, obtaining 3a–f in high yields (85–90%); the amide ion was previously generated at 50 °C for 3 minutes also under microwave conditions (Scheme 4).

As expected, the acetylation reaction of 2a (0.25 mmol) with acetic anhydride (2 equiv.) in pyridine (2 mL) at 120 °C for 30 minutes in MWC efficiently afforded the *N*-acetyl derivate 3g in 85% yield. Ultimately, the reduction reaction of the carbonyl group in 3c–d was successfully developed using NaBH_4_ (2 equiv.) in methanol at 100 °C for 5 minutes under MWC, forming alcohols 4a–b in high yields (80–87%, Scheme 4). Pleasantly, all the obtained pyrazolo[3,4-*b*]pyridines are structurally novel and functional, so they are potentially helpful for new synthetic procedures, allowing their application in various fields of chemistry; for example, compounds 2a–l could be used as *N*,*N*-donor ligands to access coordination complexes, as observed in the hydrogen bonding interactions found by XRD studies (Scheme 3 and Fig. S48†). Likewise, structures of enone 2b′ (Scheme 2), 2a–b (Scheme 3), and *N*-methyl derivative 3a (Scheme 4) were solved using single-crystal XRD analysis.^29^ All the obtained compounds (2b′, 2b′′, 2a–l, 3a–g, and 4a–b) were characterized through NMR and high-resolution mass spectra (HRMS) analysis (see ESI† for details).

### Photophysical studies

**Fig. 2:**
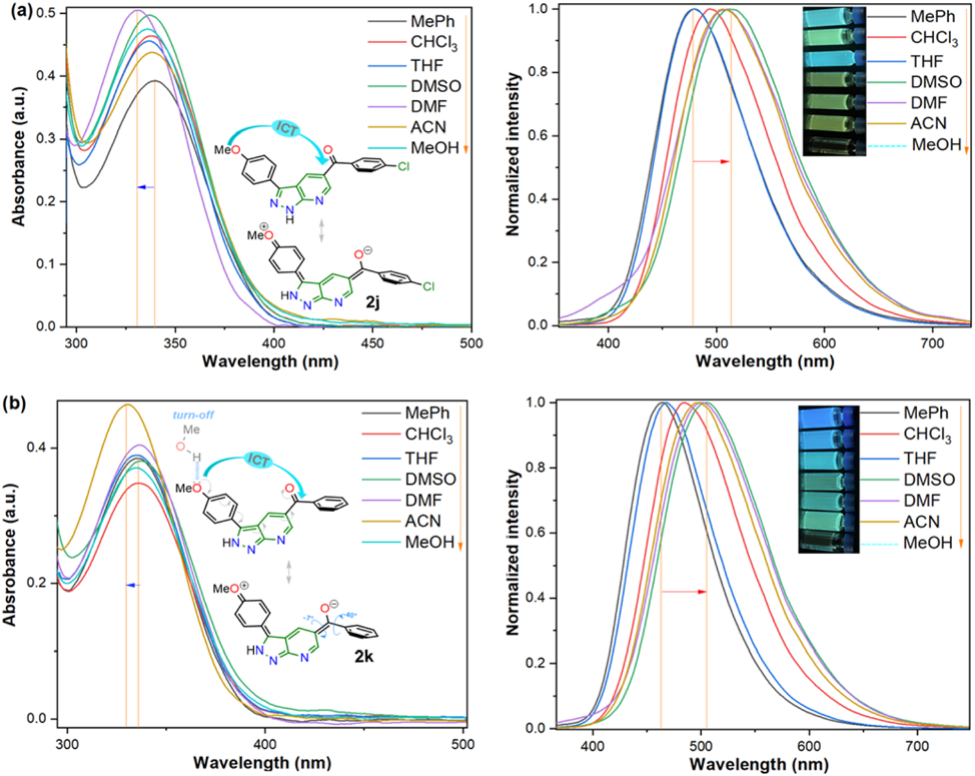
Absorption (left) and emission (right, *λ*_ex_ = 335 nm) spectra and photographs of probes (a) 2j and (b) 2k in different solvents (50 μM) at 20 °C.

During synthetic experiments, we noticed that some products exhibit luminescent properties in solution and solid state under exposure to UV light, mainly the two derivatives with 4-anisyl group (4-MeOPh) at position 3; thus, the photophysical study of 2j–k was conducted to establish the scope of their donor–π–acceptor molecular architecture (D–π–A or MeO–π–C=O) in organic fluorophores development. Due to their synthetic potential, these results would allow us to document PPys 2j–k as strategic intermediates of new functional fluorophores, considering some fluorescent dyes bearing pyrazolo[1,5-*a*]pyridine ring.^15,16,30^ In addition to the initially observed luminescence, probes 2j–k were photophysically studied since the 4-anisyl group, bonded to diverse chromophores, has proven helpful in this area, mainly by intramolecular charge transfer (ICT) phenomena using 5 : 6 fused aza-heterocyclic rings.^31–34^ In this manner, the absorption and emission spectra of fluorophores 2j–k were recorded in solvents of different polarity (solvent, Δ*f*): toluene (MePh, 0.013), chloroform (CHCl_3_, 0.149), tetrahydrofuran (THF, 0.210), dimethylsulfoxide (DMSO, 0.265), *N*,*N*-dimethylformamide (DMF, 0.275), acetonitrile (ACN, 0.306), and methanol (MeOH, 0.309) (Fig. 2 and Table 2).

**Table 2:** Photophysical data of 3-(4-anisyl)pyrazolo[3,4-*b*]pyridines 2j–k^a^

Probe	Solvent^b^	*λ*_abs_ (nm)	*ε* (M^−1^ cm^−1^)	*λ*_em_ (nm)	SS (cm^−1^)	*ϕ*_F_	*B* (*ε* × *ϕ*_F_)
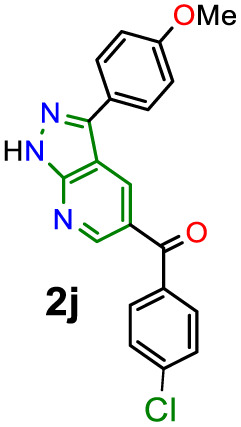	MePh	339	7840	478	8578	0.99	7762
	CHCl_3_	337	9260	493	9389	0.78	7223
	THF	336	9100	479	8885	0.78	7098
	DMSO	338	8740	512	10 054	0.53	4632
	DMF	337	9940	508	9988	0.31	3082
	ACN	331	10 100	507	10 487	0.30	3030
	MeOH	335	9500	519	10 582	0.0069	66
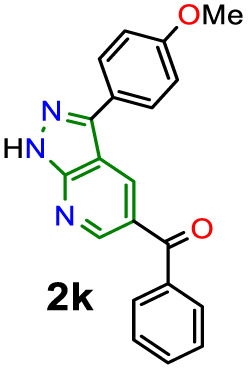	MePh	335	7700	464	8299	0.93	7161
	CHCl_3_	335	6940	484	9189	0.88	6107
	THF	334	7760	468	8572	0.84	6519
	DMSO	337	7560	503	9792	0.96	7258
	DMF	336	8080	499	9721	0.77	6222
	ACN	330	9280	498	10 222	0.74	6867
	MeOH	334	7400	524	10 856	0.01	74

a Quantum yield (*ϕ*_F_) values were determined using Prodan as a standard. Absorbance (ab), fluorescence emission (em), molar absorption coefficient (*ε*), Stokes shift (SS), and calculated bright (*B*) data are also shown.

b Variation of orientation polarizability (Δ*f*): 0.013, 0.149, 0.210, 0.265, 0.275, 0.306, and 0.309, respectively.

Compounds 2j–k displayed absorption spectra with the typical band of S_0_ → ICT transitions (*i.e.*, MeO → C=O) at around 335 nm with extinction coefficient (*ε*) values of 7840–10 100 M^−1^ cm^−1^ for 2j (4-ClBz) and 6940–9280 M^−1^ cm^−1^ for 2k (Bz). These results revealed a slight blue-shifted and best absorptivity by increasing solvent polarity, with 2j as the best chromophore due to its higher number of electrons (Fig. 2, left). Both probes exhibited high fluorescence quantum yield (*ϕ*_F_) and brightness (*B* = *ε* × *ϕ*_F_) values, mainly in less polar solvents (*e.g.*, *ϕ*_F_ = 99% and *B* = 7762 for 2j in MePh), with a sharp decrease in the emission intensity of 2j in DMF or ACN (Fig. 2, right). This is owing to the stability of non-emitting excited states, meaning that the energy received by solute is dissipated in non-radiative ways rather than released as light. Polar solvents can help with nonradiative relaxing by creating an environment where vibrational energy can be more efficiently transmitted to the solvent; this reduces the quantity of energy emitted as light, decreasing the fluorescence quantum yield.^35–40^ Moreover, both probes displayed significant Stokes shifts (8578–10 582 cm^−1^ for 2j and 8299–10 856 cm^−1^ for 2k) with a marked red-shifted by increasing solvent polarity (Fig. 2 and S49,†Table 2).

**Fig. 3:**
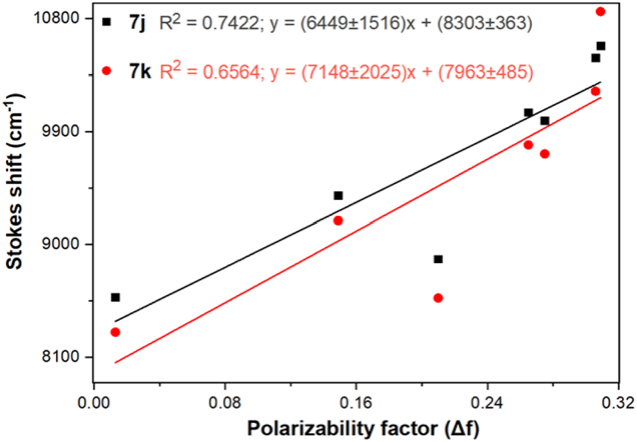
Lippert–Mataga plots (Δ*ν vs.* Δ*f*) for probes 7j–k in different solvents.

Importantly, the photophysical properties 2j–k were dramatically affected in methanol due to strong hydrogen bonding interactions of their anisyl group with solvent molecules (*ϕ*_F_ ≤ 1%). This solvent acts as a fluorescence quencher in 2j–k, blocking the ICT process to afford energy transfer without light emission; however, this property could be relevant in determining water or ethanol in some organic solvents or distilled spirits (Fig. 2).^36,37^ In addition, the Lippert–Mataga plots for 2j–k illustrate their typical behaviour in the excited states with the variation of orientation polarizability (Δ*f*) of the solvent; as expected, they evidenced different interactions with the evaluated solvents, leading to two unique straight-line correlations with positive slopes (Fig. 3). This can be explained as a general solvation effect in low polarity solvents; in contrast, specific interactions like dipole–dipole in polar solvents stabilise the system; it would not be dependable to compute the change in the dipole moment using these numbers due to the low *R*^2^ value, suggesting no strong association polarity factor/Stokes shift. However, from the positive slopes in the graphs, it is possible to see the defined trends according to the slope direction for each probe, demonstrating positive solvatofluorochromism.^35–40^

Accordingly, it is proved that the ICT phenomenon in fluorescent probes 2j–k favours a good charge separation within these molecules through resonance from the anisyl group towards the carbonyl group (*i.e.*, MeO → C=O). It should be noted that the benzene ring in the aroyl group at position 5 of the aza-heterocyclic core does not greatly affect the ICT process as this ring is not coplanar with the D–π–A system of fluorophores 2j–k (Fig. 2); this structural characteristic could be determined through the dihedral angles found from crystallographic X-ray diffraction analyses for similar structures (*i.e.*, 2a and 2b, Fig. S48 in ESI†). Finally, from the significant photophysical properties found for probes 2j–k and the good synthetic results involving compounds 2a–l, 3a–g, and 4a–b, we can establish that the heterocyclic core of pyrazolo[3,4-*b*]pyridine is a promisor functional fluorophore; specifically for developing applications in coordination chemistry, diagnostic bioimaging in living systems, chemosensors discovery, photosensitizers, or organic materials science.^13,31–40^

### DFT calculations

**Scheme 5:**
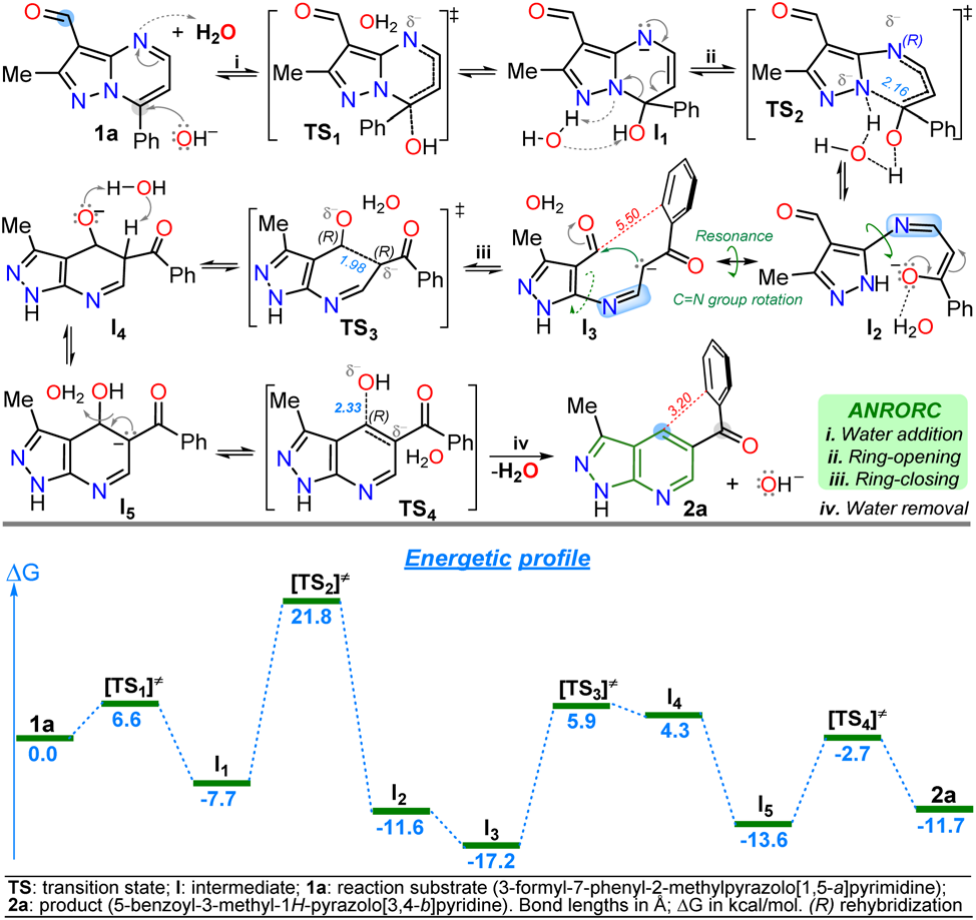
Proposed mechanism (at the top) for the isomerization reaction of 1a to 2a with its respective energetic profile (at the bottom).

The primary reaction was studied using density functional theory (DFT) B3LYP/6-311+(d,p) calculations to understand better the mechanism of the discovered reaction with the associated relative free energies (Δ*G*) by substrate 1a.^41,42^ The relative free energy calculated for the overall reaction is −11.79 kcal mol^−1^, indicating greater product 2a stability than substrate 1a due to the high aromatic character of the pyridine ring in 2a.^5,6^ It is important to note that a negative charge is induced within structures during the reaction due to the medium nature and the initial attack of the hydroxyl anion; however, the isomerization involves the addition and elimination of a water molecule through an ANRORC (adding the nucleophile, ring-opening, and ring-closing) mechanism. Implicit water was initially modelled, and explicit modelling of a single water molecule proved crucial in stabilizing transitional states by clustering negative charges within acceptor hydrogen groups along structures. Incorporating a hydroxyl group at position 7 on substrate 1a exhibits lower energy than the initial structure, implying greater stabilization for intermediate I_1_, allowing the pyrimidine ring-opening to find metastable state cause of possible intermediates I_2_ and I_3_. This study supports that rotation in the imine group of I_2_ is favourable, leading to the pyridine ring-closing to afford I_4_ by reacting the stabilized carbanion with the C-carbonyl in I_3_ (Scheme 5).

The explored mechanism reveals four crucial transition states involving the bonding of the hydroxyl group TS_1_, its detachment TS_4_, and the isomerization process *via* ring-opening TS_2_ and ring-zar^13^ closing TS_3_. The free energies in TS_2_/TS_3_ are higher than in TS_1_/TS_4_, indicating that the most critical reaction steps involve opening the pyrimidine ring and closing the pyridine ring under MWC. The energy levels of structures TS_3_ and I_4_ are closely matched. Deprotonation of the acidic hydrogen at position 5 of the dihydro-pyridine ring in I_4_ reduces the relative free energy, resulting in the discovery of a metastable state I_5_ that finally facilitates the formation of a hydroxyl group (base regeneration) to yield the heteroaromatic product 2a (Scheme 5). Notably, the anionic intermediate I_3_ has the lowest energy in the energetic profile, even lower than 2a, due to the implicit water solvation model used in the presence of the hydroxyl anion; indeed, 2a has an electronic repulsion between the two rings of the molecule at 3.20 Å in different planes. In contrast, I_3_ has larger distances (5.50 Å) between the ring of the aroyl group and the nearest carbon atom that would be part of the fused ring, reducing intramolecular electronic repulsions (Scheme 5 and Fig. S51†).

## Conclusions

In summary, a NaOH-mediated isomerization reaction of 7-aryl-3-formylpyrazolo[1,5-*a*]pyrimidines to 5-aroylpyrazolo[3,4-*b*]pyridines was successfully developed under microwave conditions at 100 °C. Eleven isomerization products were obtained in high yields (up to 91%) employing mild reaction conditions in an aqueous medium and cheap reagents. Notably, a mechanistic route for this transformation was also proposed based on DFT calculations and the experimental results; this implies an ANRORC route that started with the hydroxy anion addition at the C7 of the aza-heterocyclic core of the substrate. Additionally, the obtained ketoamines have strategic functional groups (aroyl and NH-heteroaryl) that were easily modified by simple approaches and readily available reagents. The structure of all the obtained compounds was established based on NMR, HRMS, and X-ray diffraction studies. Likewise, the two more luminescent obtained ketoamines were photophysically studied, indicating the utility of the 4-anisyl group and 5 : 6 fused rings in the optoelectronic properties of fluorophores acting through ICT phenomena. Fluorescence quantum yields of up to 99% were obtained with dyes having positive (redshift) solvatofluorochromism, highlighting the high potential of these two compounds as functional fluorophores; for example, as methanol acted as a solvent fluorescence quenching blocking the ICT process in the two tested ketoamines, new applications in sensing water or ethanol in different mediums could be possible.

## Data availability

The experimental data, NMR and HRSM spectra, crystallographic and photophysical details (CCDC: 2a2358586, 2b2358587, 2b′2358589, and 3a2358588), and computational details have been included in the ESI.

## Author contributions

The individuals listed as authors have contributed to developing this manuscript, and no other person was involved. The authors' contributions included: C. C. and N. B. carried out experiments and literature review; D. R. and M. M. carried out computational and XRD studies, respectively; and J. P. the composition of the original draft, supervision, and sources. All authors have read and agreed to the published version of this manuscript.

## Conflicts of interest

The authors declare no competing financial interest.

## Supplementary Material

RA-015-D4RA06345G-s001

RA-015-D4RA06345G-s002
