# Supplementary material for: Isomerization of pirazolopyrimidines to pyrazolopyridines by ring-opening/closing reaction in aqueous NaOH
Source: RSC Adv. 2025 Jan 22;15(3):2078–85. doi: 10.1039/d4ra06345g (PMC11751703; doi:10.1039/d4ra06345g)
Supplement: RA-015-D4RA06345G-s001 [file RA-015-D4RA06345G-s001.pdf]

Supplementary Information (SI) for RSC Advances.
This journal is © The Royal Society of Chemistry 2025

Electronic supplementary information (ESI)

Isomerization of pirazolopyrimidines to pyrazolopyridines by ring-opening/closing reaction in aqueous NaOH

Carlos Cifuentes, Nestor Bravo, Daniel Restrepo, Mario Macías, and Jaime Portilla*

Department of Chemistry, Universidad de Los Andes, Carrera 1 No. 18A-10, Bogotá 111711, Colombia

*E-mail: jportill@uniandes.edu.co; Tel: +57 1 3394949. Ext. 2080

Content

1. Overview of substrates and products numbering 2
2. Experimental procedures and characterization data 2
2.1. General Information 3
2.2. General Procedures 3
3. Data Characterization 5
4. HRMS analysis 11
5. Copies of NMR spectra 33
6. Crystallographic and photophysical details 33
7. Computational studies 55
8. References 60

1. Overview of substrates and products numbering

(a) 7-Aryl-3-formylpyrazolo[1,5-*a*]pyrimidines

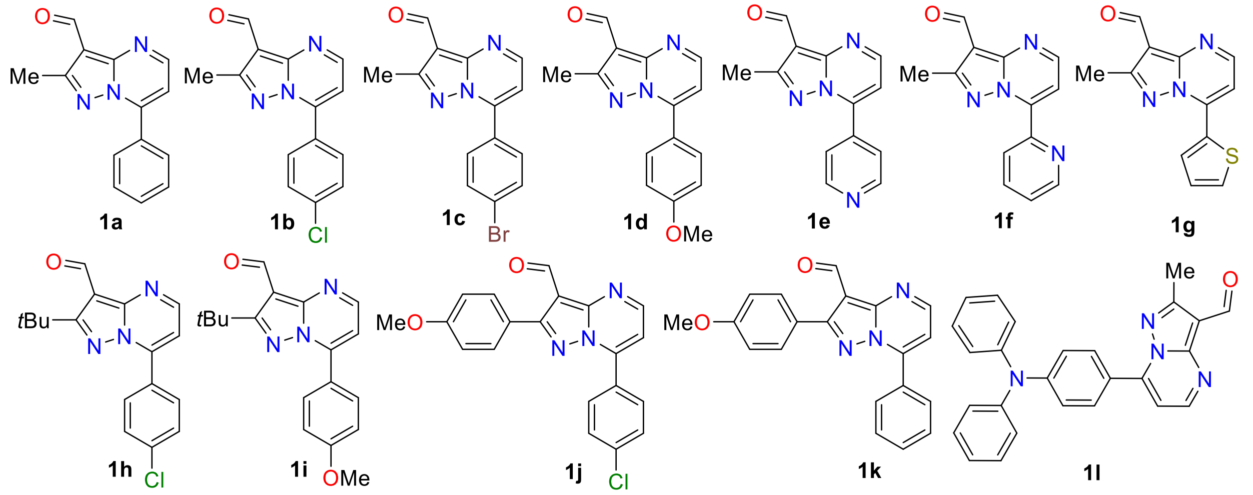

(b) 5-aroylpirazolo[3,4-*b*]pyridines

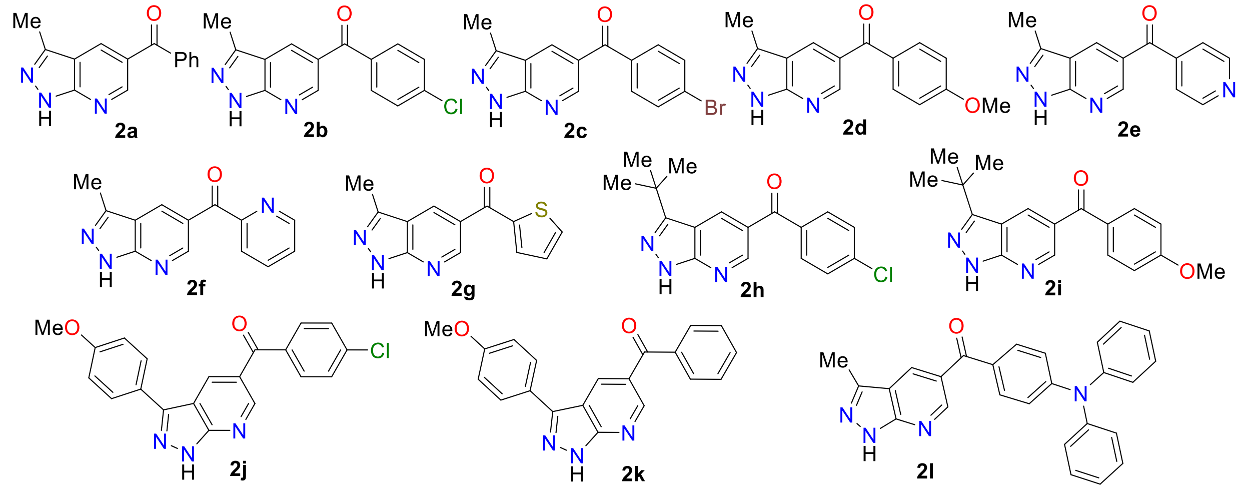

(c) Functionalized 3-methylpirazolo[3,4-*b*]pyridines

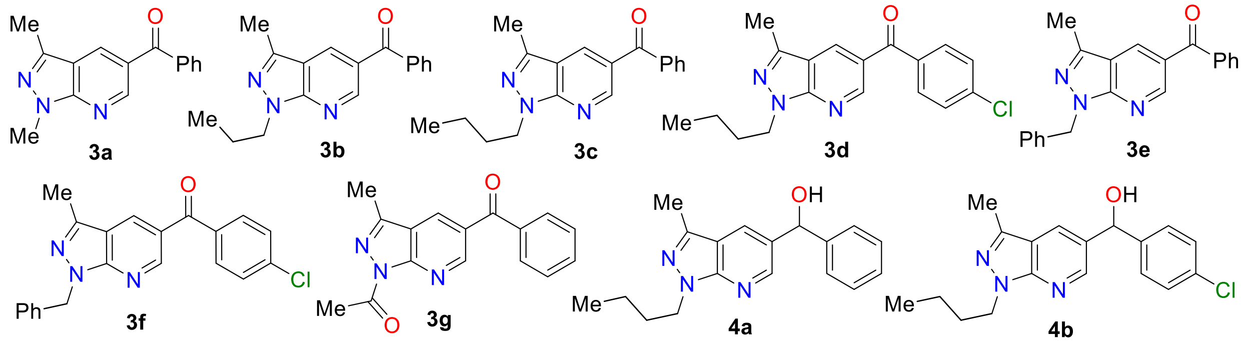

Fig. S1 Structures of a) substrates **1a-l**, b) products **1a-l**, and c) functionalized compounds **3a-g** and **4a-b**

2. Experimental procedures and characterization data

2.1. General Information

All reagents were purchased from commercial sources and used without further purification unless otherwise noted. All starting materials were weighed and handled under air at room temperature. The reactions were monitored by TLC and visualized by a UV lamp (at 254 or 365 nm). Flash chromatography was performed on silica gel (230−400 mesh) and DCM or DCM/MeOH (60:1 v/v) as eluents. All reactions under microwave (MW) irradiation were performed in a sealed reaction vessel (10 mL, max pressure = 300 psi) having a Teflon-coated stir bar (from CEM). MW-assisted reactions were performed in a CEM Discover SP-focused ME (*v* = 2.45 GHz) reactor equipped with a built-in pressure measurement sensor and a vertically focused IR temperature sensor. Controlled temperature, power, and time settings were used for all reactions.

NMR spectra for this work were recorded at 400 MHz (^{1}H) and 101 MHz (^{13}C) at 298 K, and data were recorded in $CDCl_3$ using as internal standards the residual nondeuterated signal for ^{1}H NMR (7.26 ppm) and the deuterated solvent signal for ^{13}C NMR (77.16 ppm) spectroscopy. DEPT spectra were used to assign carbon signals with the help of 2D experiments. Chemical shifts (δ) are given in parts per million (ppm) and coupling constants (*J*) in Hertz. The following abbreviations are used for multiplicities: s = singlet, d = doublet, t = triplet, q = quartet, dd = doublet of doublets, and m = multiplet. Melting points were determined by a capillary melting point apparatus and were uncorrected. A Q-TOF spectrometer recorded high-resolution mass spectra (HRMS) via electrospray ionization (ESI). The X-ray intensity data were measured at 25(2) °C using CuKα radiation (λ = 1.54184 Å) by ω scans in an Agilent SuperNova, Dual, Cu at Zero, Atlas four-circle diffractometer equipped with a CCD plate detector. Crystallographic data for the structural analysis have been deposited in the Cambridge Crystallographic Data Center (CCDC), with deposition numbers 2358586 for **2a**, 2358587 for **2b**, 2358589 for **2b'** (chalcone), and 2358588 for **3a** (*N*-methyl derivative); copy of this data may be obtained free from CCDC, 12 Union Road, Cambridge, CB2 1EZ, UK (Fax: +44-1223-336033; e-mail: deposit@ccdc.cam.ac.uk). The absorption spectra were measured on a Varian Cary 100 spectrophotometer, while those for emission were recorded using a Cary Eclipse S19 fluorescence spectrophotometer (the two Agilent Technologies devices). UV-vis and fluorescence measurements were performed at room temperature (~20 °C) in quartz cuvettes having a path length of 1 cm. For fluorescence studies, both the excitation and emission slit widths were 5 nm.

2.2. General Procedures

*2.2.1. General Procedure for the Synthesis of Pyrazolo[1,5-a]pyrimidine-3-carbaldehydes **1a–j**.*

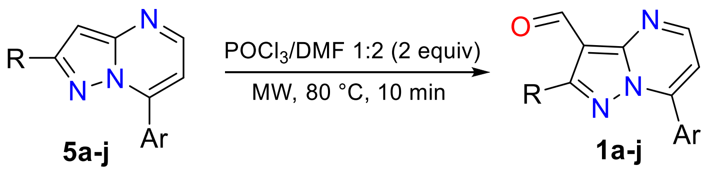

Heteroaldehydes **1a-j** were synthesized in high yields using three MW-assisted reaction steps (*i.e.*, *i.* *β*-anaminones formation, *ii.* their cyclocondensation with aminopyrazoles, and *iii.* formylation reaction) starting from aryl methyl ketones according to protocols previously reported in our investigating group;[1–4] the last step in preparing **5a-j** is described below. A mixture of phosphoryl chloride (2.0 mmol, 187 µL) and 310 µL of anhydrous *N,N*-dimethylformamide (4 mmol) was added into a sealed tube containing a Teflon-coated magnetic stirring bar and cooled to 0 °C and stirring for 30 min. Later the formylating agent solution was added dropwise to a solution of **4** (1 mmol) in 300 µL of DMF under stirring at 0 °C for 10 min; then, the reaction was warmed to room temperature and stirred for 15 min, and finally, the mixture was irradiated with microwaves at 80 °C (100W) for 10 min in a sealed tube under stirring (Teflon-coated magnetic bar). The resulting reaction mixture was cooled to 50 °C by airflow; after, the mixture was maintained at pH = 7 by adding an aqueous solution of $NaHCO_3$ (20%), and the reaction mixture was vigorously stirred at room temperature for 30 min. The precipitate was filtered, washed with cold water (2 × 5 mL), and purified by flash chromatography on silica gel (eluent DCM) to afford the pure products **1a-l**.

2.2.2. General procedure for the synthesis of 5-aroyl-NH-pyrazolo[3,4-b]pyridines **2a-l**

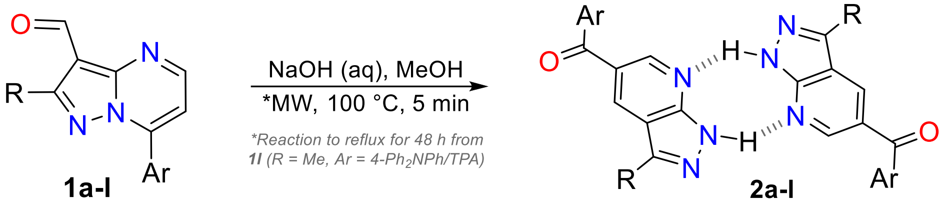

A mixture of **5a-j** (0.5 mmol), NaOH 1M (1 mL, 1 mmol), and 0.5 mL of MeOH was added into a sealed tube containing a Teflon-coated magnetic stirring bar and was irradiated with MW at 100 °C (130W) for 5 min; the reaction from substrate **1l** (R = Me, Ar = TPA) was carried out under reflux for 48 h. The resulting reaction mixture was cooled to 50 °C by airflow, diluted with DCM, and hydrolyzed. The organic layer was separated, and the aqueous layer was extracted thrice with DCM. The combined organic layers were washed with brine, dried over anhydrous Na_2SO_4, filtered, and concentrated under vacuum to give the crude product. Flash chromatography of this material silica gel (eluent DCM/MeOH 60:1 *v/v*) afforded the pure products **2a-l**.

2.2.3. General procedure for the synthesis of N-substituted pyrazolo[3,4-b]pyridines **3a-f**

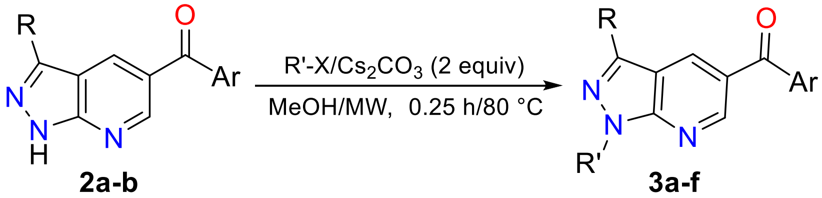

A mixture of caesium carbonate (2 equiv) and **6a-l** (1 equiv) in anhydrous MeCN (2 mL) in a sealed tube and stirred (Teflon-coated magnetic bar) was irradiated at 50 °C for 3 min. To the generated suspension, the respective alkyl halide (R'-X, 2 equiv) was added in one portion, and the resulting mixture was irradiated under MW 80 °C (100W) for 15 min. The resulting reaction mixture was

cooled to 50 °C by airflow, diluted with DCM, and hydrolyzed. The organic layer was separated, and the aqueous layer was extracted thrice with DCM. The combined organic layers were washed with brine, dried over anhydrous sodium sulfate, filtered, and concentrated under vacuum. Flash chromatography of this material silica gel (eluent DCM) yielded the desired products **3a-f**.

*2.2.4. General synthesis of 1-acetyl-5-benzoyl-3-methylpyrazolo[3,4-b]pyridine (**3g**)*

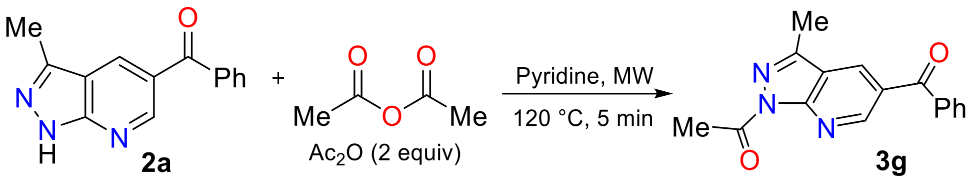

A mixture of caesium **2a** (1 equiv), acetic anhydride (Ac_2O, 2 equiv), and pyridine (2 mL) in a sealed tube was irradiated with MW under stirring (Teflon-coated magnetic bar) at 120 °C (140W) for 5 min. The resulting reaction mixture was cooled to 50 °C by airflow, HCl 5% (5 mL) was added, and the product was extracted with DCM (3 × 7 mL). The organic layer was separated, and the aqueous layer was extracted thrice with DCM. The combined organic layers were washed with brine, dried over anhydrous sodium sulfate, filtered, and concentrated under vacuum to give the crude product. Flash chromatography on silica gel (eluent DCM) afforded the desired product **3g**.

*2.2.5. General synthesis of 5-(aryl)hydroxymethyl-1-butylpyrazolo[3,4-b]pyridines **4a-b***

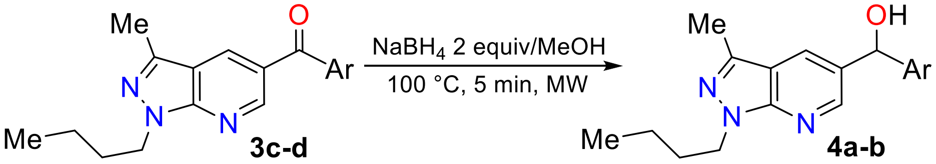

A mixture of **3c-d** (1 equiv), $NaBH_4$ (2 equiv), and methanol (2 mL) in a sealed tube was irradiated with MW under stirring (Teflon-coated magnetic bar) at 100 °C (120W) for 5 min. The resulting reaction mixture was cooled to 50 °C by airflow, HCl 5% (5 mL) was added, and the product was extracted with DCM (3 × 7 mL). The organic layer was separated, and the aqueous layer was extracted thrice with DCM. The combined organic layers were washed with brine, dried over anhydrous Na_2SO_4, filtered, and concentrated under vacuum to give the crude product, which was chromatographed (silica gel, eluent DCM) to afford alcohols **4a-b**.

3. Data Characterization

*(3-Methyl-1H-pyrazolo[3,4-b]pyridin-5-yl)(phenyl)methanone (**2a**).* By the general procedure

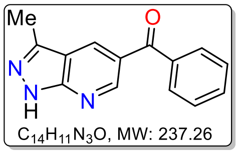

from aldehyde **1a** (118 mg, 0.5 mmol), **2a** was obtained as a yellow solid (106 mg, 90%). Mp 150–151 °C. ^1H NMR (400 MHz, $CDCl_3$): δ = 2.66 (s, 3H), 7.52–7.83 (m, 5H), 8.59 (s, 1H), 9.07 (s, 1H), 12.91 (s-br, 1H) ppm. $^{13}C\{^1H\}$ NMR (100 MHz, $CDCl_3$): δ = 12.7 (CH_3), 114.6 (C), 126.6 (C), 128.8 (CH), 130.0 (CH), 132.9 (CH), 133.0 (CH), 137.6 (C), 144.7 (C), 151.2 (CH), 153.5 (C), 194.9 (C) ppm. HRMS (ESI) m/z: $[M + H]^+$ calcd. for $C_{14}H_{12}N_3O^+$ 238.0975; found 238.0979.

*(4-Chlorophenyl)(3-methyl-1H-pyrazolo[3,4-b]pyridin-5-yl)methanone (**2b**).* By the general route

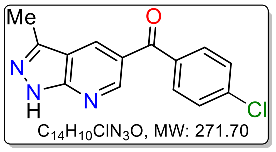

from aldehyde **1b** (136 mg, 0.5 mmol), **2b** was obtained as a yellow solid (126 mg, 91%). Mp 203–204 °C. ^{1}H NMR (400 MHz, $CDCl_3$): δ = 2.66 (s, 3H), 7.52 (d, *J* = 8.0 Hz, 2H), 7.78 (d, *J* = 8.0 Hz, 2H), 8.55 (d, *J* = 2.0 Hz, 1H) ppm, 9.04 (d, *J* = 2.0 Hz, 1H) ppm, 12.72 (s-br, 1H). $^{13}C\{^1H\}$ NMR (100 MHz, $CDCl_3$): δ = 12.8 (CH_3), 114.6 (C), 126.3 (C), 129.1 (CH), 131.4 (CH), 132.9 (CH), 135.9 (CH), 139.4 (C), 144.8 (C), 151.0 (CH), 153.5 (C), 193.7 (C) ppm. HRMS (ESI) m/z: $[M + H]^+$ calcd. for $C_{14}H_{11}{}^{35}ClN_3O^+$ 272.0585; found 272.0598.

*(4-Bromophenyl)(3-methyl-1H-pyrazolo[3,4-b]pyridin-5-yl)methanone (**2c**).* By the general route

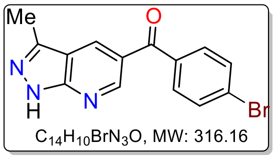

from **1c** (158 mg, 0.5 mmol), **2c** was obtained as a yellow solid (134 mg, 85%). Mp 206–207 °C. ^{1}H NMR (400 MHz, $CDCl_3$): δ = 2.65 (s, 3H), 7.71 (s, 4H), 8.53/9.02 (d, *J* = 2.0 Hz, 1H/1H), 11.65 (s-br, 1H) ppm. $^{13}C\{^1H\}$ NMR (100 MHz, $CDCl_3$): δ = 12.8 (CH_3), 114.5 (C), 126.4 (C), 128.1 (C), 131.5 (CH), 132.1 (CH), 132.7 (CH), 136.4 (C), 145.0 (C), 151.2 (CH), 153.6 (C), 193.9 (C) ppm. HRMS (ESI) m/z: $[M + H]^+$ calcd. for $C_{14}H_{11}{}^{79}BrN_3O^+$ 316.0080; found 316.0095.

*(4-Methoxyphenyl)(3-methyl-1H-pyrazolo[3,4-b]pyridin-5-yl)methanone (**2d**).* By the general

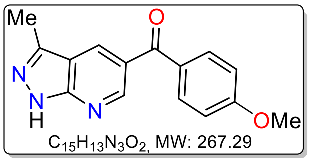

procedure from aldehyde **1d,** (133 mg, 0.5 mmol), **2d** was obtained as a yellow solid (84 mg, 63%). Mp 169–170 °C.^{1}H NMR (400 MHz, $CDCl_3$): δ = 2.66 (s, 3H), 3.91 (s, 3H), 7.02 (d, *J* = 8.0 Hz, 2H), 7.85 (d, *J* = 8.0 Hz, 2H), 8.54 (d, *J* = 2.0 Hz, 1H), 9.02 (d, *J* = 2.0 Hz, 1H), 12.49 (s-br, 1H). $^{13}C\{^1H\}$ NMR (100 MHz, $CDCl_3$): δ = 12.8 (CH_3), 55.7 (CH_3), 114.0 (CH), 114.5 (C), 127.3 (C), 130.2 (C), 132.6 (CH), 144.6 (C), 151.0 (CH), 153.4 (C), 163.6 (C), 193.6 (C) ppm. HRMS (ESI) m/z: $[M + H]^+$ calcd. for $C_{15}H_{14}N_3O_2^+$ 268.1080; found 268.1079.

*(3-Methyl-1H-pyrazolo[3,4-b]pyridin-5-yl)(pyridin-4-yl)methanone (**2e**).* By the general method

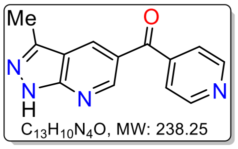

from aldehyde **1e** (119 mg, 0.5 mmol), **2e** was obtained as a yellow solid (77 mg, 65%). Mp 194–196 °C. ^{1}H NMR (400 MHz, $CDCl_3$): δ = 2.67 (s, 3H), 7.63 (d, *J* = 8.0 Hz, 2H), 8.58 (s, 1H), 8.90 (d, *J* = 8.0 Hz, 1H), 9.08 (s, 1H) ppm, 12.81 (s-br, 1H). $^{13}C\{^1H\}$ NMR (100 MHz, $CDCl_3$): δ = 12.8 (CH_3), 114.7 (C), 122.9 (CH), 125.2 (C), 133.2 (CH), 144.5 (C), 145.2 (C), 150.8 (CH), 151.2 (CH), 153.8 (C), 193.5 (C) ppm. HRMS (ESI) m/z: $[M + H]^+$ calcd. for $C_{13}H_{11}N_4O^+$ 239.0927; found 239.0935.

*(3-Methyl-1H-pyrazolo[3,4-b]pyridin-5-yl)(pyridin-2-yl)methanone (**2f**).* By the general way from

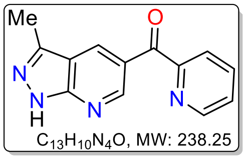

1f (119 mg, 0.5 mmol), **2f** was obtained as a yellow solid (74 mg, 62%). Mp 187–188 °C (amorphous). ^{1}H NMR (400 MHz, $CDCl_3$): δ = 2.66 (s, 3H), 7.55 (td, *J* = 7.78 Hz, 1.72 Hz, 1H), 7.96 (td, *J* = 7.78 Hz, 1.72 Hz, 1H), 8.165 (d, 1H), 8.775 (d, 1H), 8.96 (d, *J* = 1.96 Hz, 1H), 9.42 (d, *J* = 1.96 Hz, 1H) ppm, 12.90 (s-br, 1H). $^{13}C\{^1H\}$ NMR (100 MHz, $CDCl_3$): δ = 12.8 (CH_3), 114.6 (C), 124.9 (CH), 125.5 (C), 126.8

(CH), 134.3 (CH), 137.5 (CH), 145.0 (C), 148.7 (CH), 152.4 (CH), 153.5 (C), 154.8 (C), 191.6 (C) ppm. HRMS (ESI) m/z: $[M + H]^+$ calcd. for $C_{13}H_{11}N_4O^+$ 239.0927; found 239.0929.

*(3-Methyl-1H-pyrazolo[3,4-b]pyridin-5-yl)(thiophen-2-yl)methanone (**2g**)*. By the general method

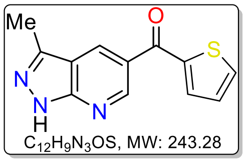

from **1g** (121 mg, 0.5 mmol), **2g** was obtained as a yellow solid (79 mg, 65%). Mp 182–183 °C. ^{1}H NMR (400 MHz, $CDCl_3$): δ = 2.68 (s, 3H), 7.23 (t, J = 4.0 Hz, 1H), 7.72 (d, J = 4.0 Hz, 1H), 7.80 (d, J = 4.0 Hz, 1H), 8.64 (d, J = 1.9 Hz, 1H), 9.15 (d, J = 2.08 Hz, 1H), 12.42 (s-br, 1H) ppm. ^{13}C{^{1}H} NMR (100 MHz, $CDCl_3$): δ = 12.8 (CH_3), 114.6 (C), 127.3 (C), 128.4 (CH), 132.8 (CH), 134.7 (CH), 134.8 (CH), 143.4 (C), 144.7 (C), 150.3 (CH), 153.6 (C), 186.1 (C) ppm. HRMS (ESI) m/z: $[M + H]^+$ calcd. for $C_{12}H_{10}N_3OS^+$ 244.0539; found 244.0541.

*(3-(tert-Butyl)-1H-pyrazolo[3,4-b]pyridin-5-yl)(4-chlorophenyl)methanone (**2h**)*. By the general

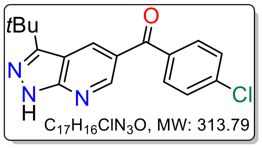

method from **1h** (157 mg, 0.5 mmol), **2h** was obtained as a yellow solid (136 mg, 87%). Mp 203–204 °C. ^{1}H NMR (400 MHz, $CDCl_3$): δ = 1.55 (s, 9H), 7.53 (d, J = 8.0 Hz, 2H), 7.79 (d, J = 8.0 Hz, 2H), 8.76 (s, 1H), 9.02 (s, 1H), 12.79 (s-br, 1H) ppm. ^{13}C{^{1}H} NMR (100 MHz, $CDCl_3$): δ = 30.2 (CH_3), 34.6 (C), 112.7 (C), 125.9 (C), 129.1 (CH), 131.4 (CH), 134.5 (CH), 136.0 (C), 139.4 (C), 150.6 (CH), 154.1 (C), 156.2 (C), 193.7 (C) ppm. HRMS (ESI) m/z: $[M + H]^+$ calcd. for $C_{17}H_{17}ClN_3O^+$ 314.1054; found 314.1049.

*(3-(tert-Butyl)-1H-pyrazolo[3,4-b]pyridin-5-yl)(4-methoxyphenyl)methanone (**2i**)*. By the general

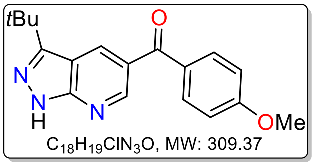

route from **1i** (155 mg, 0.5 mmol), **2i** was obtained as a yellow solid (116 mg, 75%). Mp 1856–186 °C. ^{1}H NMR (400 MHz, $CDCl_3$): δ = 1.56 (s, 9H), 3.92 (s, 3H), 7.02 (d, J = 8.0 Hz, 2H), 7.86 (d, J = 8.0 Hz, 2H), 8.73 (d, J = 2.0 Hz, 1H), 9.02 (d, J = 2.0 Hz, 1H), 12.6 (s-br, 1H) ppm. ^{13}C{^{1}H} NMR (100 MHz, $CDCl_3$): δ = 30.2 (CH_3), 34.5 (C), 55.7 (CH_3), 112.5 (C), 114.0 (CH), 126.8 (C), 130.3 (C), 132.6 (CH), 134.2 (CH), 150.5 (CH), 154.0 (C), 155.9 (C), 163.6 (C), 193.6 (C) ppm. HRMS (ESI) m/z: $[M + H]^+$ calcd. for $C_{18}H_{20}N_3O_2^+$ 310.1550; found 310.1568.

*(4-Chlorophenyl)(3-(4-methoxyphenyl)-1H-pyrazolo[3,4-b]pyridin-5-yl)methanone (**2j**)*. By the

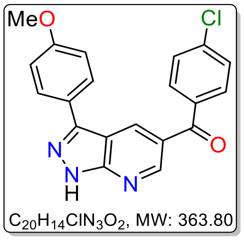

general route from **1j** (181 mg, 0.5 mmol), **2j** was obtained as a yellow solid (149 mg, 82%). Mp 206–207 °C. ^{1}H NMR (400 MHz, $CDCl_3$): δ = 3.89 (s, 3H), 7.06 (d, J = 8.0 Hz, 2H), 7.53 (d, J = 8.0 Hz, 2H), 7.80 (d, J = 8.0 Hz, 2H), 7.91 (d, J = 12.0 Hz, 2H), 8.84/9.07 (d, J = 2.0 Hz, 1H/1H), 12.77 (s-br, 1H) ppm. ^{13}C{^{1}H} NMR (100 MHz, $CDCl_3$): δ = 55.6 (CH_3), 113.0 (C), 114.8 (CH), 124.9 (C), 127.1 (C), 128.7 (CH), 129.2 (CH), 131.5 (CH), 134.0 (CH), 135.8 (C), 139.5 (C), 146.6 (C), 151.0 (CH), 153.9 (C), 160.6 (C), 193.5 (C) ppm. HRMS (ESI) m/z: $[M + H]^+$ calcd. for $C_{20}H_{15}ClN_3O_2^+$ 364.0847; found 364.0859.

(3-(4-Methoxyphenyl)-1H-pyrazolo[3,4-b]pyridin-5-yl)(phenyl)methanone (***2k***). By the general route from **1k** (164 mg, 0.5 mmol), **2k** was obtained as a yellow solid (125 mg, 76%). Mp 206–208 °C. ^{1}H NMR (400 MHz, $CDCl_3$): δ = 3.88 (s, 3H), 7.06 (d, J = 8.0 Hz, 2H), 7.56 (t, J = 8.0 Hz, 2H), 7.67 (t, J = 4.0 Hz, 1H), 7.86 (d, J = 8.0 Hz, 2H), 7.93 (d, J = 8.0 Hz, 2H), 8.89/9.11 (d, J = 2.0 Hz, 1H/1H), 12.71 (s-br, 1H) ppm. ^{13}C{^{1}H} NMR (100 MHz, $CDCl_3$): δ = 55.6 (CH_3), 112.9 (C), 114.7 (CH), 125.0 (C), 127.5 (C), 128.7 (CH), 128.8 (CH), 130.1 (CH), 133.0 (CH), 134.0 (CH), 137.6 (C), 146.7 (C), 151.3 (CH), 153.9 (C), 160.5 (C), 194.8 (C) ppm. HRMS (ESI) m/z: [M + H]$^+$ calcd. for $C_{20}H_{16}N_3O_2^+$ 330.1237; found 330.1248.

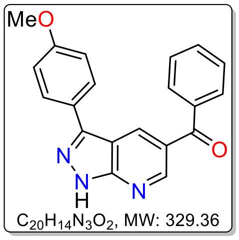

(4-(Diphenylamino)phenyl)(3-methyl-1H-pyrazolo[3,4-b]pyridin-5-yl)methanone (***2l***). By the general way from **1l** (202 mg, 0.5 mmol) but under reflux for 48 h, **2l** was obtained as a bright yellow solid (101 mg, 50%). Mp 181–182 °C. ^{1}H NMR (400 MHz, $CDCl_3$): δ = 2.66 (s, 3H), 7.05 (d, J = 8.7 Hz, 2H), 7.14-7.22 (m, 6H), 7.35 (t, J = 8.0 Hz, 4H), 7.72 (d, J = 8.7 Hz, 2H), 8.56 (s, 1H), 9.04 (s, 1H), 12.41 (s-br, 1H) ppm ^{13}C{^{1}H} NMR (100 MHz, $CDCl_3$): δ = 12.8 (CH_3), 114.6 (C), 119.6 (CH), 125.0 (CH), 126.3 (CH), 127.6 (C), 129.0 (C), 129.8 (CH), 132.0 (CH), 132.4 (CH), 144.5 (C), 146.5 (C), 150.9 (CH), 153.5 (C), 193.1 (C) ppm. HRMS (ESI) m/z: [M + H]$^+$ calcd. for $C_{26}H_{21}N_4O^+$ 405.1710; found 405.1722.

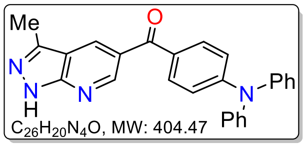

(E)-1-(4-Chlorophenyl)-3-(7-(4-chlorophenyl)-2-methylpyrazolo[1,5-a]pyrimidin-3-yl)prop-2-en-1-one (***2b'***) *& 5-amino-3-methyl-1H-pyrazole-4-carbaldehyde* (***2b''***)*.* By the general route with **1b** (108 mg, 0.5 mmol) but at 70 °C for 0.5 h, **2b'** and **2b''** were obtained as yellow and orange solids and were chromatographed using DCM and DCM/MeOH (50:1) como eluents, respectively. For **2b'**, 17 mg, 17%; mp 260–261 °C (Lit.[2] 259–260 °C); ^{1}H NMR (400 MHz, $CDCl_3$): δ = 2.65 (s, 3H), 7.00 (d, J = 4.0 Hz, 1H) ppm, 7.49 (d, J = 8.0 Hz, 2H) ppm, 7.57 (d, J = 8.0 Hz, 2H) ppm, 8.02–8.09 (m, 5H) ppm, 8.24 (d, J = 16.0 Hz, 1H) ppm, 8.695 (d, J = 4.0 Hz, 1H) ppm. These data matched those previously reported in our research group.[2] For **2b''**, 8 mg, 13%; mp 133–134 °C; ^{1}H NMR (400 MHz, $CDCl_3$): δ = 2.37 (s, 3H), 5.56 (s, 2H) ppm, 9.67 (s, 1H) ppm; ^{13}C{^{1}H} NMR (100 MHz, $CDCl_3$): δ = 10.9 (CH_3), 106.5 (C), 148.0 (C), 154.3 (C), 184.3 (C); HRMS (ESI) m/z: [M + H]$^+$ calcd. for $C_5H_8N_3O^+$ 126.0662; found 126.0659.

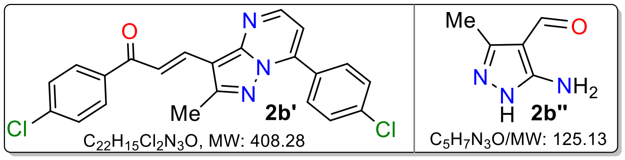

(1,3-Dimethyl-1H-pyrazolo[3,4-b]pyridin-5-yl)(phenyl)methanone (***3a***). By the general procedure from **2a** (118 mg, 0.5 mmol), Cs_2CO_3 (325 mg, 1 mmol), and iodomethane (CH_3I, 141 mg, 1 mmol), **3a** was obtained as a colorless oil-solid (109 mg, 87%). ^{1}H NMR (400 MHz, $CDCl_3$): δ = 2.60 (s, 3H), 4.13 (s, 3H), 7.53 (t, J = 8.0 Hz, 2H), 7.64 (t, J = 7.5 Hz, 1H), 7.81 (d, J = 7.0 Hz, 2H), 8.48 (d, J = 1.96 Hz, 1H), 9.00 (d, J = 1.96 Hz, 1H) ppm. ^{13}C{^{1}H} NMR (100 MHz, $CDCl_3$): δ = 12.6 (CH_3), 33.9 (CH_3), 114.5 (C), 126.1 (C), 128.7 (CH), 130.0 (CH), 132.7 (CH), 132.8 (CH), 137.8 (C), 143.0 (C), 151.2 (CH),

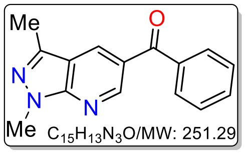

152.0 (C), 195.1 (C) ppm. HRMS (ESI) m/z: $[M + H]^+$ calcd. for $C_{15}H_{14}N_3O^+$ 252.1131; found 252.1142.

*(3-Methyl-1-propyl-1H-pyrazolo[3,4-b]pyridin-5-yl)(phenyl)methanone (**3b**).* By the general way from **2a** (118 mg, 0.5 mmol), Cs_2CO_3 (325 mg, 1 mmol), and *n*-C_3H_7Br (137 mg, 1 mmol), **3b** was obtained as a yellow oil (126 mg, 90%). ^1H NMR (400 MHz, $CDCl_3$): δ = 0.95 (t, *J* = 7.2 Hz, 3H), 1.98 (m, 2H), 2.60 (s, 3H), 4.46 (t, *J* = 7.4 Hz, 2H), 7.53 (t, *J* = 8.0 Hz, 2H), 7.62 (t, *J* = 8.0 Hz, 1H), 7.81 (d, *J* = 8.0 Hz, 2H), 8.47 (d, *J* = 2.0 Hz, 1H) ppm, 8.98 (d, *J* = 2.0 Hz, 1H) ppm. $^{13}C\{^1H\}$ NMR (100 MHz, $CDCl_3$): δ = 11.4 (CH_3), 12.6 (CH_3), 23.3 (CH_2), 48.8 (CH_2), 114.5 (C), 126.1 (C), 128.7 (CH), 130.0 (CH), 132.6 (CH), 132.7 (CH), 137.9 (C), 142.8 (C), 151.0 (CH), 151.9 (C), 195.1 (C) ppm. HRMS (ESI) m/z: $[M + H]^+$ calcd. for $C_{17}H_{18}N_3O^+$ 280.1444; found 280.1444.

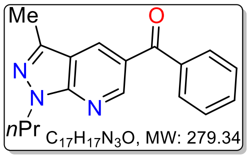

*(1-Butyl-3-methyl-1H-pyrazolo[3,4-b]pyridin-5-yl)(phenyl)methanone (**3c**).* By the general route from **2a** (118 mg, 0.5 mmol), Cs_2CO_3 (325 mg, 1 mmol), and *n*-C_4H_9Br (151 mg, 1 mmol), **3c** was obtained as a yellow oil (126 mg, 86%). ^1H NMR (400 MHz, $CDCl_3$): δ = 0.95 (t, *J* = 7.4 Hz, 3H), 1.36 (m, 2H), 1.94 (m, 2H), 2.59 (s, 3H), 4.49 (t, *J* = 7.2 Hz, 2H), 7.52 (t, *J* = 8.0 Hz, 2H), 7.63 (t, *J* = 8.0 Hz, 1H), 7.81 (d, *J* = 8.0 Hz, 2H), 8.45/8.97 (d, *J* = 2.1 Hz, 1H/1H) ppm. $^{13}C\{^1H\}$ NMR (100 MHz, $CDCl_3$): δ = 12.6 (CH_3), 13.8 (CH_3), 20.1 (CH_2), 31.9 (CH_2), 46.9 (CH_2), 114.4 (C), 126.0 (C), 128.7 (CH), 130.0 (CH), 132.6 (CH), 132.7 (CH), 137.8 (C), 142.8 (C), 151.0 (CH), 151.7 (C), 195.1 (C) ppm. HRMS (ESI) m/z: $[M + H]^+$ calcd. for $C_{18}H_{20}N_3O^+$ 294.1601; found 294.1603.

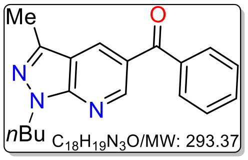

*(1-Butyl-3-methyl-1H-pyrazolo[3,4-b]pyridin-5-yl)(4-chlorophenyl)methanone (**3d**).* By the general way, **3d** was obtained as a yellow oil (139 mg, 85%) from **2b** (136 mg, 0.5 mmol), Cs_2CO_3 (325 mg, 1 mmol), and *n*-C_4H_9Br (151 mg, 1 mmol). ^1H NMR (400 MHz, $CDCl_3$): δ = 0.95 (t, *J* = 7.4 Hz, 3H), 1.35 (m, 2H), 1.93 (m, 2H), 2.59 (s, 3H), 4.48 (t, *J* = 7.2 Hz, 2H), 7.50 (d, *J* = 8.7 Hz, 2H), 7.75 (d, *J* = 8.7 Hz, 2H), 8.42 (d, *J* = 2.0 Hz, 1H), 8.94 (d, *J* = 2.0 Hz, 1H) ppm. $^{13}C\{^1H\}$ NMR (100 MHz, $CDCl_3$): δ = 12.6 (CH_3), 13.8 (CH_3), 20.1 (CH_2), 31.9 (CH_2), 47.0 (CH_2), 114.5 (C), 125.7 (C), 129.0 (CH), 131.4 (CH), 132.5 (CH), 136.1 (C), 139.2 (C), 142.9 (C), 150.8 (CH), 151.8 (C), 193.8 (C) ppm. HRMS (ESI) m/z: $[M + H]^+$ calcd. for $C_{18}H_{19}{}^{35}ClN_3O^+$ 328.1211; found 328.1214.

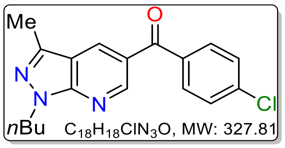

*(1-Benzyl-3-methyl-1H-pyrazolo[3,4-b]pyridin-5-yl)(phenyl)methanone (**3e**)*. By the general route from **2a** (118 mg, 0.5 mmol), Cs_2CO_3 (325 mg, 1 mmol), and benzyl bromide ($PhCH_2Br$, 171 mg, 1 mmol), **3e** was obtained as a yellow solid (147 mg, 90%). Mp 127–128 °C. ^1H NMR (400 MHz, $CDCl_3$): δ = 2.59 (s, 3H), 5.69 (s, 2H), 7.26–7.38 (m, 5H) ppm, 7.53 (t, *J* = 8.0 Hz, 2H), 7.64 (t, *J* = 8.0 Hz, 1H), 7.82 (d, *J* = 8.0 Hz, 2H), 8.48 (d, *J* = 2.1 Hz, 1H), 9.02 (d, *J* = 2.1 Hz, 1H) ppm. $^{13}C\{^1H\}$ NMR (100 MHz, $CDCl_3$): δ = 12.7 (CH_3), 50.8 (CH_2), 114.7 (C), 126.3 (C), 127.9 (CH), 128.0 (CH), 128.6 (CH), 128.8 (CH), 130.0 (CH), 132.6 (C), 132.7 (C), 136.8 (C), 137.7 (C) ppm, 143.5 (C), 151.3 (CH) ppm, 151.9 (C) ppm, 195.0 (C) ppm. HRMS (ESI) m/z: $[M + H]^+$ calcd. for $C_{21}H_{18}N_3O^+$ 328.1444; found 328.1444.

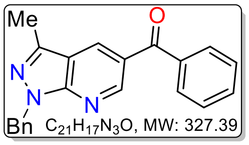

*(1-Benzyl-3-methyl-1H-pyrazolo[3,4-b]pyridin-5-yl)(4-chlorophenyl)methanone **(3f)**.* By the general protocol from **2a** (136 mg, 0.5 mmol), Cs_2CO_3 (325 mg, 1 mmol), and $PhCH_2Br$ (171 mg, 1 mmol), **3f** was obtained as a yellow solid (139 mg, 85%). Mp 181–182 °C. ^{1}H NMR (400 MHz, $CDCl_3$): δ = 2.60 (s, 3H), 5.69 (s, 2H), 7.25–7.38 (m, 5H), 7.51 (d, *J* = 8.0 Hz, 2H), 7.77 (d, *J* = 8.0 Hz, 2H), 8.44 (d, *J* = 2.1 Hz, 1H), 8.98 (d, *J* = 2.1 Hz, 1H) ppm. ^{13}C{^{1}H} NMR (100 MHz, $CDCl_3$): δ = 12.7 (CH_3), 50.9 (CH_2), 114.7 (C), 126.1 (C), 128.0 (CH), 128.1 (CH), 128.8 (CH), 129.0 (CH), 131.4 (CH), 132.5 (CH), 136.1 (C), 136.8 (C) ppm, 139.3 (C), 143.5 (C) ppm, 151.1 (CH) ppm, 152.0 (C) ppm, 193.8 (C) ppm. HRMS (ESI) m/z: $[M + H]^+$ calcd. for $C_{21}H_{17}{}^{35}ClN_3O^+$ 362.1055; found 362.1068.

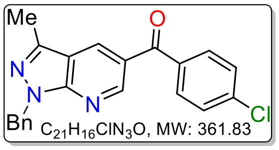

*1-(5-benzoyl-3-methyl-1H-pyrazolo[3,4-b]pyridin-1-yl)ethan-1-one **(3g)**.* By the general protocol from **2b** (59 mg, 0.25 mmol) and acetic anhydride (51 mg, 0.5 mmol) in 2 mL of pyridine, **3g** was obtained as with oil (59 mg, 85%). ^{1}H NMR (400 MHz, $CDCl_3$): δ = 2.65 (s, 3H), δ = 2.90 (s, 3H), 7.54 (t, *J* = 7.8 Hz, 2H), 7.67 (t, *J* = 7.5 Hz, 1H), 7.83 (d, *J* = 8.0 Hz, 2H), 8.53 (s, 1H), 9.13 (s, 1H) ppm ^{13}C{^{1}H} NMR (100 MHz, $CDCl_3$): δ = 12.9 (CH_3), δ = 24.4 (CH_3), 118.4 (C), 128.9 (CH), 129.5 (C), 130.2 (CH), 132.0 (CH), 133.5 (CH), 136.9 (C), 147.9 (C), 152.5 (CH), 153.1 (C), 194.2 (C) ppm. HRMS (ESI) m/z: $[M + H]^+$ calcd. for $C_{16}H_{14}N_3O_2^+$ 280.1081 $[M + H]^+$; found 280.1081.

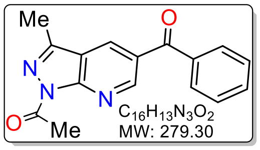

*(1-Butyl-3-methyl-1H-pyrazolo[3,4-b]pyridin-5-yl)(phenyl)methanol **(4a)**.* By the general procedure from **3c** (73 mg, 0.25 mmol) and $NaBH_4$ (18 mg, 0.5 mmol), **4g** was obtained as with oil (59 mg, 87%). ^{1}H NMR (400 MHz, $CDCl_3$): δ = 0.83 (t, *J* = 7.4 Hz, 3H), 1.22 (m, 2H), 1.77 (m, 2H), 2.41 (s, 3H), 3.44 (s, 1H), 4.30 (t, *J* = 7.2 Hz, 2H), 5.90 (s, 1H), 7.19 (m, 1H), 7.24-7.32 (m, 4H), 8.88 (d, *J* = 2.1 Hz, 1H) ppm, 8.34 (d, *J* = 2.1 Hz, 1H) ppm. ^{13}C{^{1}H} NMR (100 MHz, $CDCl_3$): δ = 12.5 (CH_3), 13.8 (CH_3), 20.1 (CH_2), 32.0 (CH_2), 46.8 (CH_2), 74.4 (CH-OH), 114.8 (C), 126.5 (CH), 127.3 (CH), 127.9 (CH), 128.8 (CH), 131.9 (C), 140.6 (C), 143.7 (C), 148.4 (CH), 150.3 (C) ppm. HRMS (ESI) m/z: $[M + H]^+$ calcd. for $C_{18}H_{22}N_3O^+$ 296.1757; found 296.1765.

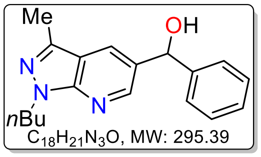

*(1-Butyl-3-methyl-1H-pyrazolo[3,4-b]pyridin-5-yl)(4-chlorophenyl)methanol **(4b)**.* By the general procedure from **3d** (82 mg, 0.25 mmol) and $NaBH_4$ (18 mg, 0.5 mmol), **4g** was obtained as a withe oil (59 mg, 80%).^{1}H NMR (400 MHz, $CDCl_3$): δ = 0.90 (t, *J* = 7.4 Hz, 3H), 1.31 (m, 2H), 1.85 (m, 2H), 2.49 (s, 3H), 3.18 (s, 1H), 4.40 (t, *J* = 7.3 Hz, 2H), 5.97 (s, 1H), 7.33 (m, 4H), 7.91 (s, 1H), 8.42 (s, 1H) ppm. ^{13}C{^{1}H} NMR (100 MHz, $CDCl_3$): δ = 12.5 (CH_3), 13.8 (CH_3), 20.1 (CH_2), 32.1 (CH_2), 46.8 (CH_2), 73.8 (CH-OH), 114.8 (C), 127.4 (CH), 127.9 (CH), 128.9 (CH), 131.4 (C), 133.7 (C), 140.6 (C), 142.1 (C), 148.3 (CH), 150.5 (C) ppm. HRMS (ESI) m/z: $[M + H]^+$ calcd. for $C_{18}H_{21}{}^{35}ClN_3O^+$ 330.1368; found 330.1368.

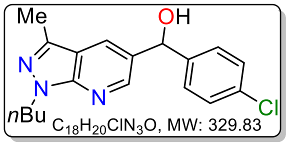

4. HRMS analysis

User Chromatograms

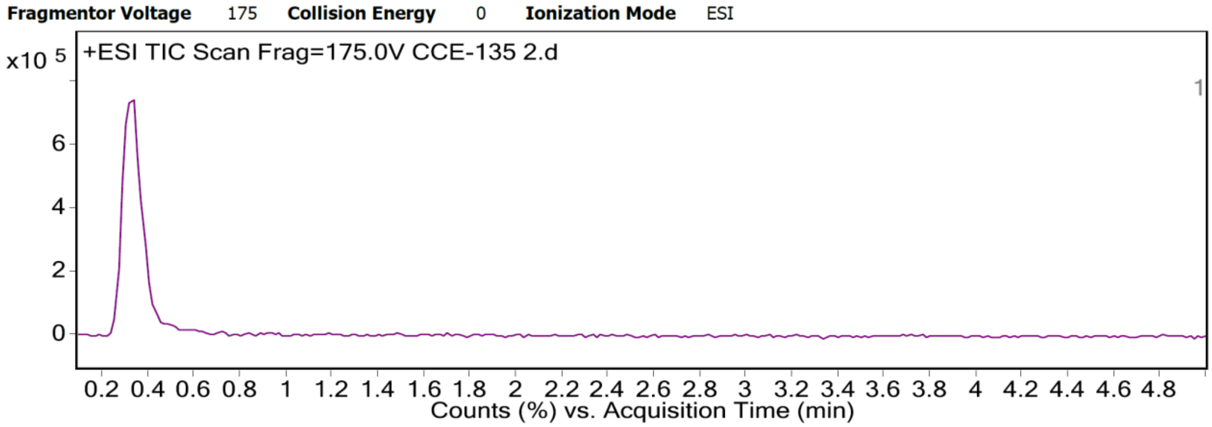

User Spectra

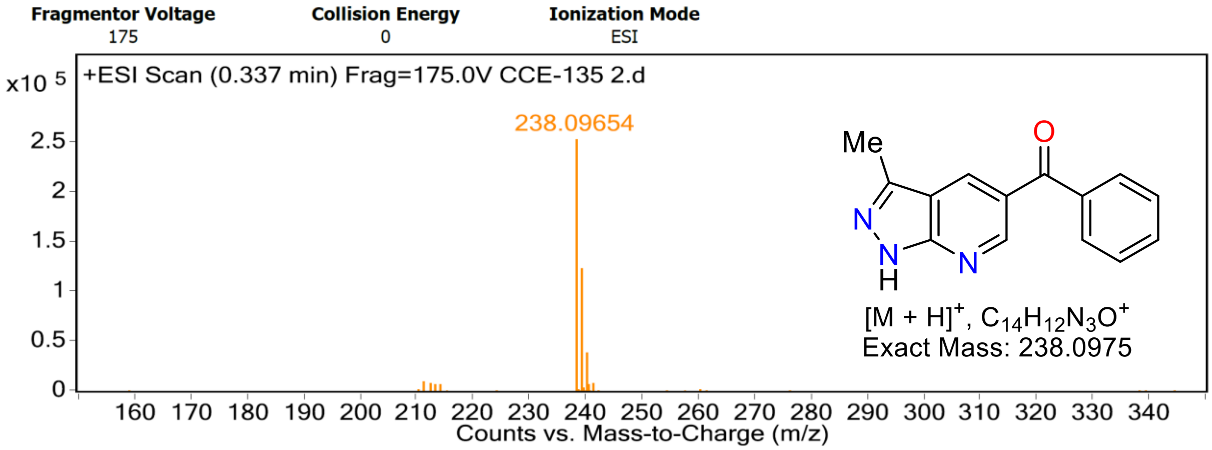

Peak List

m/z	z	Abund	Abund %
238.09654		252600.8	100
238.13568		36321.8	14.38
239.1017	1	123711.5	48.98
240.10758	1	38727.1	15.33

Fig. S2 HRMS analysis of (3-methyl-1*H*-pyrazolo[3,4-*b*]pyridin-5-yl)(phenyl)methanone (**2a**)

User Chromatograms

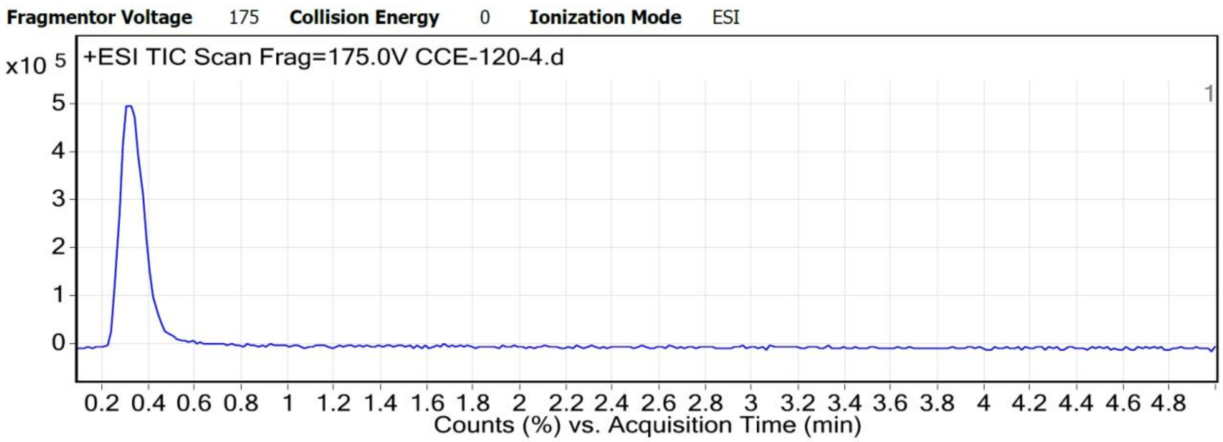

User Spectra

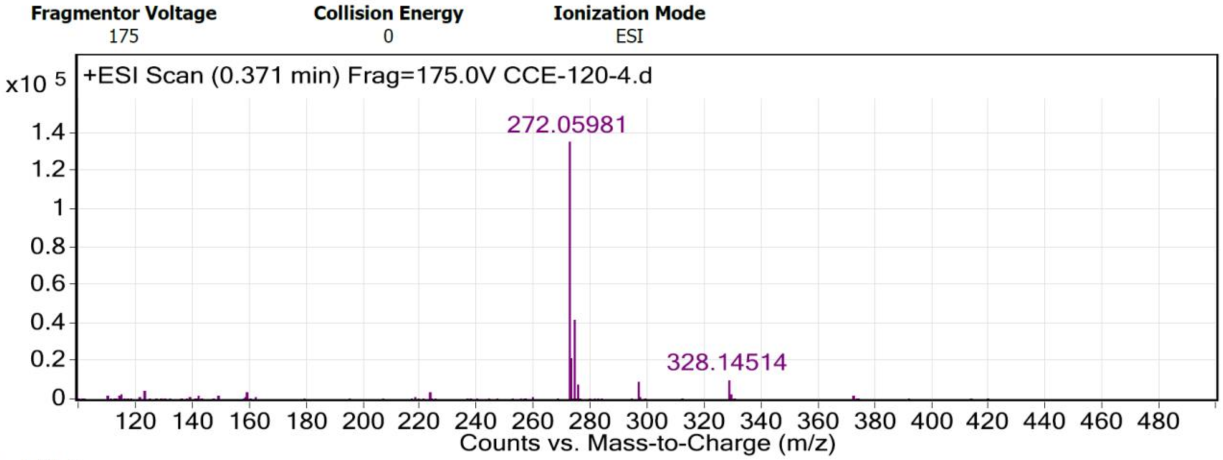

Peak List

m/z	z	Abund	HeightPercent
81.93703		7785.2	5.73
272.05981	1	135961	100
273.06076	1	21763.7	16.01
274.05729	1	42314.1	31.12
275.05924	1	8118.7	5.97
296.17686		9455.3	6.95
328.14514		10893.7	8.01

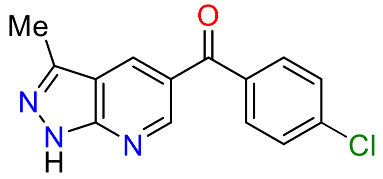

$[M + H]^+$, $C_{14}H_{11}{}^{35}ClN_3O^+$
Exact Mass: 272.0585

Fig. S3 HRMS analysis of (4-chlorophenyl)(3-methyl-1*H*-pyrazolo[3,4-b]pyridin-5-yl)methanone (**2b**)

User Chromatograms

Fragmentor Voltage 175 **Collision Energy** 0 **Ionization Mode** ESI

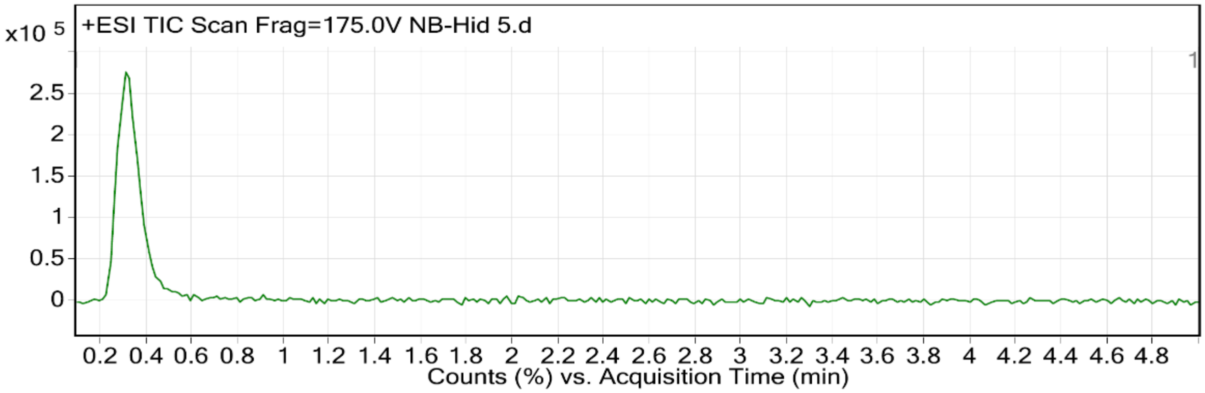

User Spectra

Fragmentor Voltage 175 **Collision Energy** 0 **Ionization Mode** ESI

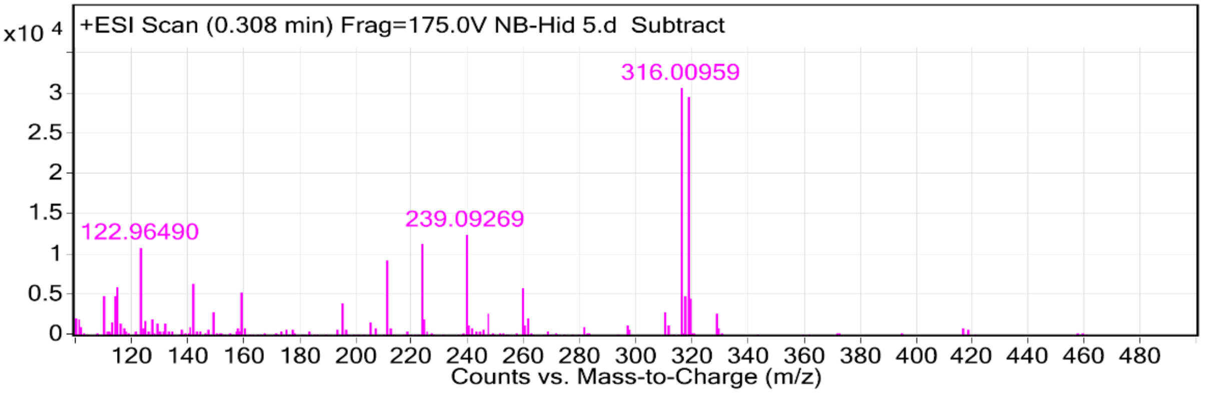

Peak List

m/z	z	Abund	HeightPercent
195.0823		4012.3	13.08
211.09774		9284.1	30.26
223.07509	1	11369.6	37.06
239.09269		12457.6	40.61
259.05347		5963.9	19.44
316.00959	1	30679.6	100
317.01155	1	4893.2	15.95
318.00613	1	29496.1	96.14
319.01014	1	4542.8	14.81

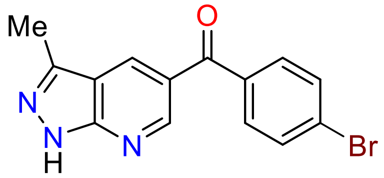

$[M + H]^+$, $C_{14}H_{11}{}^{79}BrN_3O^+$
Exact Mass: 316.0080

Fig. S4 HRMS analysis of (4-bromophenyl)(3-methyl-1*H*-pyrazolo[3,4-*b*]pyridin-5-yl)methanone (**2c**)

User Chromatograms

Fragmentor Voltage 175 **Collision Energy** 0 **Ionization Mode** ESI

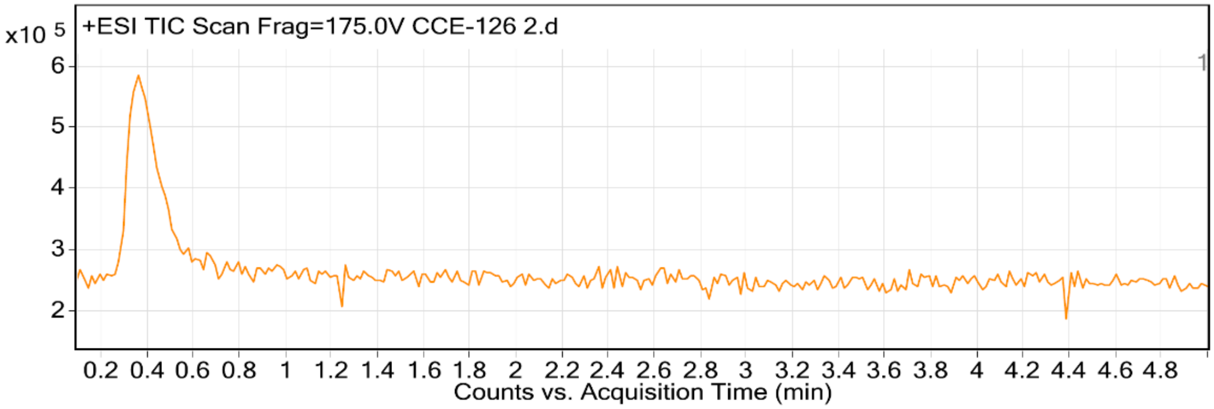

User Spectra

Fragmentor Voltage	Collision Energy	Ionization Mode
175	0	ESI

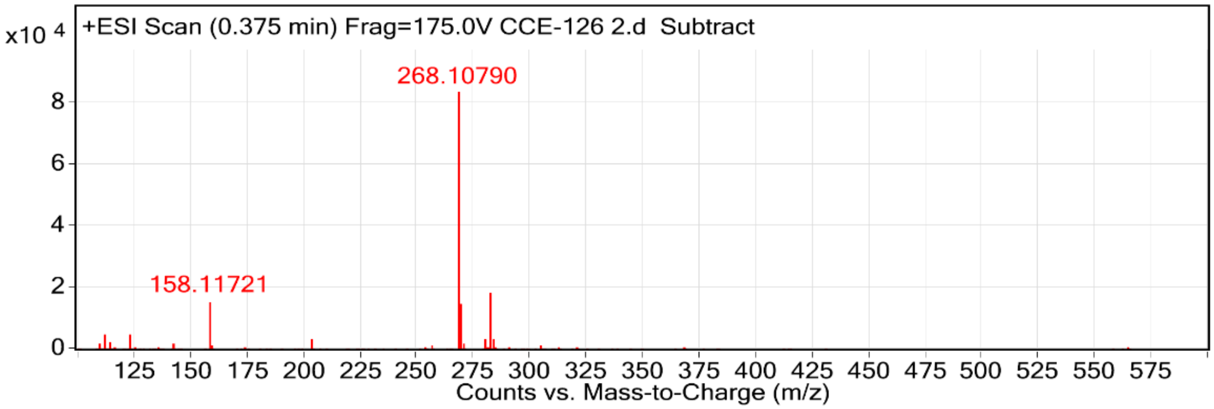

Peak List

m/z	z	Abund	HeightPercent
81.9382		6841.2	8.2
112.11186		5086.7	6.09
122.96363		4893.3	5.86
158.11721		15725.4	18.84
268.1079	1	83473.6	100
269.1105	1	14939	17.9
282.27824		18688.3	22.39
609.2804	1	20562.1	24.63

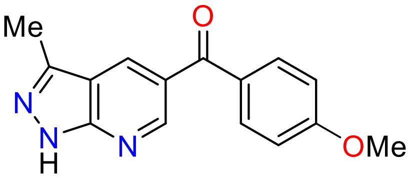

$[M + H]^+$, $C_{15}H_{14}N_3O_2^+$
Exact Mass: 268.1081

Fig. S5 HRMS analysis of (4-methoxyphenyl)(3-methyl-1*H*-pyrazolo[3,4-*b*]pyridin-5-yl)methanone (**2d**)

User Chromatograms

Fragmentor Voltage 175 **Collision Energy** 0 **Ionization Mode** ESI

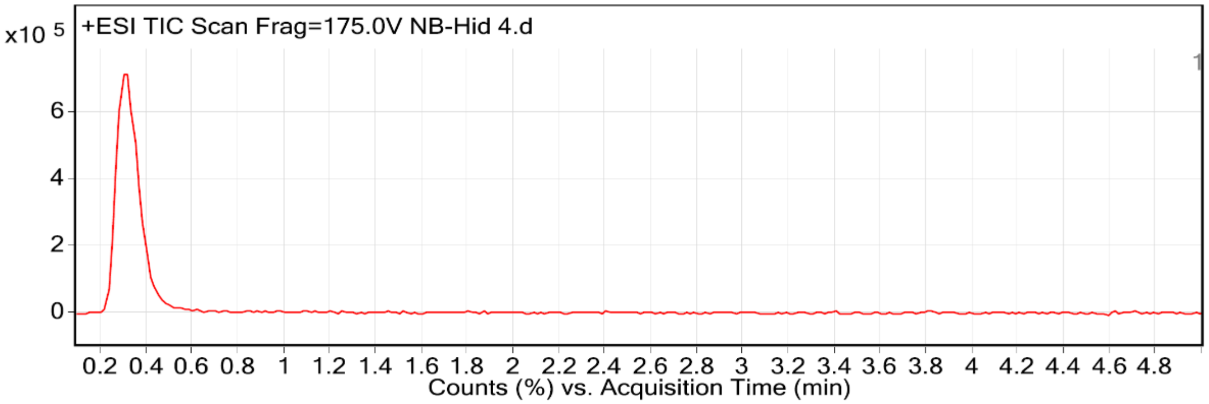

User Spectra

Fragmentor Voltage 175 **Collision Energy** 0 **Ionization Mode** ESI

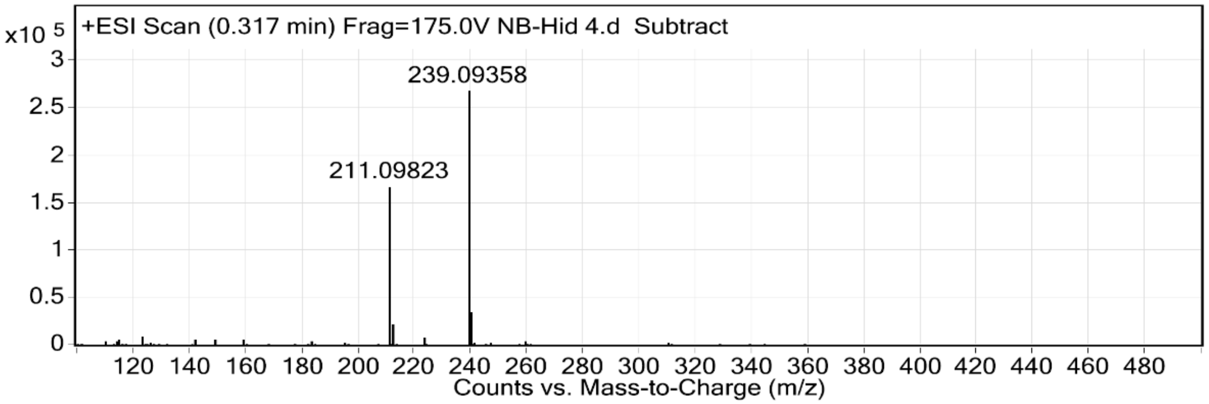

Peak List

m/z	z	Abund	HeightPercent
81.93688		16723.4	6.22
211.09823	1	167462.4	62.33
212.10117	1	22798.1	8.49
239.09358	1	268675.7	100
240.09667	1	35983.7	13.39

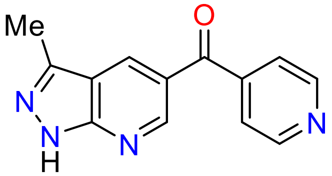

$[M + H]^+$, $C_{13}H_{11}N_4O^+$
Exact Mass: 239.0927

Fig. S6 HRMS analysis of (3-methyl-1*H*-pyrazolo[3,4-*b*]pyridin-5-yl)(pyridin-4-yl)methanone (**2e**)

User Chromatograms

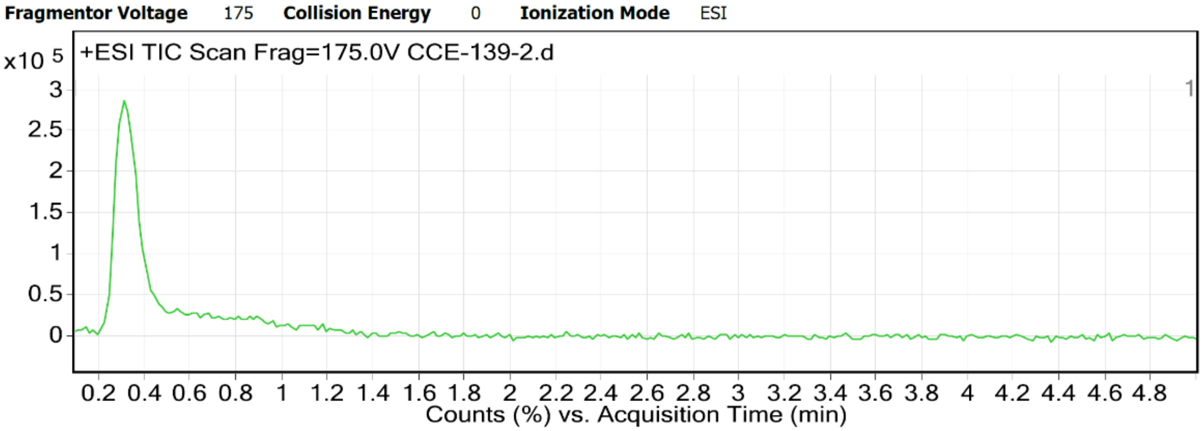

User Spectra

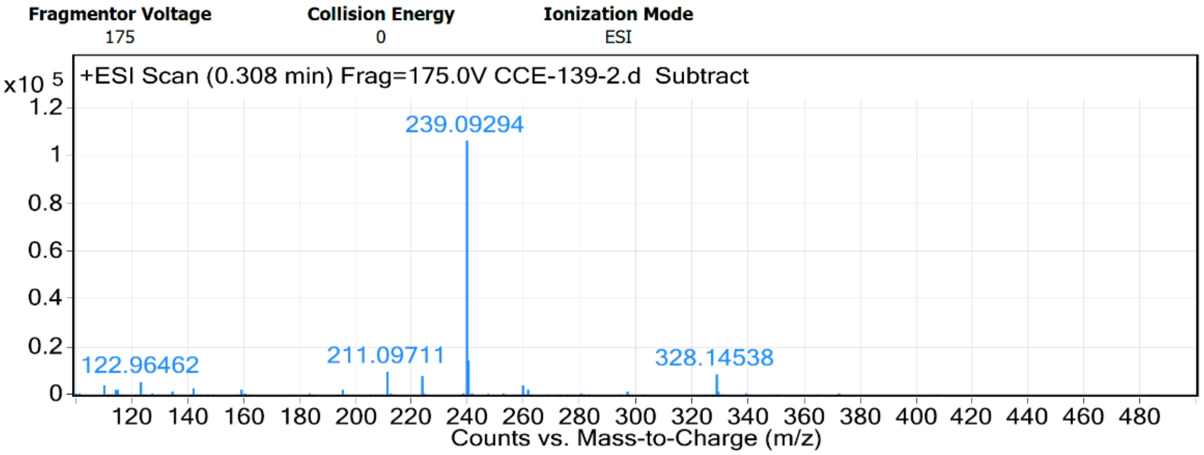

Peak List

m/z	z	Abund	HeightPercent
81.93675		9578.3	8.98
122.96462		5823.6	5.46
211.09711		9940	9.32
223.07488		8268.9	7.75
239.09294	1	106676	100
240.09581	1	14715.1	13.79
328.14538		8916.1	8.36

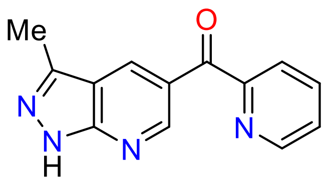

$[M + H]^+$, $C_{13}H_{11}N_4O^+$
Exact Mass: 239.0927

Fig. S7 HRMS analysis of (3-methyl-1*H*-pyrazolo[3,4-*b*]pyridin-5-yl)(pyridin-2-yl)methanone (**2f**)

User Chromatograms

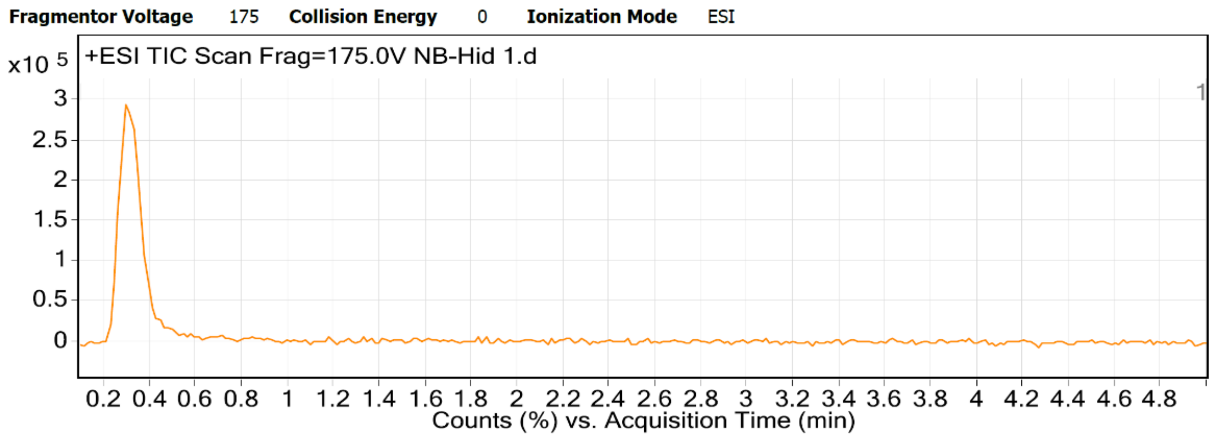

User Spectra

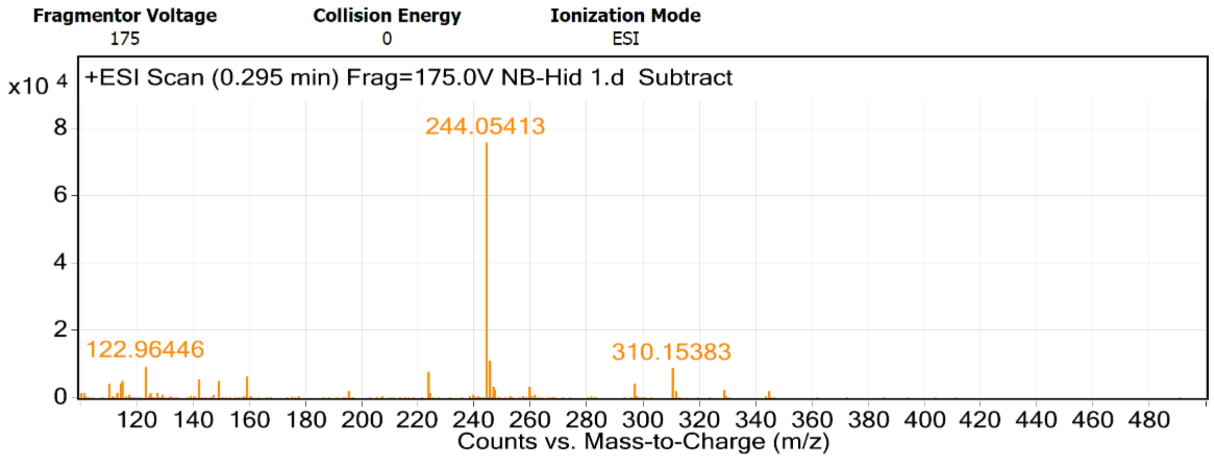

Peak List

m/z	z	Abund	HeightPercent
141.95907		5954.4	7.85
149.02333		5299.8	6.99
158.97637		7020.7	9.26
223.07438		8164.7	10.77
244.05413	1	75838.6	100
245.05848	1	11327	14.94
296.17508		4524.3	5.97
310.15383		8902.4	11.74

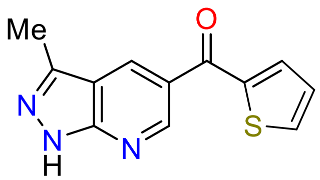

$[M + H]^+$, $C_{12}H_{10}N_3OS^+$
Exact Mass: 244.0539

Fig. S8 HRMS analysis of (3-methyl-1*H*-pyrazolo[3,4-*b*]pyridin-5-yl)(thiophen-2-yl)methanone (**2g**)

User Chromatograms

Fragmentor Voltage 175 **Collision Energy** 0 **Ionization Mode** ESI

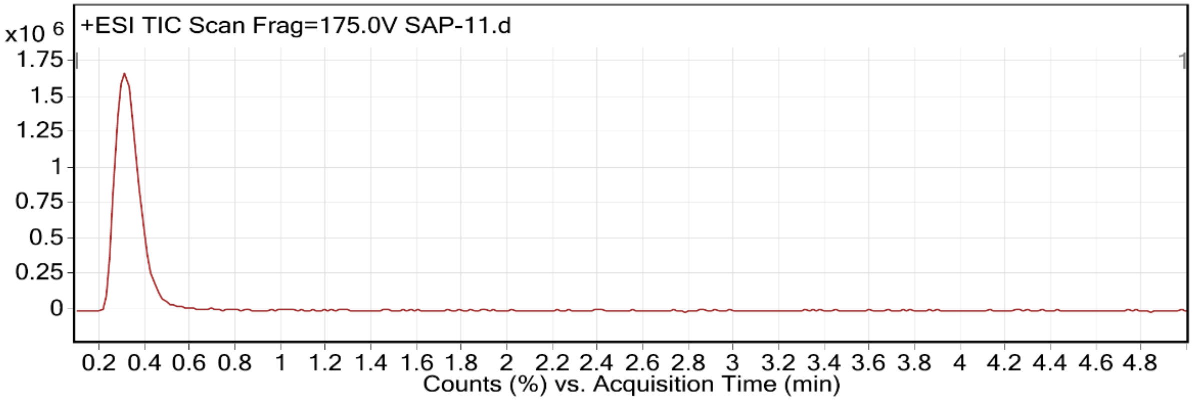

User Spectra

**Fragmentor Voltage**	**Collision Energy**	**Ionization Mode**
175	0	ESI

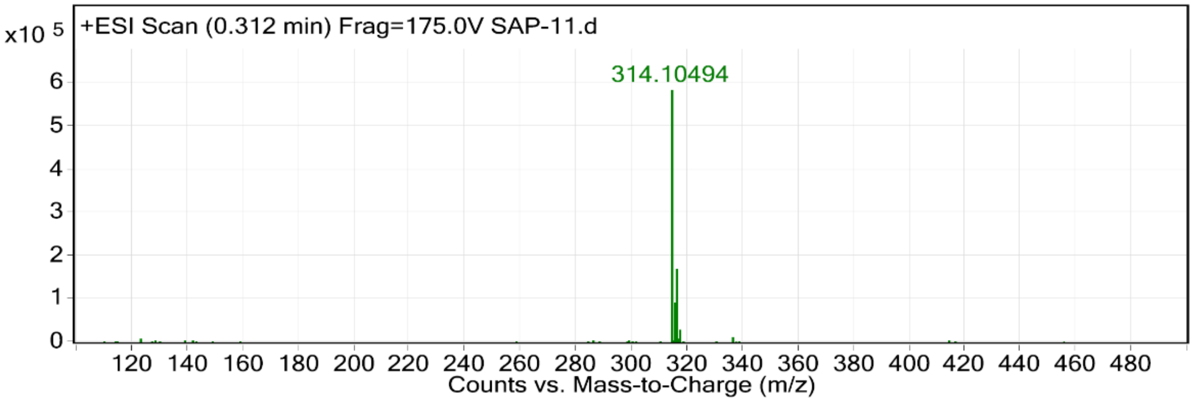

Peak List

m/z	z	Abund	HeightPercent
314.10494	1	584712.6	100
314.14786		68972.9	11.8
315.10839	1	95871	16.4
316.10341	1	170714.2	29.2
317.10596	1	30743.4	5.26

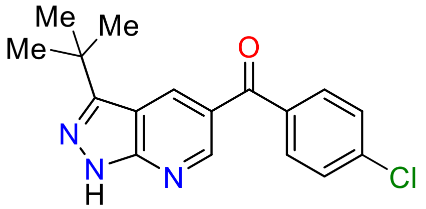

$[M + H]^+$, $C_{17}H_{17}{}^{35}ClN_3O^+$
Exact Mass: 314.1055

Fig. S9 HRMS analysis of (3-(*t*-butyl)-1*H*-pyrazolo[3,4-*b*]pyridin-5-yl)(4-chlorophenyl)methanone (**2h**)

User Chromatograms

Fragmentor Voltage 175 **Collision Energy** 0 **Ionization Mode** ESI

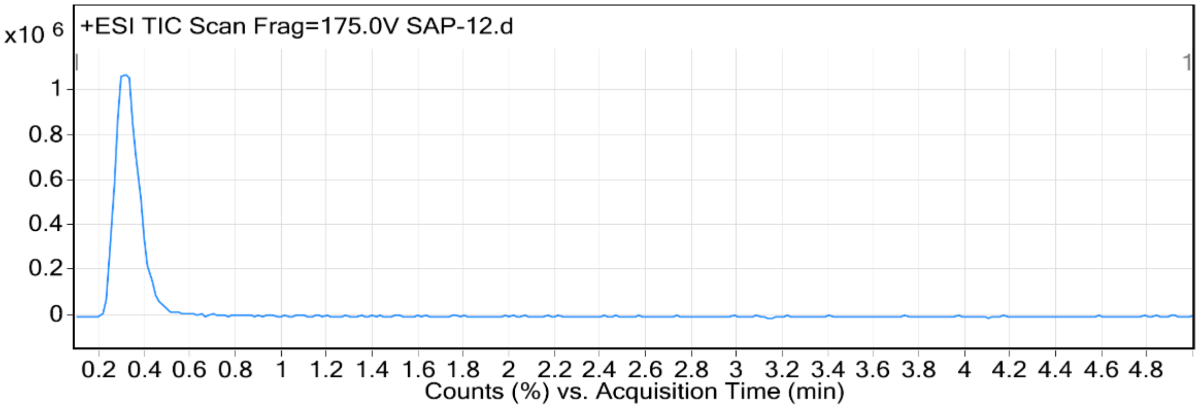

User Spectra

**Fragmentor Voltage**	**Collision Energy**	**Ionization Mode**
175	0	ESI

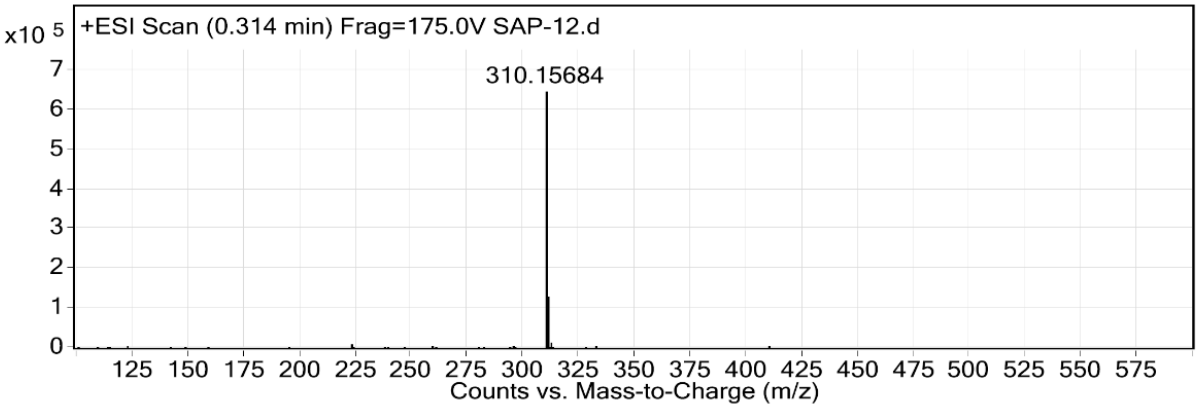

Peak List

m/z	z	Abund	HeightPercent
310.15684	1	646219.4	100
311.15974	1	131675.3	20.38

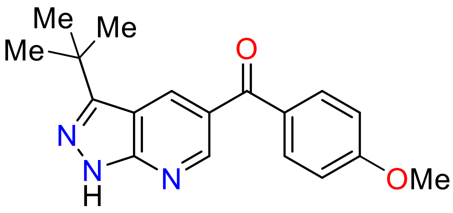

$[M + H]^+$, $C_{18}H_{20}N_3O_2^+$
Exact Mass: 310.1550

Fig. S10 HRMS analysis of (3-(*t*-butyl)-1*H*-pyrazolo[3,4-*b*]pyridin-5-yl)(4-methoxyphenyl)methanone (**2i**)

User Chromatograms

Fragmentor Voltage 175 **Collision Energy** 0 **Ionization Mode** ESI

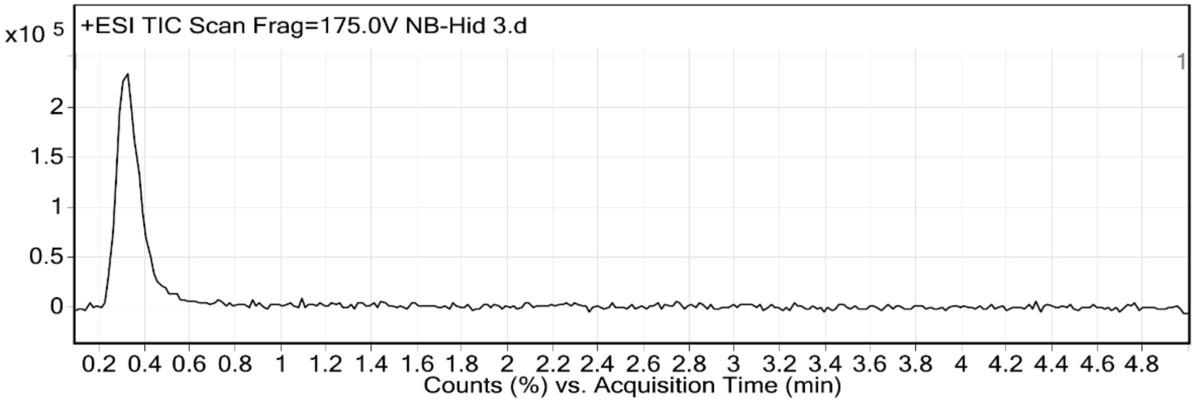

User Spectra

Fragmentor Voltage 175 **Collision Energy** 0 **Ionization Mode** ESI

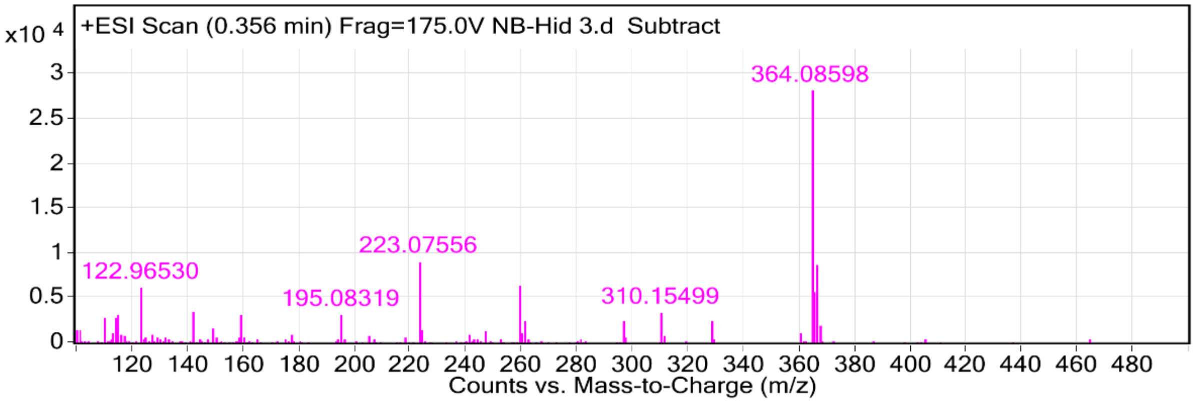

Peak List

m/z	z	Abund	HeightPercent
195.08319		3203.3	11.35
223.07556	1	9149.2	32.41
259.05351		6383.6	22.61
261.05024		2594.3	9.19
296.17747		2562	9.07
310.15499		3451.4	12.23
328.14684		2484	8.8
364.08598	1	28231.9	100
365.08884	1	5703.9	20.2
366.08342	1	8835.2	31.29

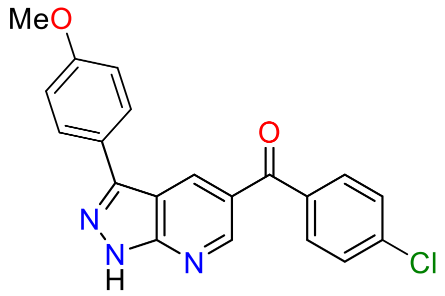

$[M + H]^+$, $C_{20}H_{15}{}^{35}ClN_3O_2{}^+$
Exact Mass: 364.0847

Fig. S11 HRMS analysis of (4-chlorophenyl)(3-(4-methoxyphenyl)-1*H*-pyrazolo[3,4-*b*]pyridin-5-yl)methanone (**2j**)

User Chromatograms

Fragmentor Voltage 175 **Collision Energy** 0 **Ionization Mode** ESI

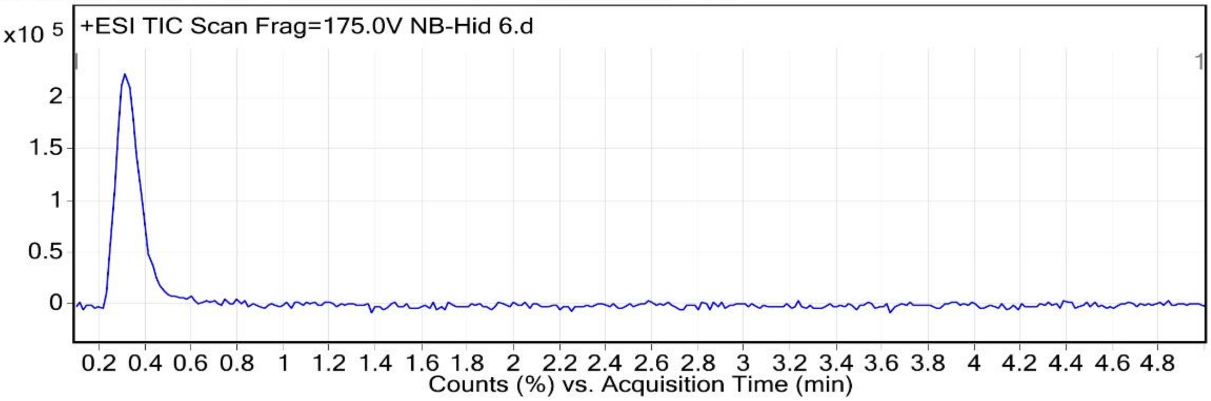

User Spectra

**Fragmentor Voltage**	**Collision Energy**	**Ionization Mode**
175	0	ESI

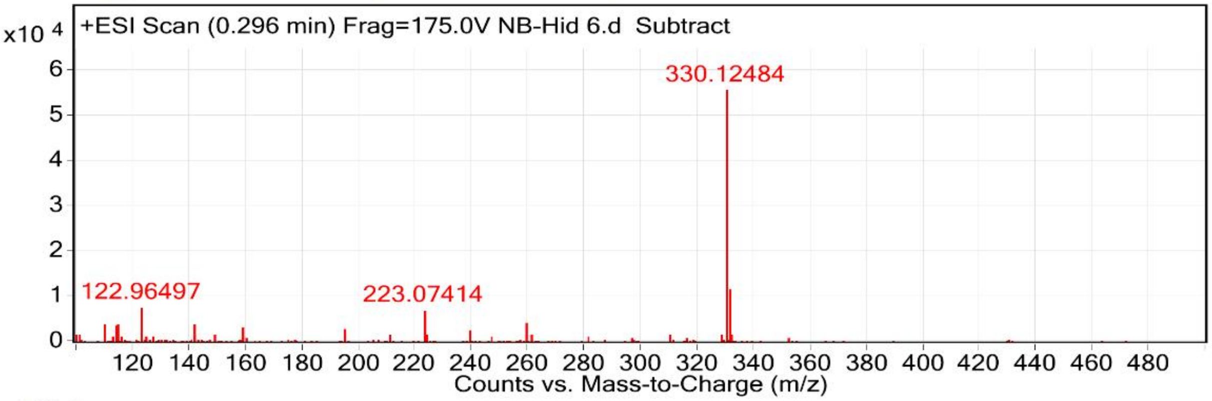

Peak List

m/z	z	Abund	HeightPercent
122.96497		7614.2	13.68
141.95936		4045.5	7.27
158.97393		3451	6.2
195.08218		3001	5.39
223.07414		6981.2	12.55
259.05236		4211.2	7.57
330.12484	1	55647.7	100
331.12801	1	11554.7	20.76

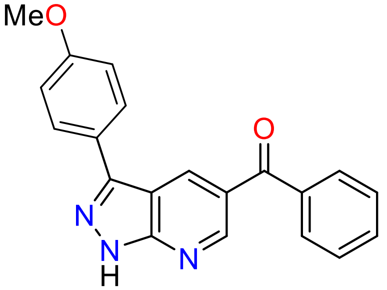

$[M + H]^+$, $C_{20}H_{16}N_3O_2^+$
Exact Mass: 330.1237

Fig. S12 HRMS analysis of (3-(4-methoxyphenyl)-1*H*-pyrazolo[3,4-*b*]pyridin-5-yl)(phenyl)methanone (**2k**)

User Chromatograms

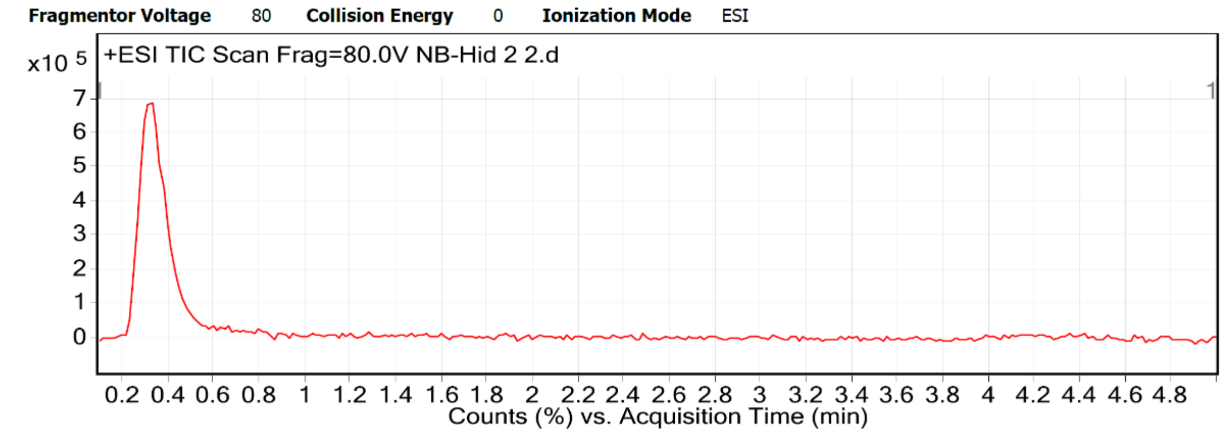

User Spectra

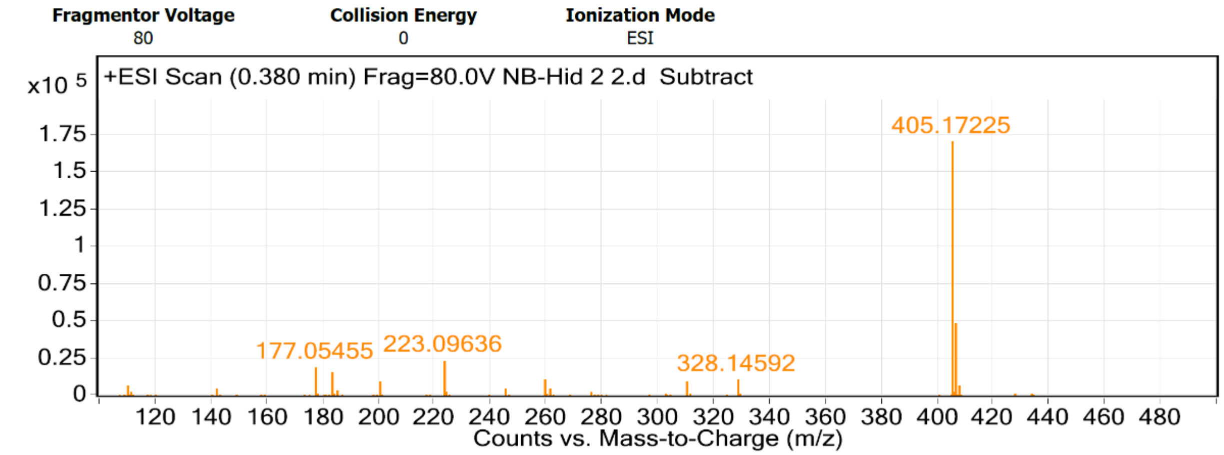

Peak List

m/z	z	Abund	HeightPercent
177.05455		19149.2	11.22
182.9852		16567.3	9.71
200.00094		10308	6.04
223.09636		23822.6	13.96
259.05422		11646.9	6.82
310.1554		10119.4	5.93
328.14592		10850.1	6.36
405.17225	1	170687.3	100

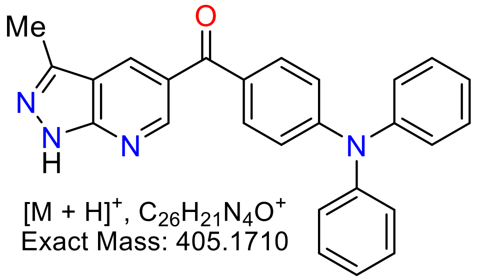

Fig. S13 HRMS analysis of (4-(diphenylamino)phenyl)(3-methyl-1*H*-pyrazolo[3,4-*b*]pyridin-5-yl)methanone (**2l**)

User Chromatograms

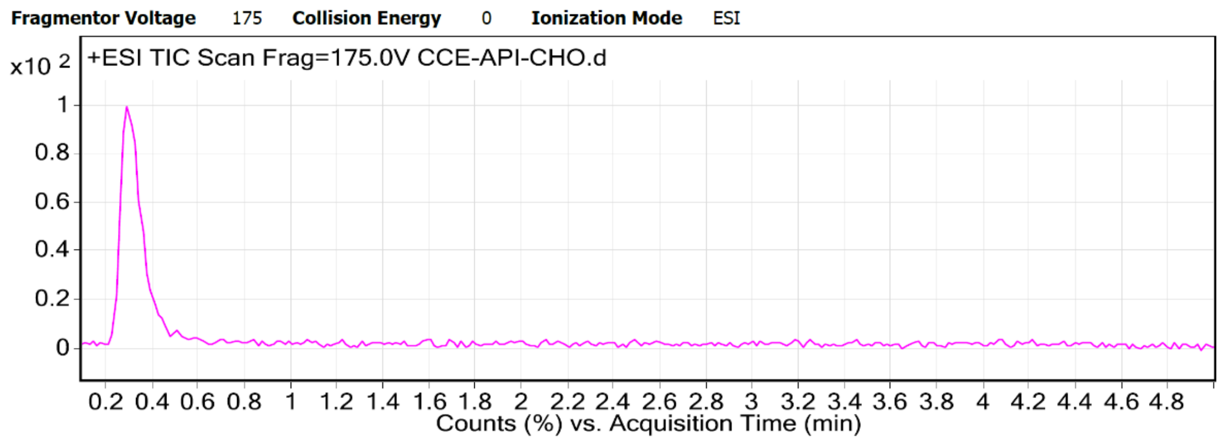

User Spectra

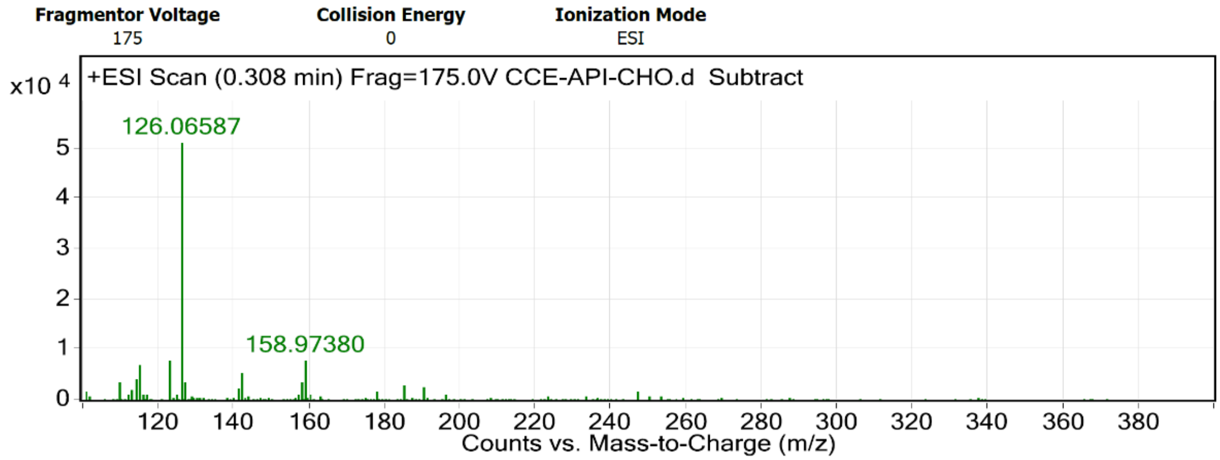

Peak List

m/z	z	Abund	HeightPercent
109.94353		3705.2	7.24
113.96342		4327.2	8.45
114.98395		6985.5	13.65
122.96312		7932.1	15.5
126.06587	1	51190.1	100
127.06941	1	3726.3	7.28
141.95864		5523	10.79
158.11692		3584.1	7

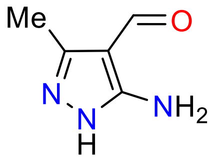

$[M + H]^+$, $C_5H_8N_3O^+$
Exact Mass: 126.0662

Fig. S14 HRMS analysis of 5-amino-3-methyl-1*H*-pyrazole-4-carbaldehyde (**2b''**)

User Chromatograms

Fragmentor Voltage 175 **Collision Energy** 0 **Ionization Mode** ESI

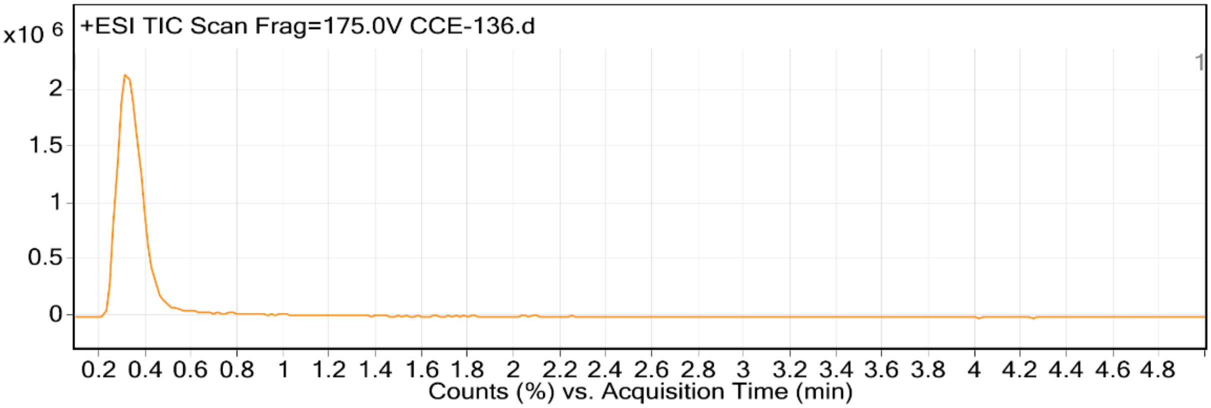

User Spectra

Fragmentor Voltage	Collision Energy	Ionization Mode
175	0	ESI

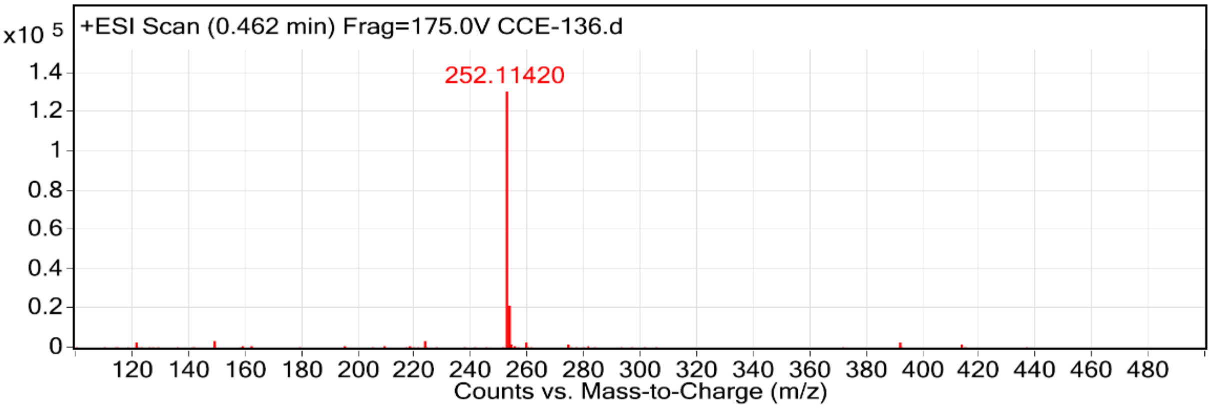

Peak List

m/z	z	Abund	HeightPercent
252.1142	1	130702.5	100
253.11581	1	21813.3	16.69

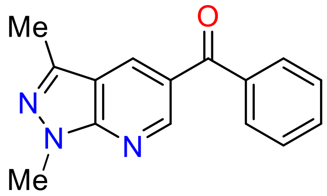

$[M + H]^+$, $C_{15}H_{14}N_3O^+$
Exact Mass: 252.1131

Fig. S15 HRMS analysis of (1,3-dimethyl-1*H*-pyrazolo[3,4-*b*]pyridin-5-yl)(phenyl)methanone (**3a**)

User Chromatograms

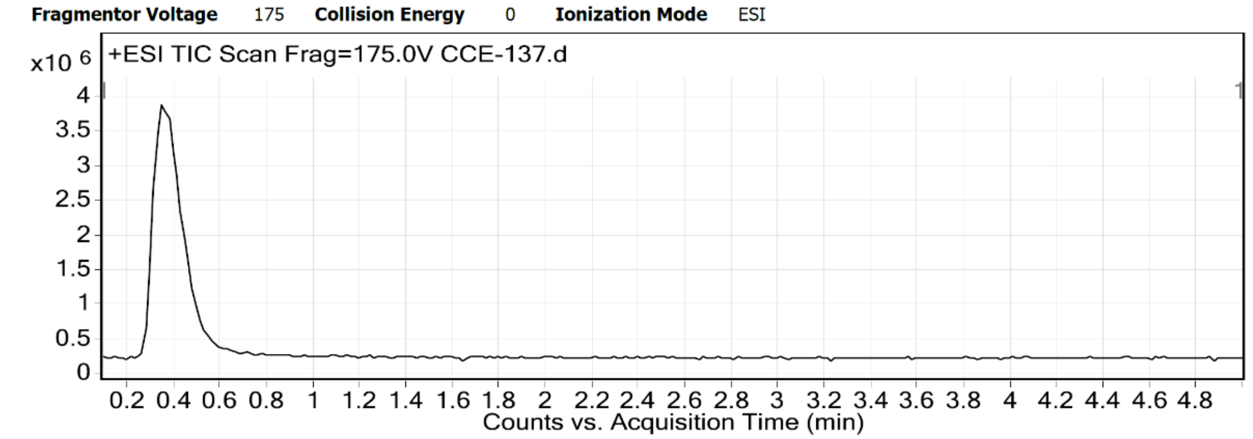

User Spectra

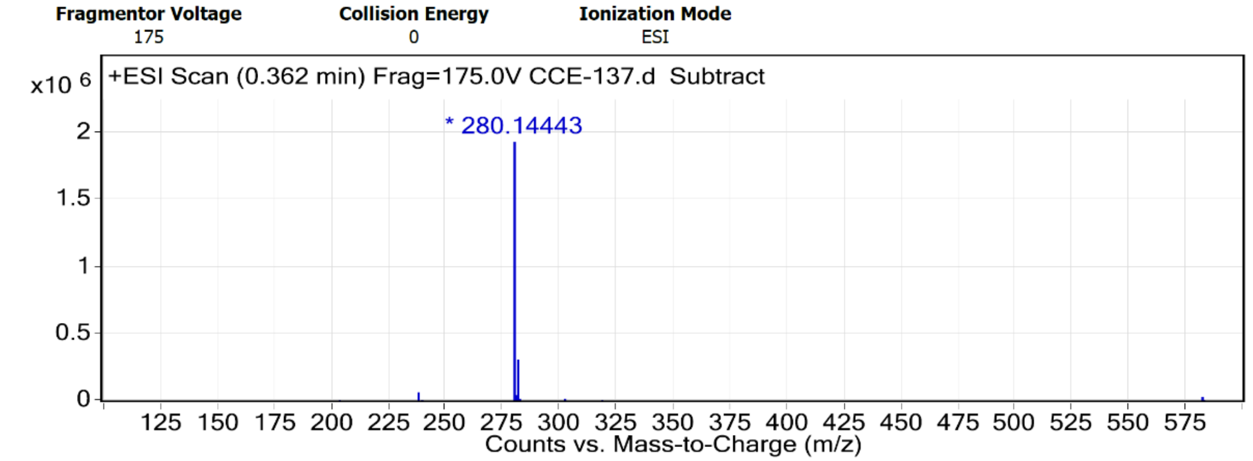

Peak List

m/z	z	Abund	HeightPercent
280.14443	1	1936032.1	100
280.18669		129166.5	6.67
281.14704	1	308474.5	15.93

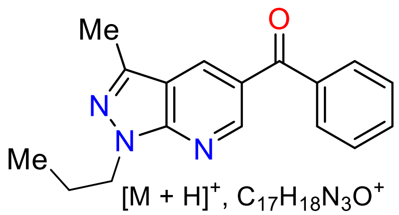

$[M + H]^+$, $C_{17}H_{18}N_3O^+$
Exact Mass: 280.1444

Fig. S16 HRMS analysis of (*3*-methyl-1-propyl-1*H*-pyrazolo[3,4-*b*]pyridin-5-yl)(phenyl)methanone (**3b**)

User Chromatograms

Fragmentor Voltage 175 **Collision Energy** 0 **Ionization Mode** ESI

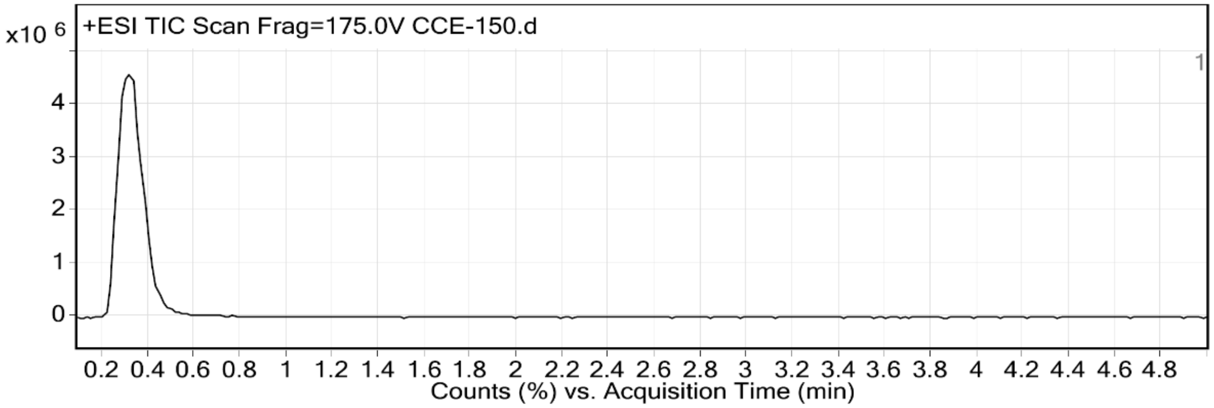

User Spectra

Fragmentor Voltage 175 **Collision Energy** 0 **Ionization Mode** ESI

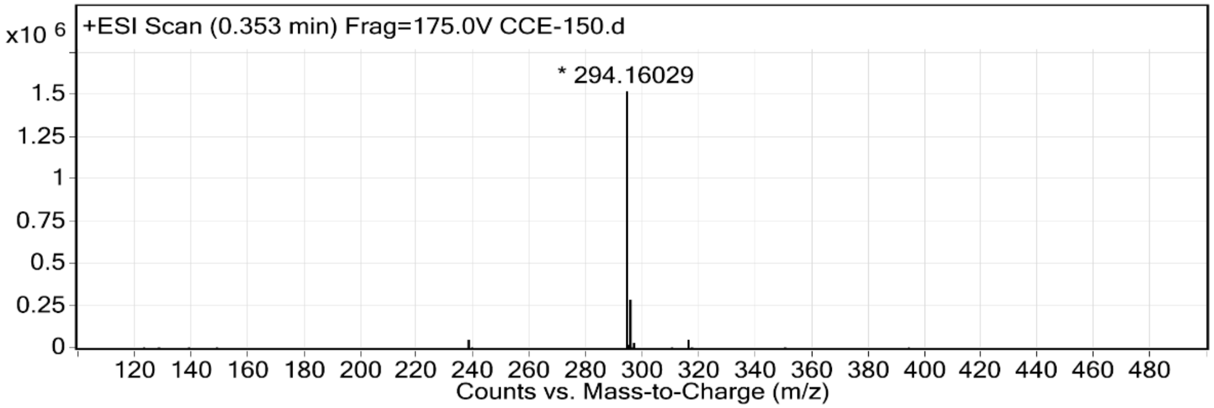

Peak List

m/z	z	Abund	HeightPercent
294.16029	1	1523380.5	100
294.20222		111572.1	7.32
294.24146		80863.7	5.31
295.16294	1	287785.2	18.89

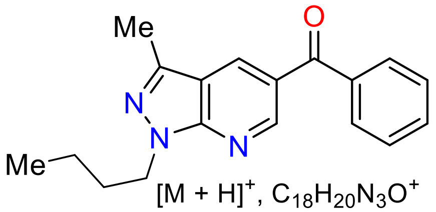

$[M + H]^+$, $C_{18}H_{20}N_3O^+$
Exact Mass: 294.1601

Fig. S17 HRMS analysis of (1-butyl-3-methyl-1*H*-pyrazolo[3,4-*b*]pyridin-5-yl)(phenyl)methanone (**3c**)

User Chromatograms

Fragmentor Voltage 175 **Collision Energy** 0 **Ionization Mode** ESI

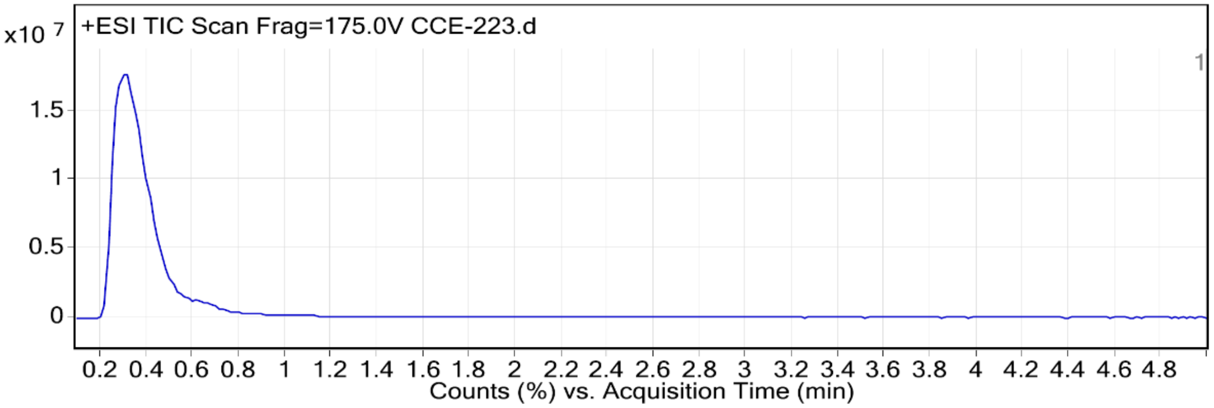

User Spectra

Fragmentor Voltage	Collision Energy	Ionization Mode
175	0	ESI

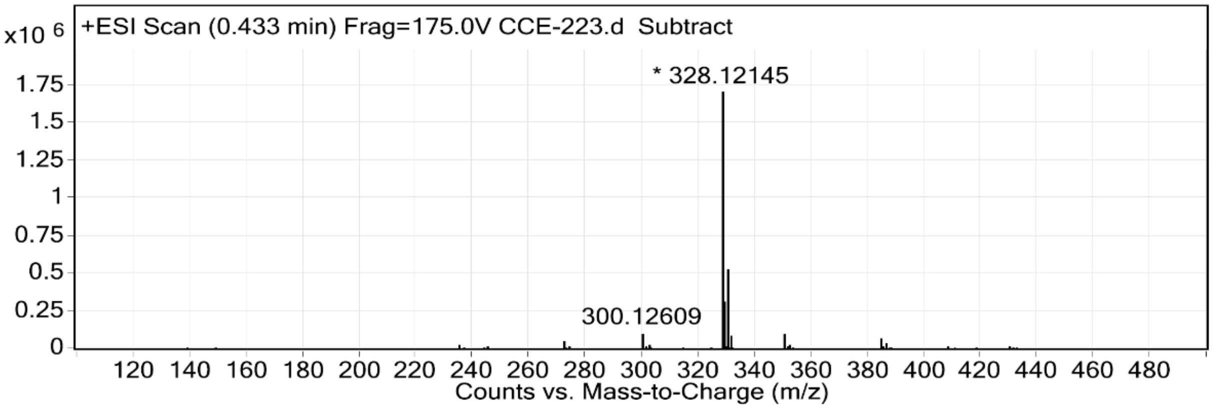

Peak List

m/z	z	Abund	HeightPercent
300.12609		104372.1	6.11
328.12145	1	1707117.5	100
328.16538		126568.4	7.41
328.20785		91290.5	5.35
328.27561		86043.7	5.04
329.1237	1	312547.9	18.31
330.11871	1	529508.2	31.02
331.12179	1	92387.1	5.41

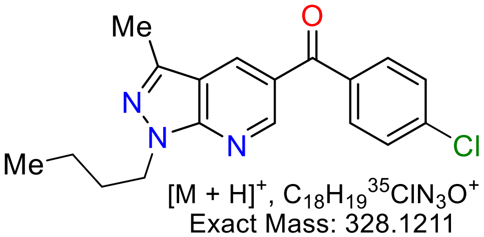

[M + H]$^+$, $C_{18}H_{19}{}^{35}ClN_3O^+$
Exact Mass: 328.1211

Fig. S18 HRMS analysis of (1-butyl-3-methyl-1*H*-pyrazolo[3,4-*b*]pyridin-5-yl)(4-chlorophenyl)methanone (**3d**)

User Chromatograms

Fragmentor Voltage 175 **Collision Energy** 0 **Ionization Mode** ESI

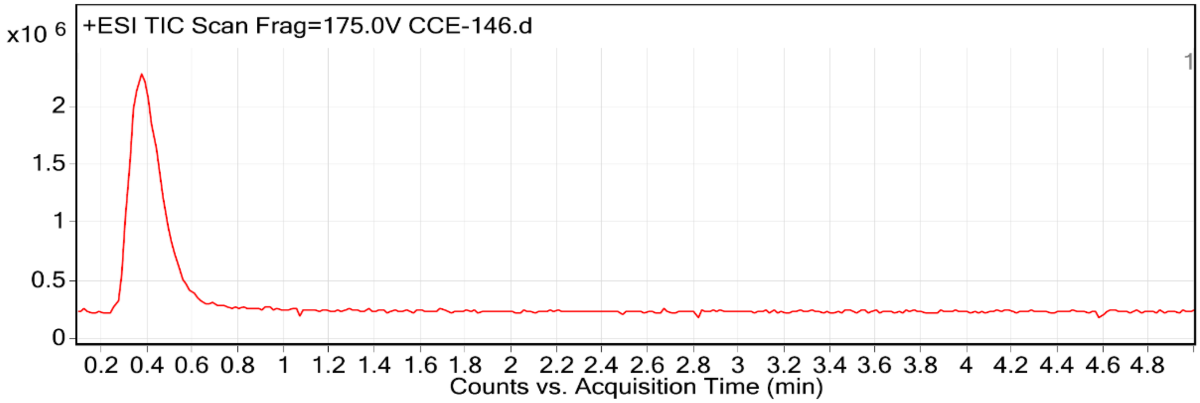

User Spectra

Fragmentor Voltage	Collision Energy	Ionization Mode
175	0	ESI

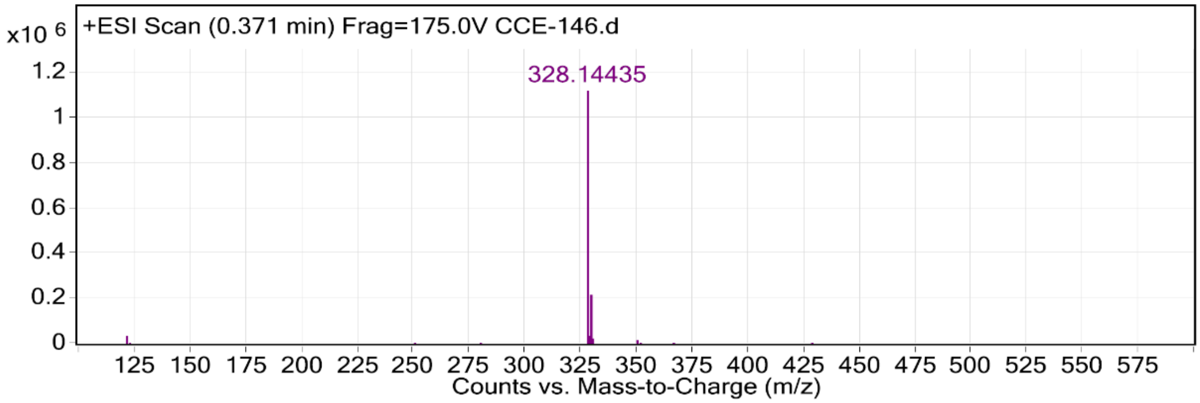

Peak List

m/z	z	Abund	HeightPercent
328.14435	1	1120921	100
328.19066		96727	8.63
329.14746	1	221351	19.75
922.01134		68610	6.12

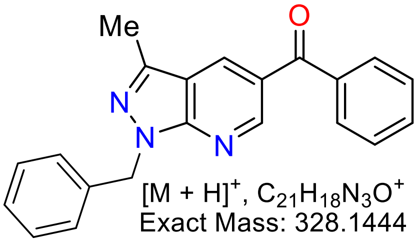

$[M + H]^+$, $C_{21}H_{18}N_3O^+$
Exact Mass: 328.1444

Fig. S19 HRMS analysis of (1-benzyl-3-methyl-1*H*-pyrazolo[3,4-*b*]pyridin-5-yl)(phenyl)methanone (**3e**)

User Chromatograms

Fragmentor Voltage 175 **Collision Energy** 0 **Ionization Mode** ESI

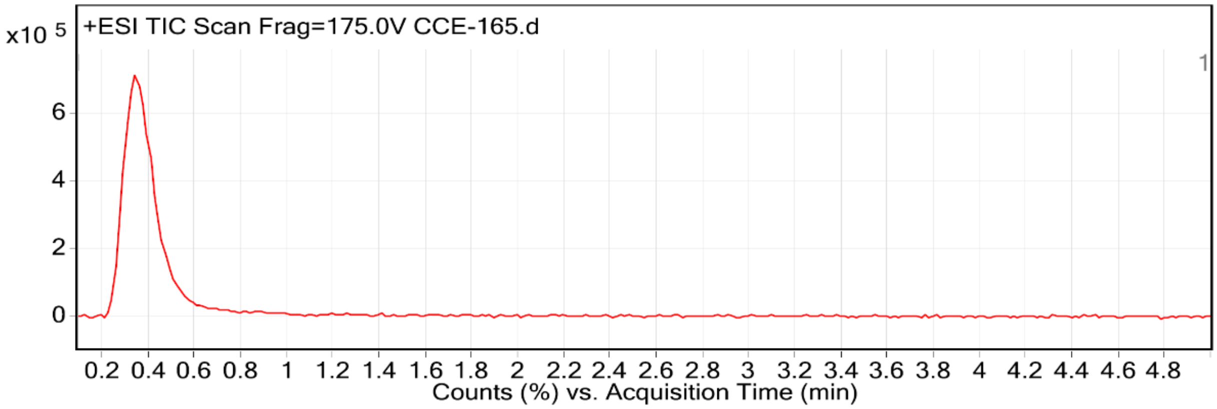

User Spectra

**Fragmentor Voltage**	**Collision Energy**	**Ionization Mode**
175	0	ESI

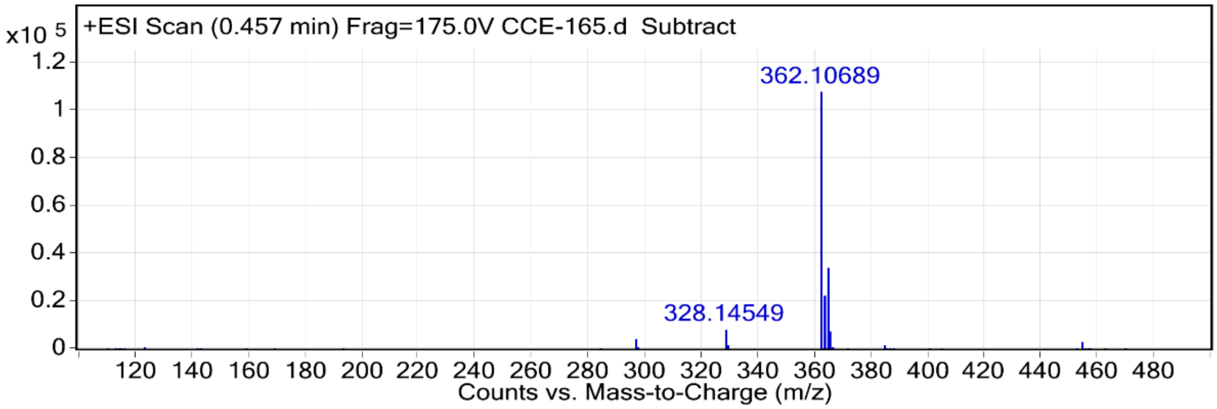

Peak List

m/z	z	Abund	HeightPercent
328.14549		8225.6	7.62
362.10689	1	107960.2	100
363.10913	1	22551.2	20.89
364.10491	1	34471.4	31.93
365.10465	1	7856.2	7.28

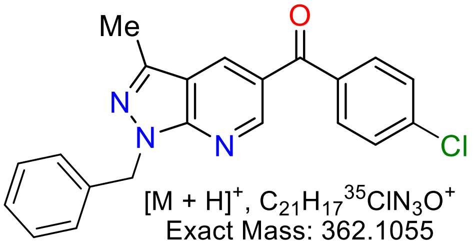

$[M + H]^+$, $C_{21}H_{17}{}^{35}ClN_3O^+$
Exact Mass: 362.1055

Fig. S20 HRMS analysis of (1-benzyl-3-methyl-1*H*-pyrazolo[3,4-*b*]pyridin-5-yl)(4-chlorophenyl)methanone (**3f**)

User Chromatograms

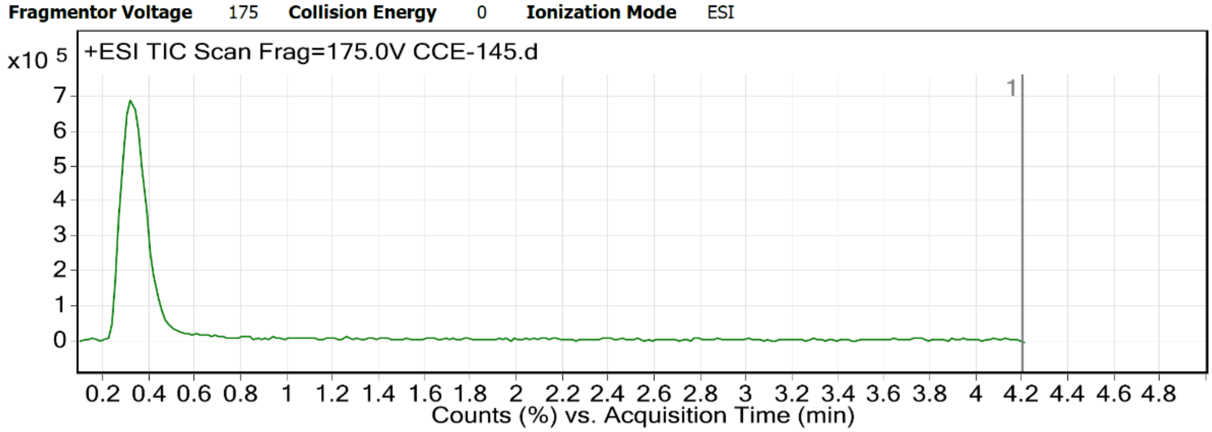

User Spectra

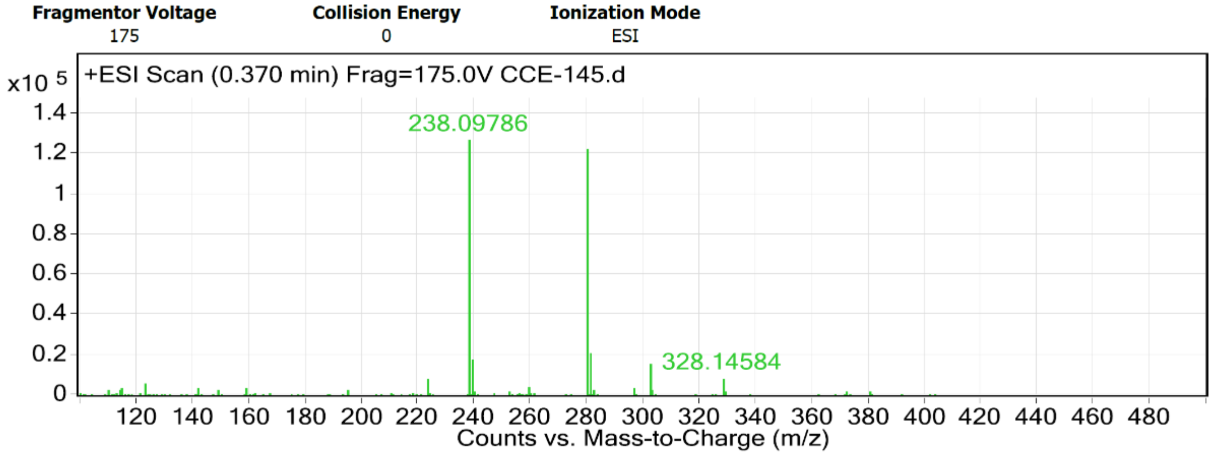

Peak List

m/z	z	Abund	HeightPercent
81.93642		9879.3	7.76
223.07422		8278.6	6.5
238.09786	1	127356.9	100
239.1014	1	18239.9	14.32
280.1081	1	122687.9	96.33
281.1112	1	21154.1	16.61
302.08954		16111.9	12.65
328.14584		8581.7	6.74

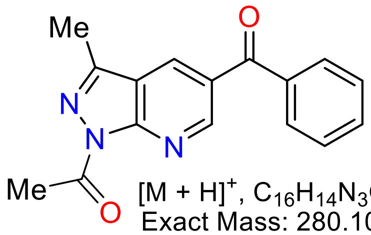

$[M + H]^+$, $C_{16}H_{14}N_3O_2^+$
Exact Mass: 280.1081

Fig. S21 HRMS analysis of 1-(5-benzoyl-3-methyl-1H-pyrazolo[3,4-b]pyridin-1-yl)ethan-1-one (**3g**)

User Chromatograms

Fragmentor Voltage 175 **Collision Energy** 0 **Ionization Mode** ESI

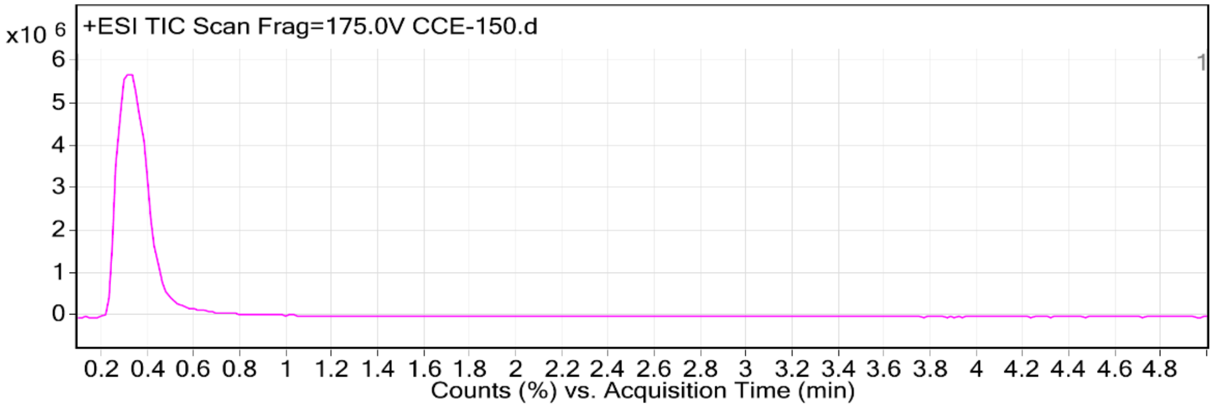

User Spectra

**Fragmentor Voltage**	**Collision Energy**	**Ionization Mode**
175	0	ESI

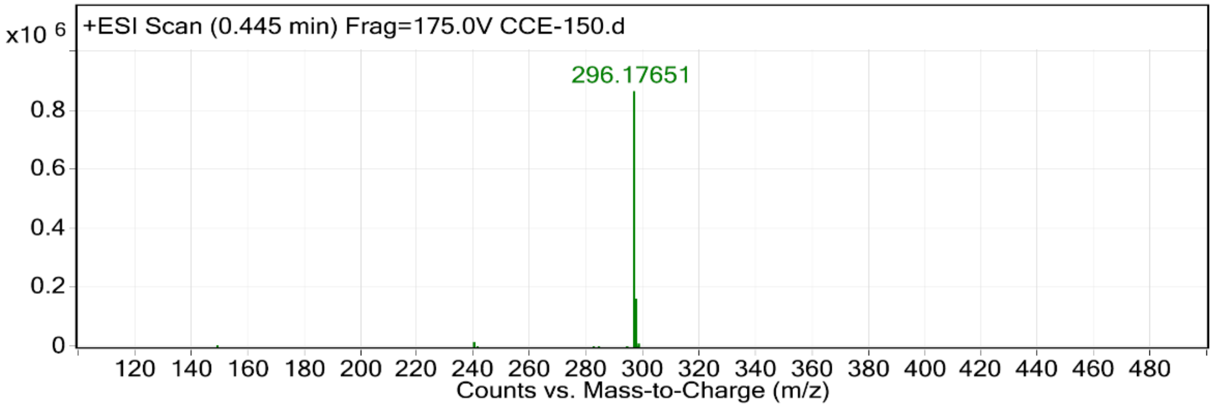

Peak List

m/z	z	Abund	HeightPercent
296.17651	1	867763.8	100
297.18054	1	163729.1	18.87

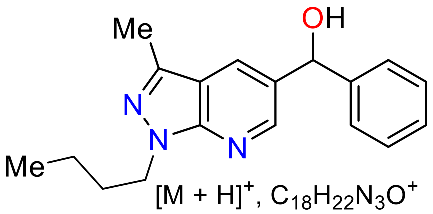

$[M + H]^+$, $C_{18}H_{22}N_3O^+$
Exact Mass: 296.1757

Fig. S22 HRMS analysis of (1-butyl-3-methyl-1*H*-pyrazolo[3,4-*b*]pyridin-5-yl)(phenyl)methanol (**4a**)

User Chromatograms

Fragmentor Voltage 175 **Collision Energy** 0 **Ionization Mode** ESI

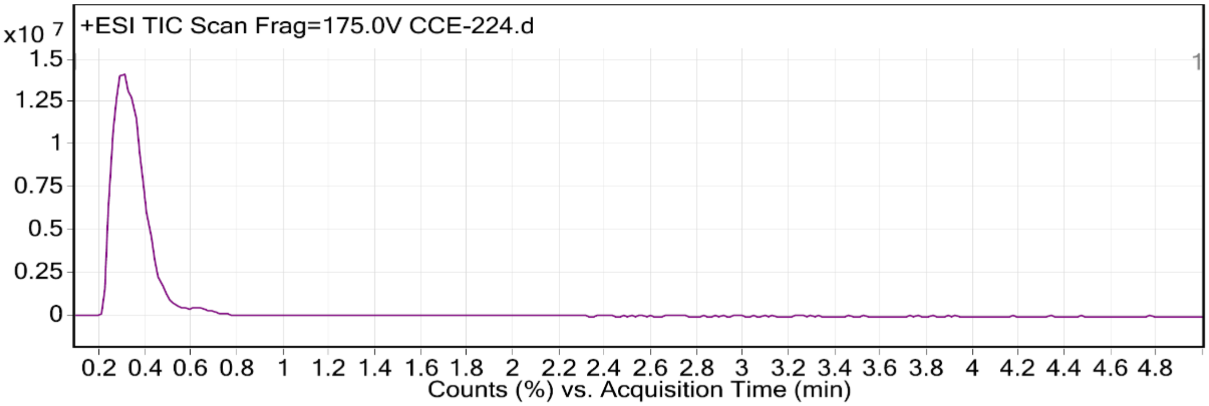

User Spectra

Fragmentor Voltage	Collision Energy	Ionization Mode
175	0	ESI

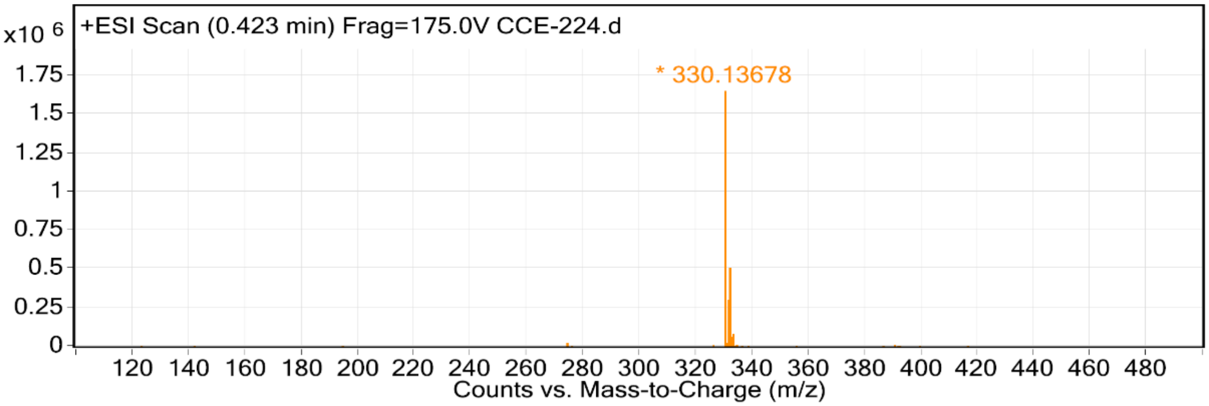

Peak List

m/z	z	Abund	HeightPercent
330.13678	1	1654864.1	100
330.18206		127058.5	7.68
330.22362		89965.9	5.44
331.13971	1	304508.8	18.4
332.13398	1	510502.1	30.85
333.13725	1	92534.3	5.59

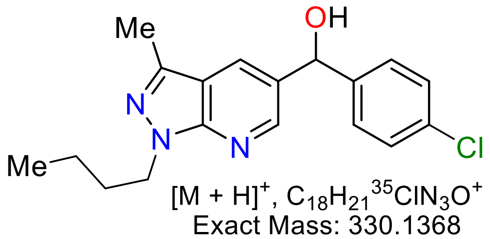

$[M + H]^+$, $C_{18}H_{21}{}^{35}ClN_3O^+$
Exact Mass: 330.1368

Fig. S23 HRMS analysis of (1-butyl-3-methyl-1*H*-pyrazolo[3,4-*b*]pyridin-5-yl)(4-chlorophenyl)methanol (**4b**)

5. Copies of NMR spectra

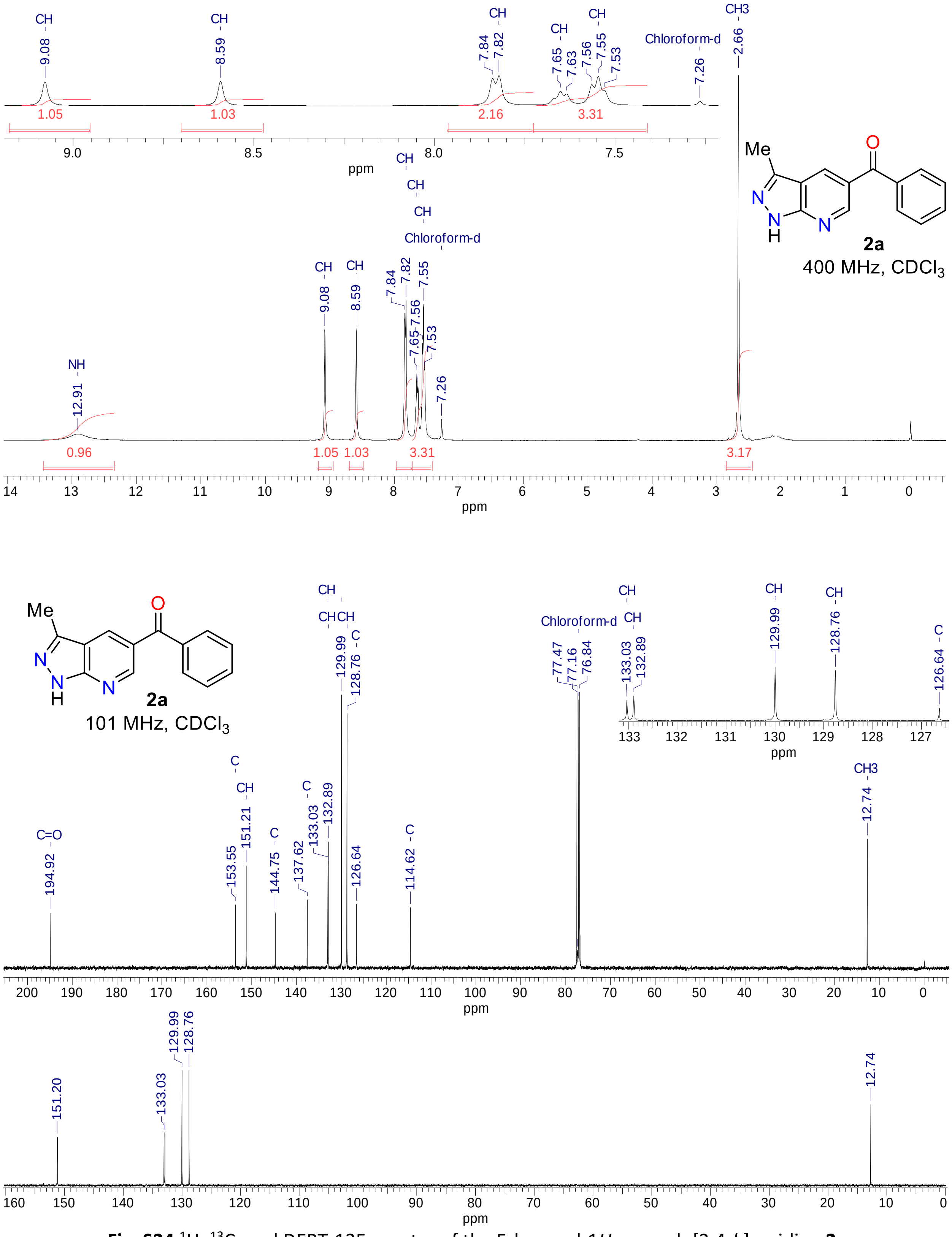

Fig. S24 ^{1}H, ^{13}C, and DEPT-135 spectra of the 5-benzoyl-1*H*-pyrazolo[3,4-*b*]pyridine **2a**

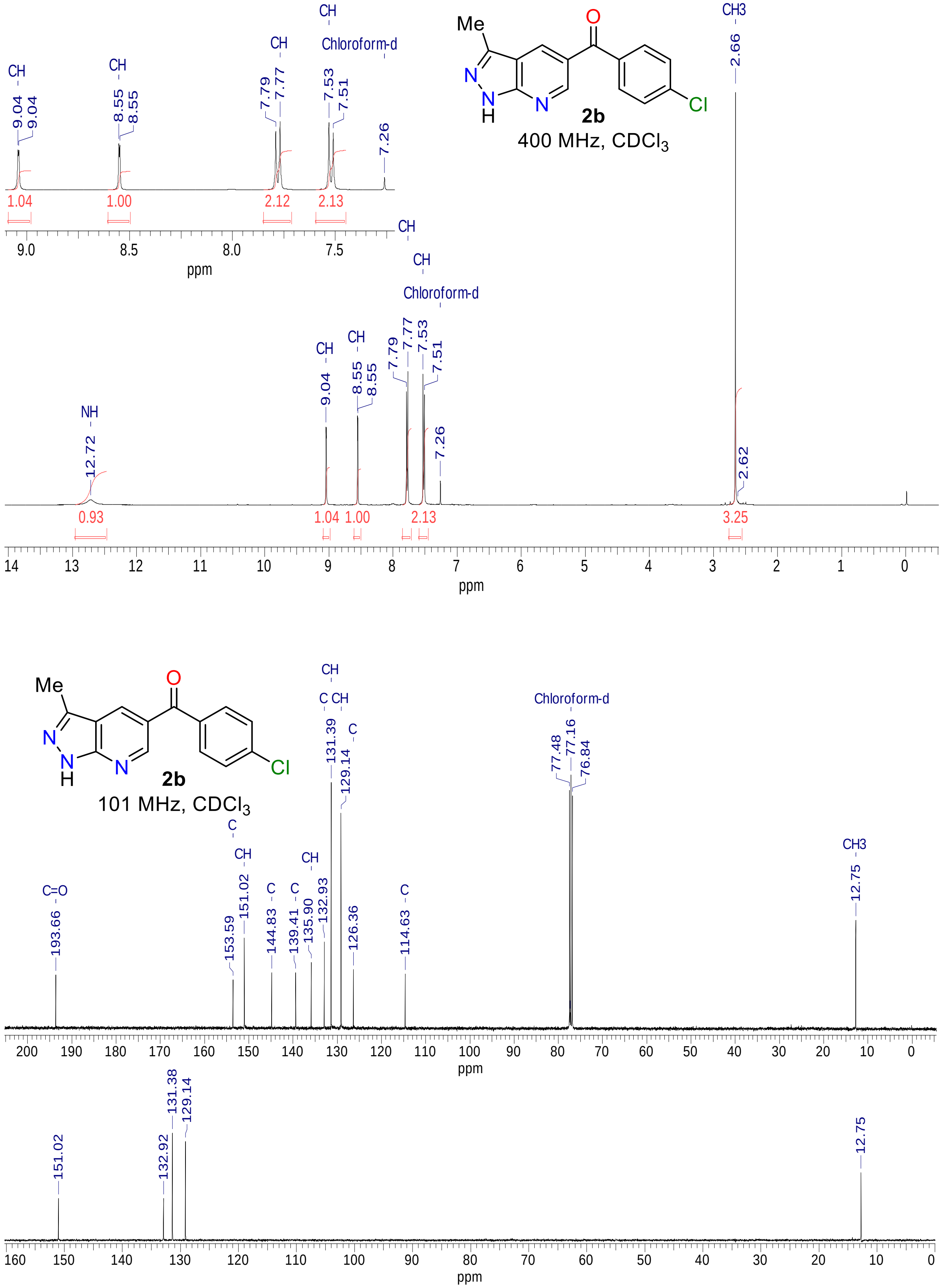

Fig. S25 ^{1}H, ^{13}C, and DEPT-135 spectra of the 5-(4-chlorobenzoyl)-1*H*-pyrazolo[3,4-*b*]pyridine **2b**

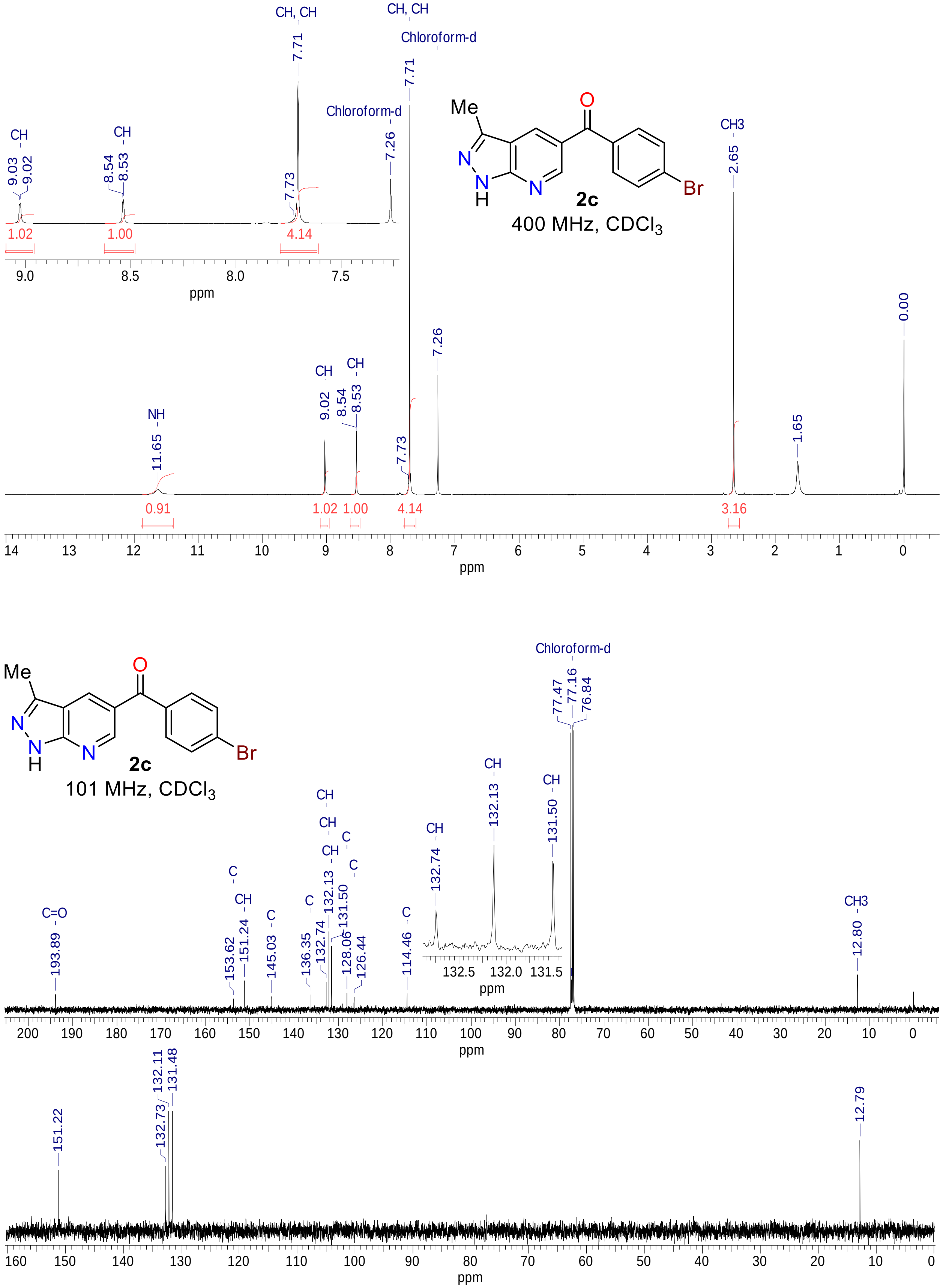

Fig. S26 ^{1}H, ^{13}C, and DEPT-135 spectra of the 5-(4-bromobenzoyl)-1*H*-pyrazolo[3,4-*b*]pyridine **2c**

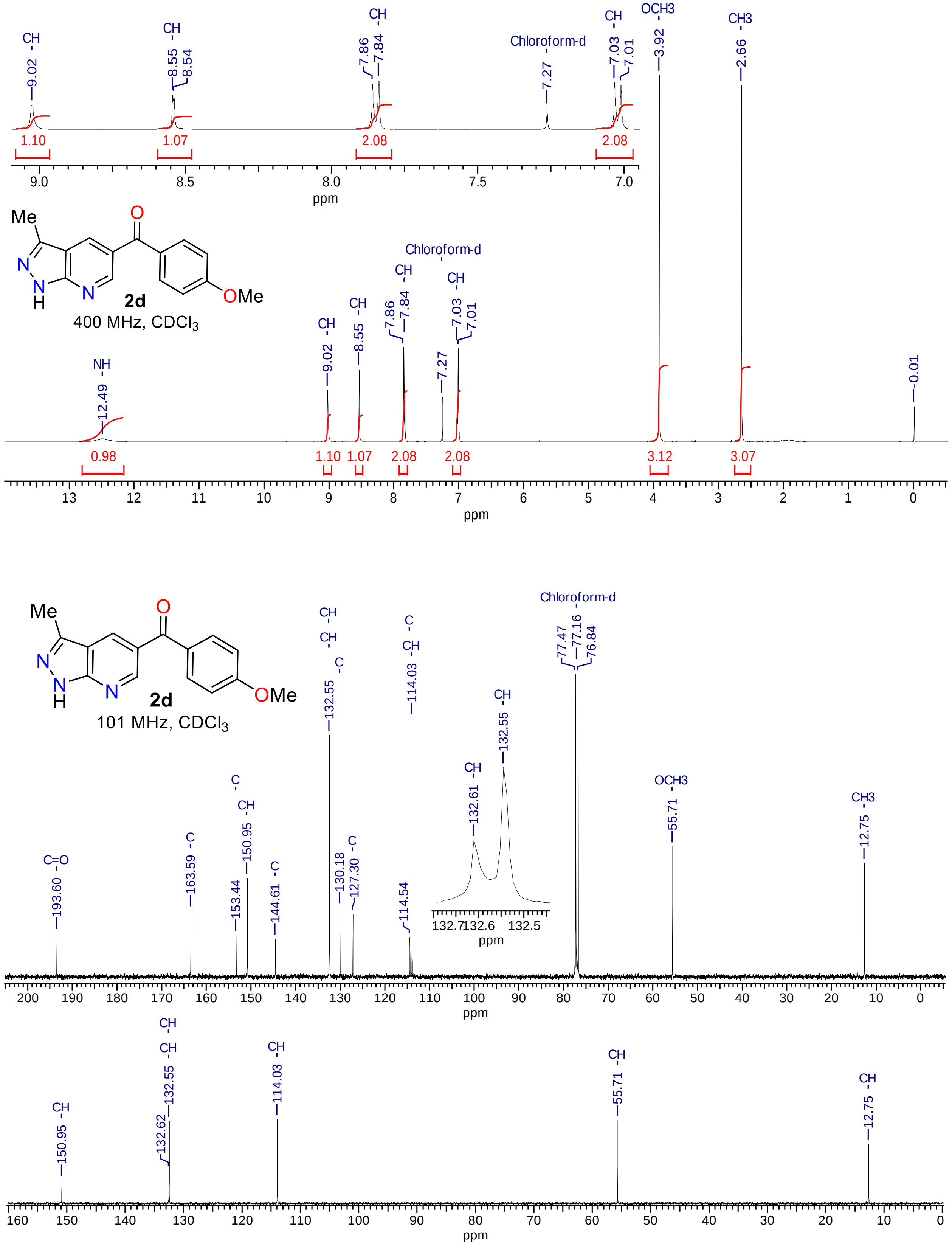

Fig. S27 ^{1}H, ^{13}C, and DEPT-135 spectra of the 5-(4-methoxybenzoyl)-1*H*-pyrazolo[3,4-*b*]pyridine **2d**

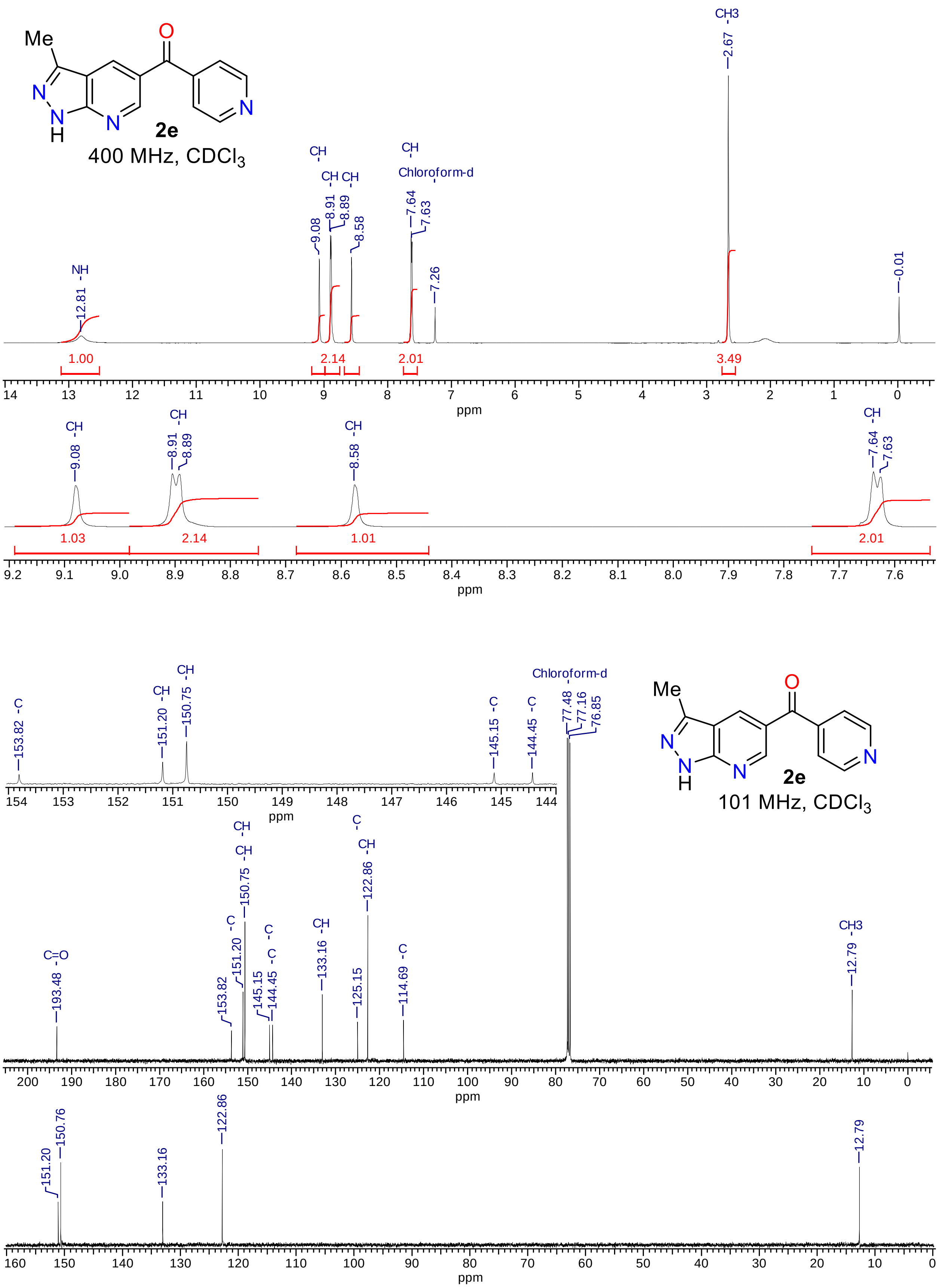

Fig. S28 ^{1}H, ^{13}C, and DEPT-135 spectra of the 5-(4-pyridinoyl)-1*H*-pyrazolo[3,4-*b*]pyridine **2e**

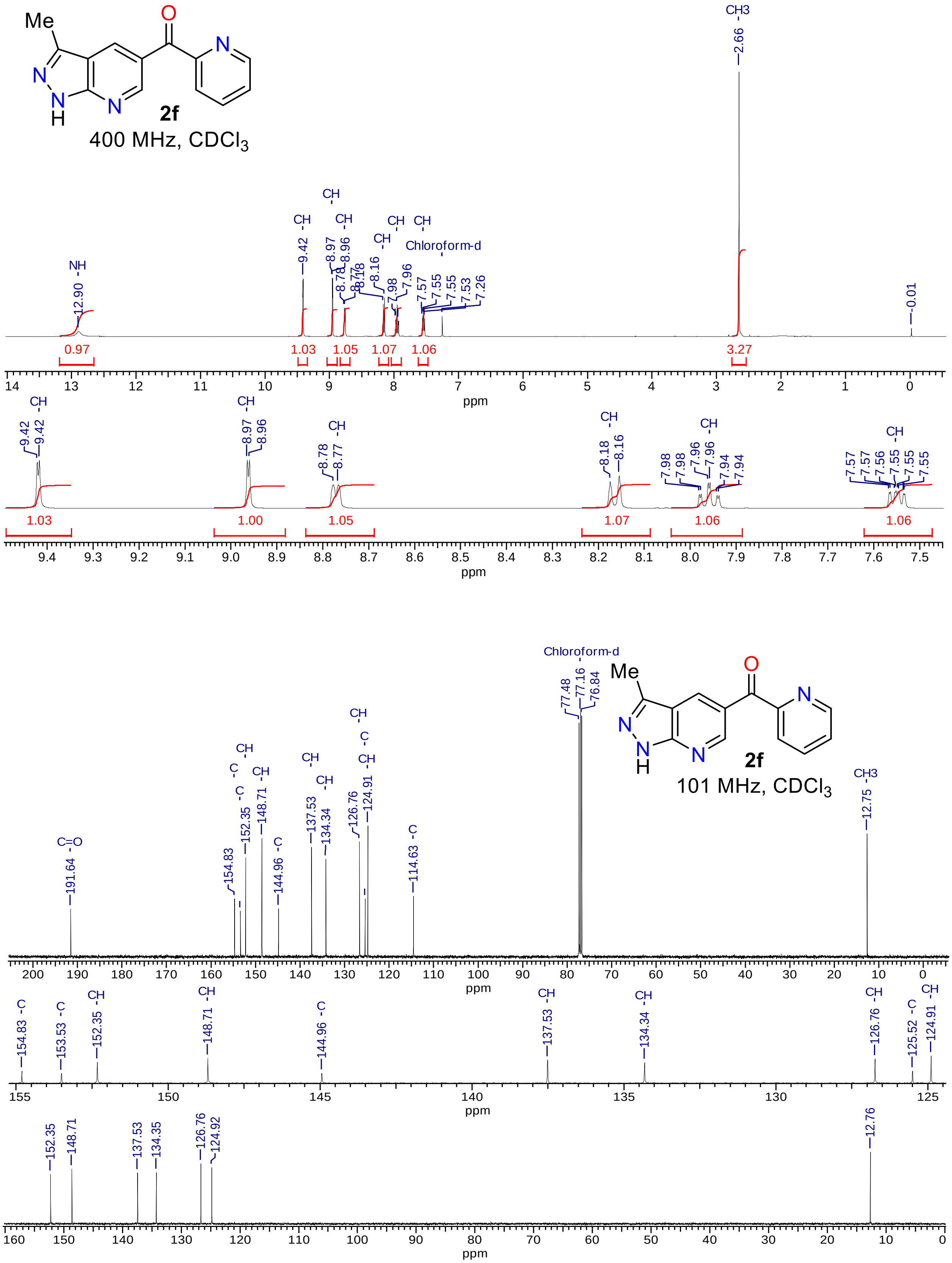

Fig. S29. ^{1}H, ^{13}C, and DEPT-135 spectra of the 5-(2-pyridinoyl)-1*H*-pyrazolo[3,4-*b*]pyridine **2f**

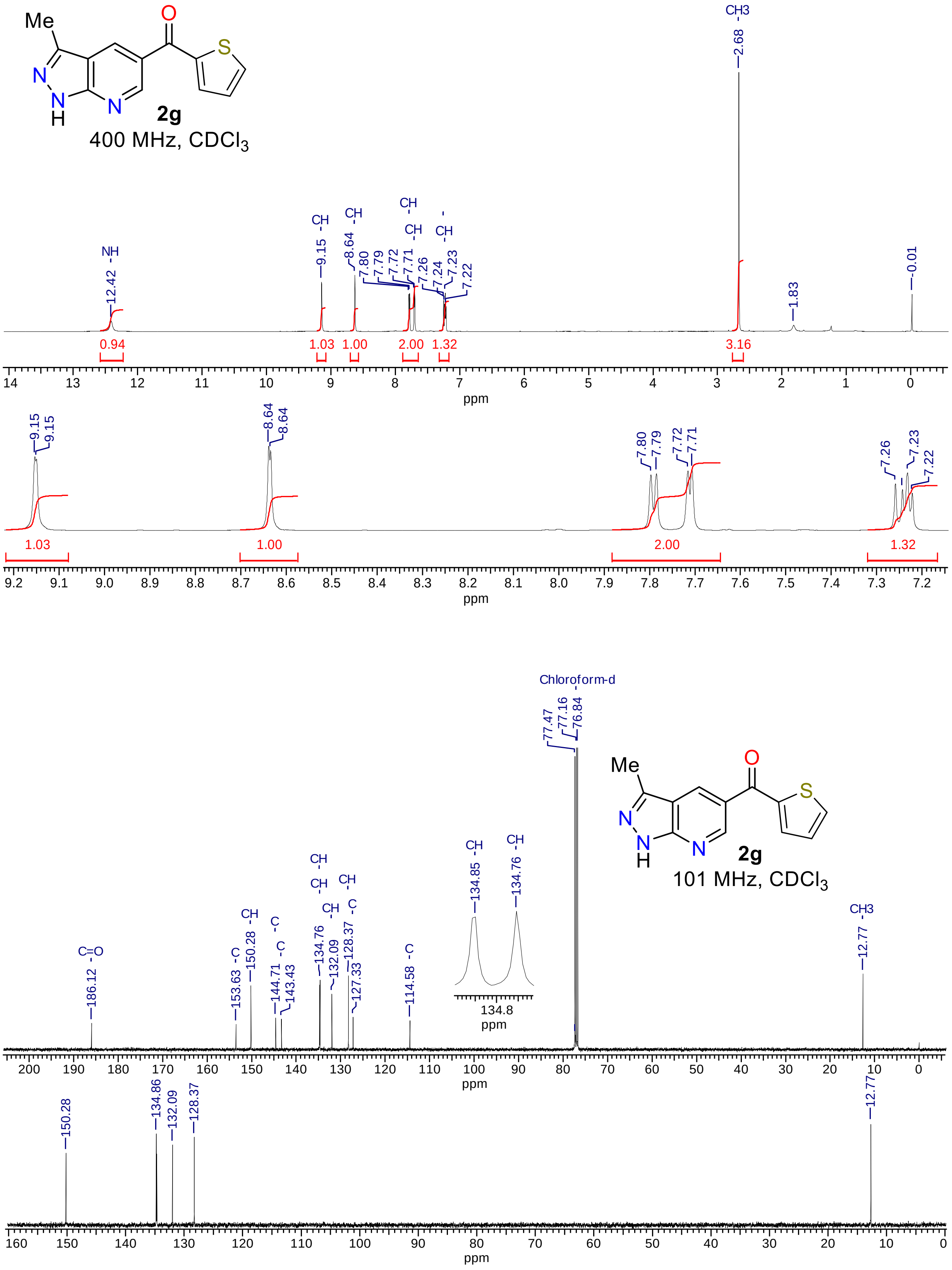

Fig. S30 ^{1}H, ^{13}C, and DEPT-135 spectra of the 5-(2-thienoyl)-1*H*-pyrazolo[3,4-*b*]pyridine **2g**

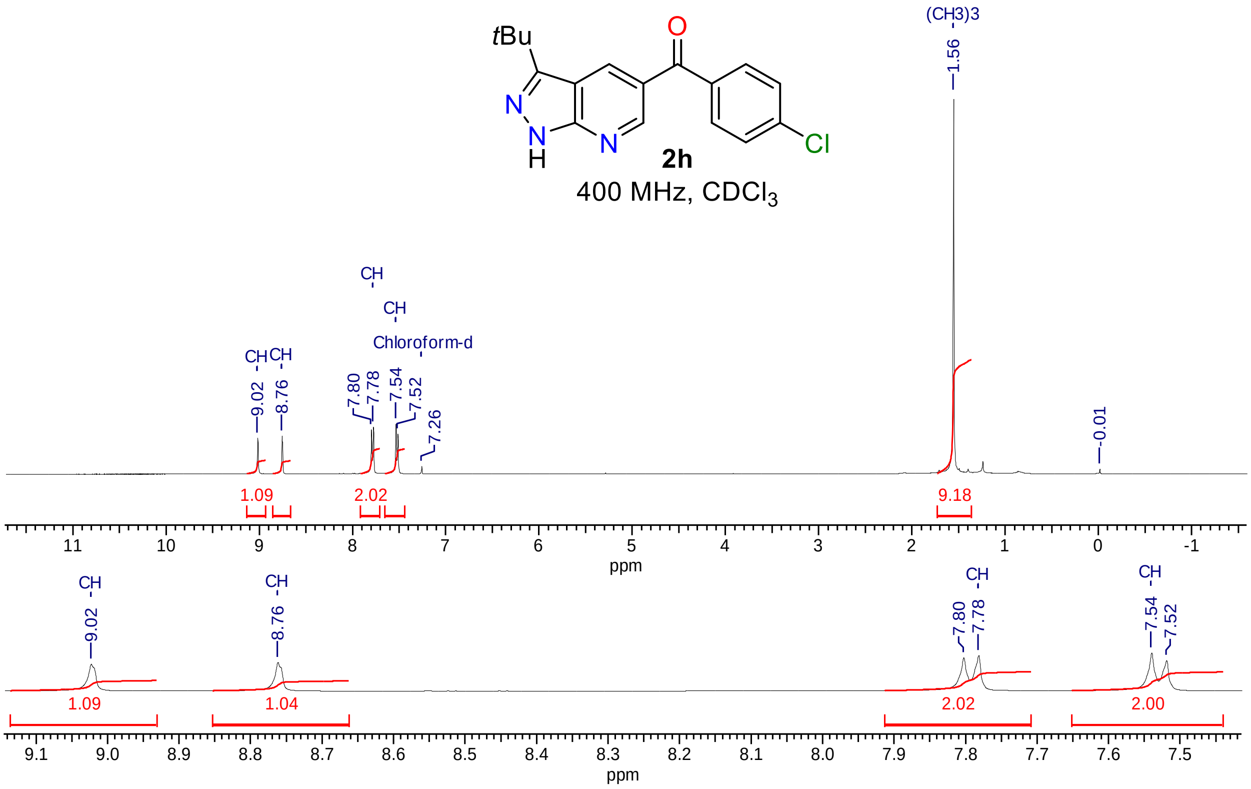

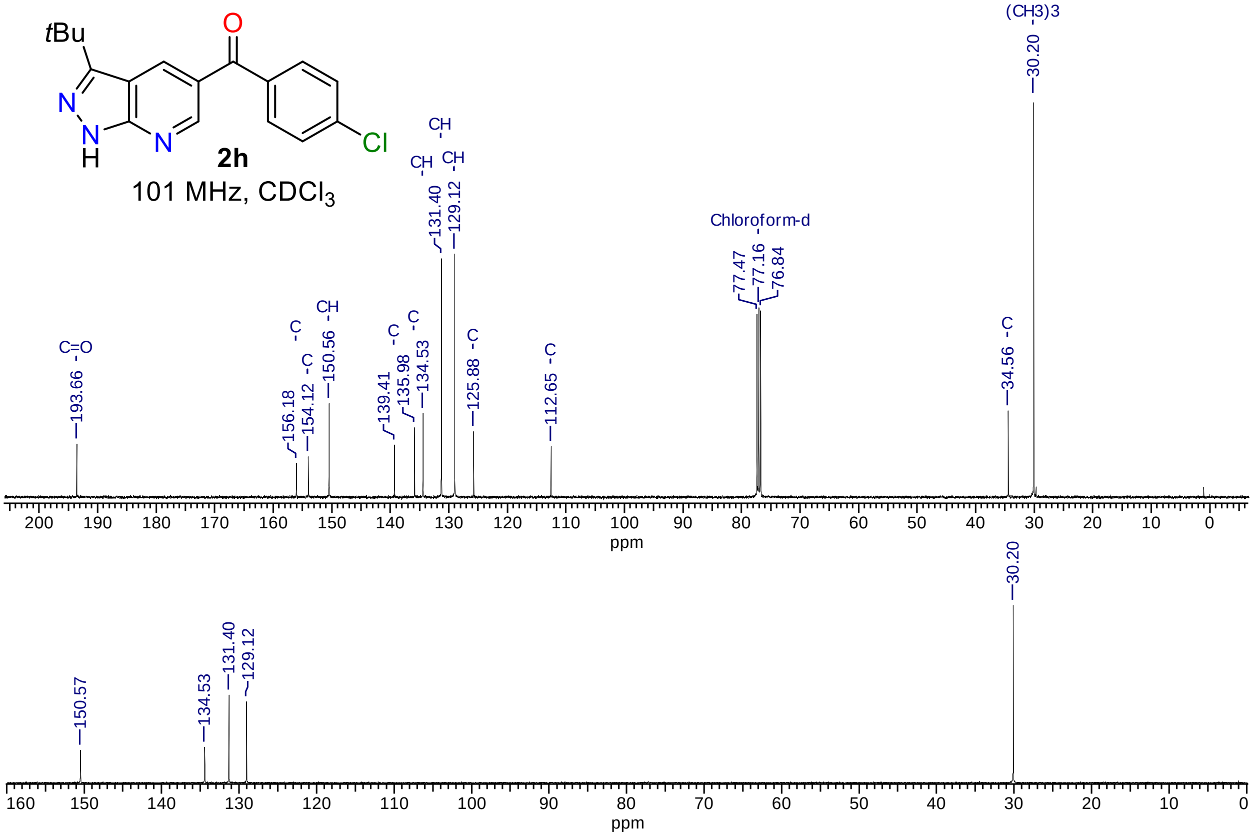

Fig. S31 ^{1}H, ^{13}C, and DEPT-135 spectra of the 5-(4-chlorobenzoyl)-1*H*-pyrazolo[3,4-*b*]pyridine **2h**

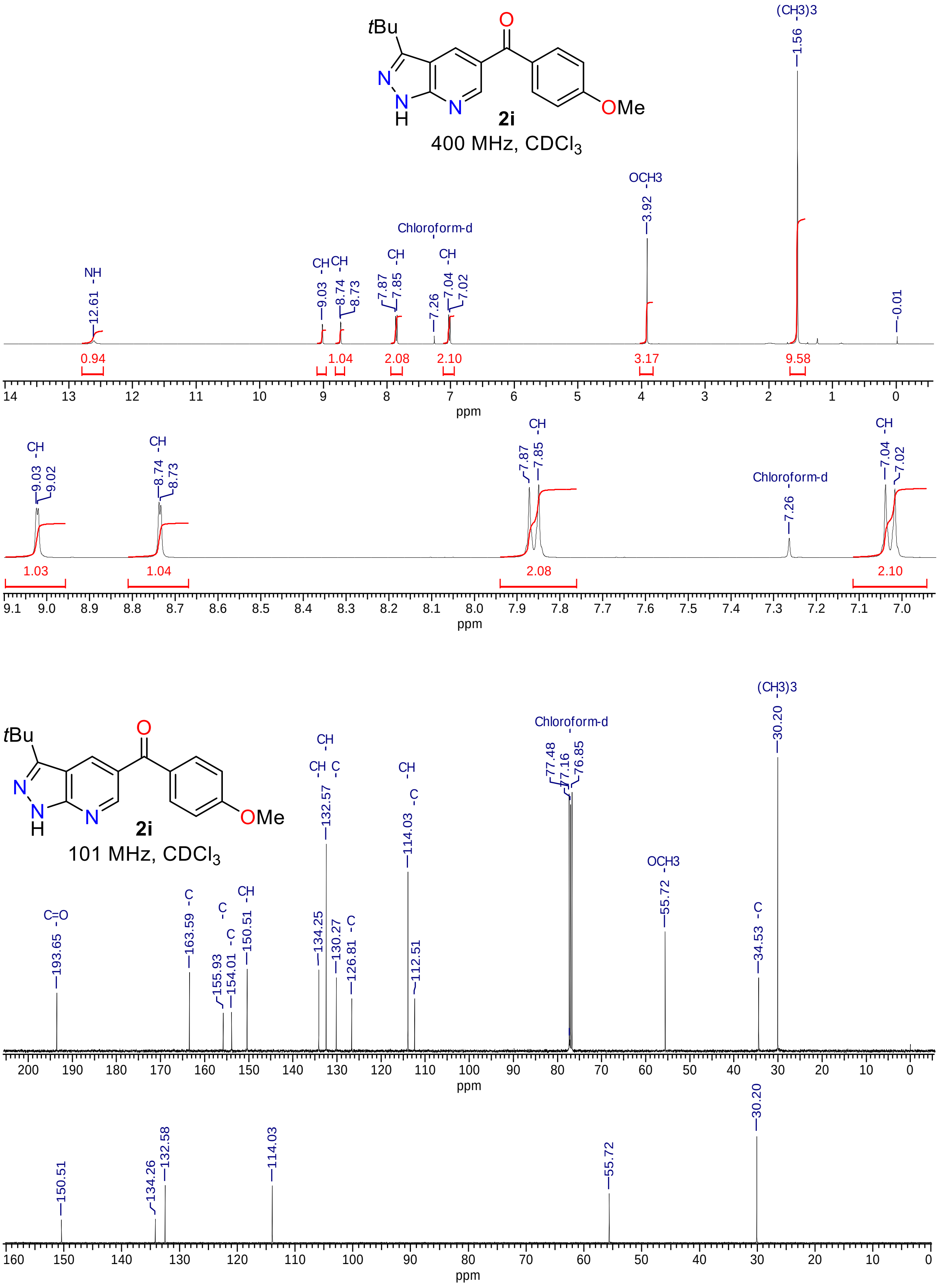

Fig. S32 ^{1}H, ^{13}C, and DEPT-135 spectra of the 5-(4-methoxybenzoyl)-1*H*-pyrazolo[3,4-*b*]pyridine **2i**

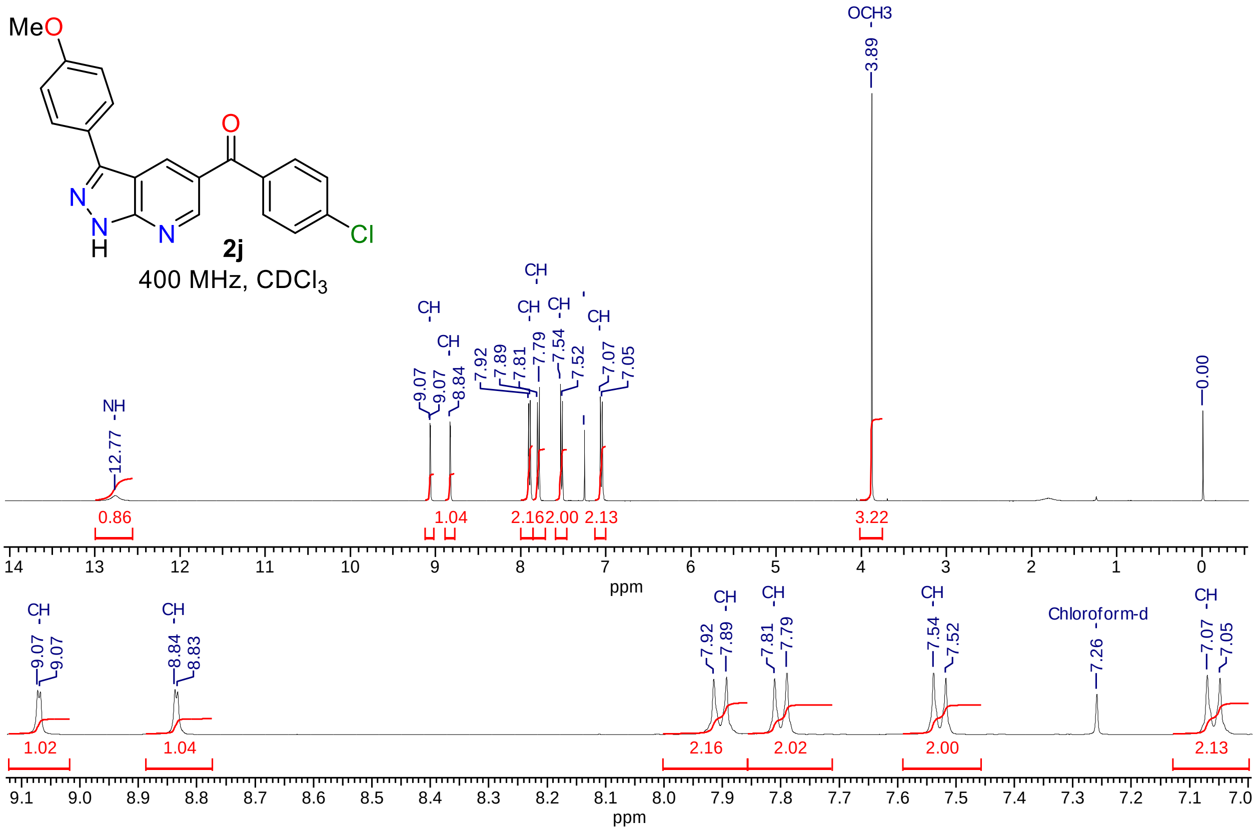

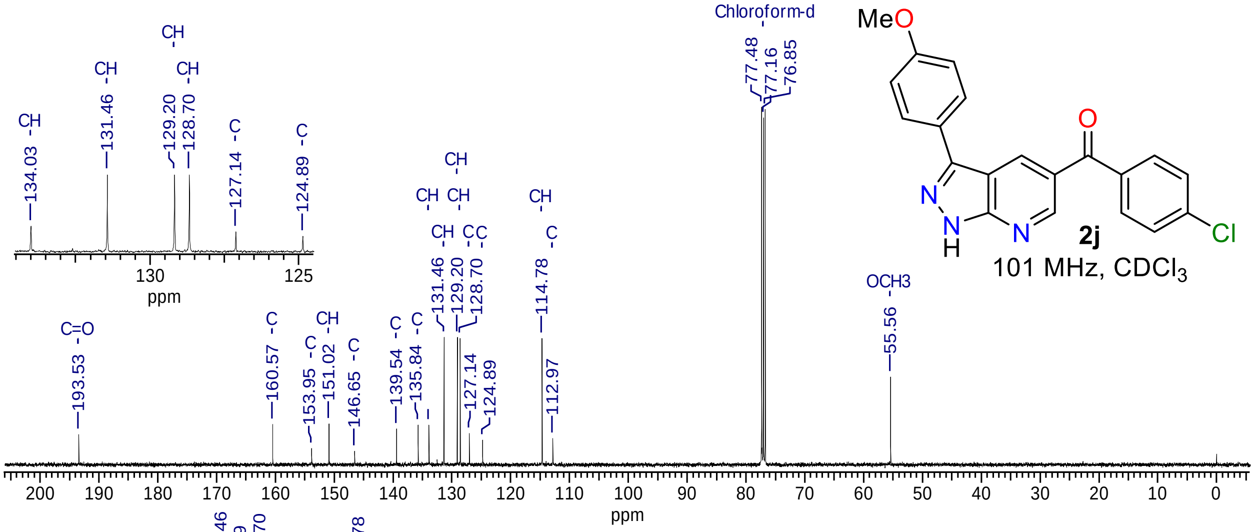

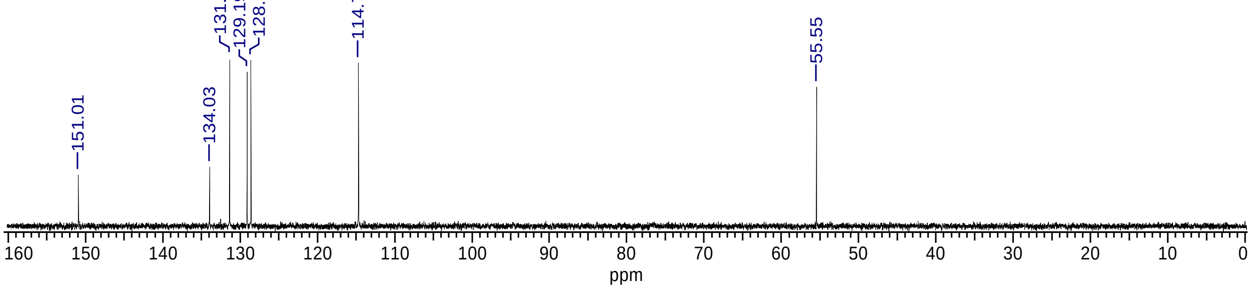

Fig. S33 ^{1}H, ^{13}C, and DEPT-135 spectra of the 3-aryl-5-(4-chlorobenzoyl)-1*H*-pyrazolo[3,4-*b*]pyridine **2j**

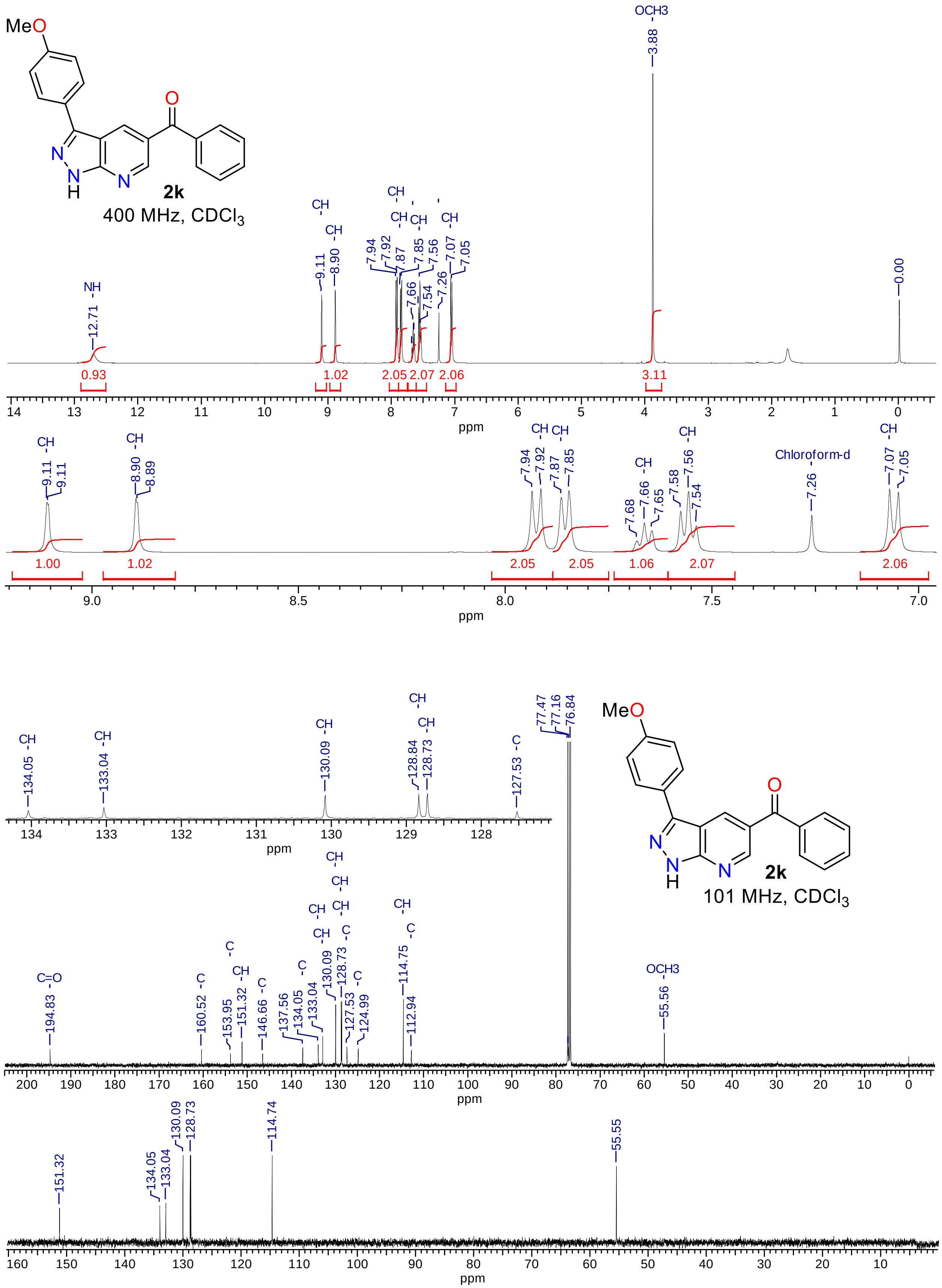

Fig. S34 ^{1}H, ^{13}C, and DEPT-135 spectra of the 3-aryl-5-benzoyl-1*H*-pyrazolo[3,4-*b*]pyridine **2k**

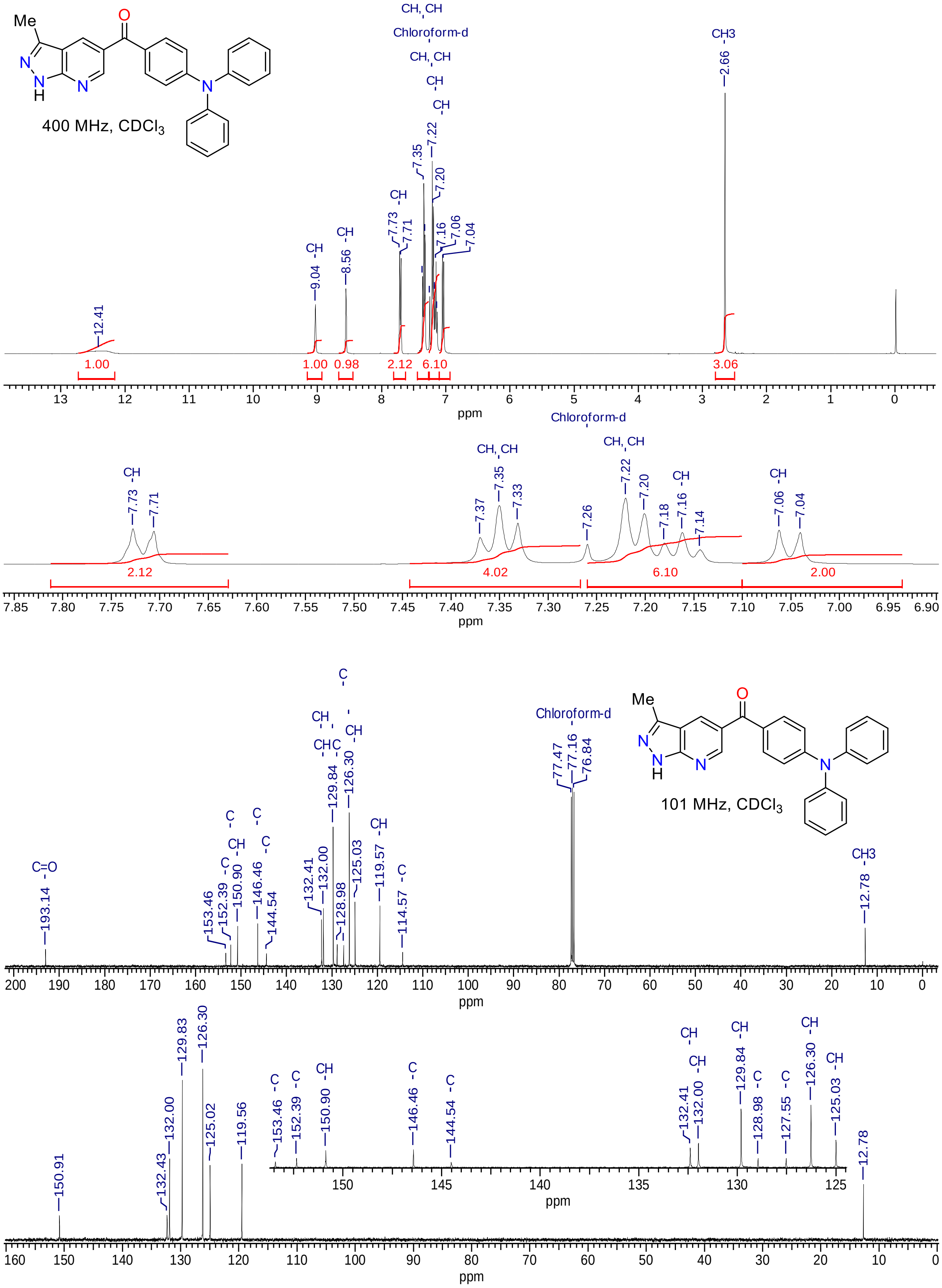

Fig. S35 ^{1}H, ^{13}C, and DEPT-135 spectra of the 5-(4-difenilaminobenzoyl)-1*H*-pyrazolo[3,4-*b*]pyridine **2l**

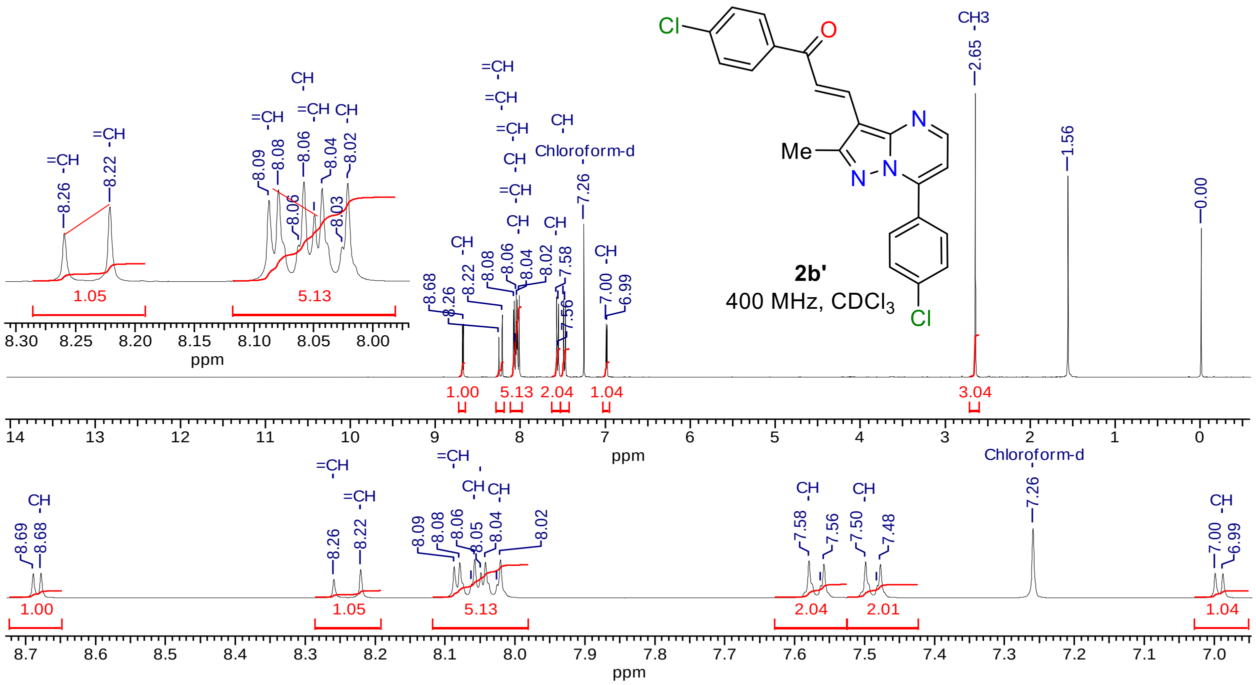

Fig. S36 ^{1}H spectra of the chalcone derivative **2'**

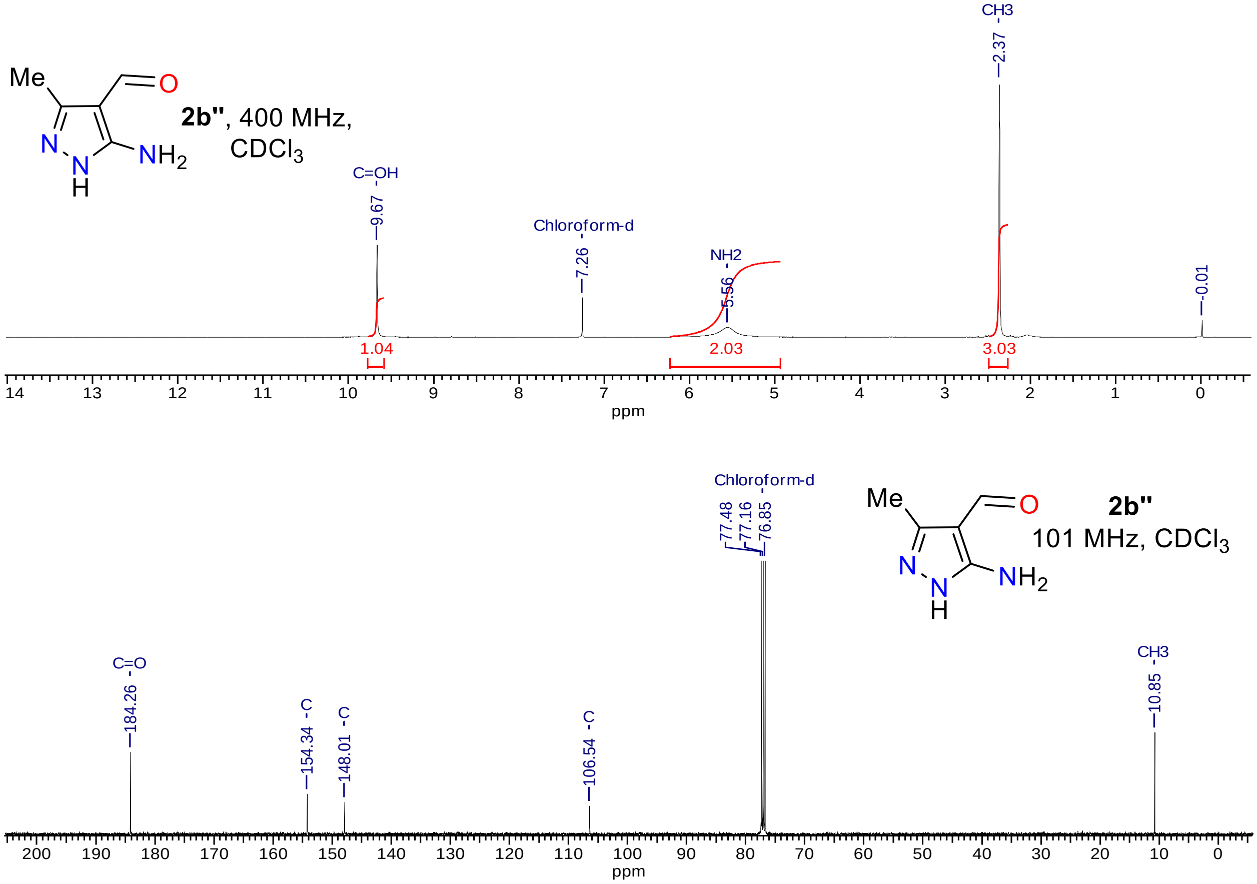

Fig. S37 ^{1}H, ^{13}C{^{1}H}, and DEPT-135 of 5-amino-3-methyl-1H-pyrazole-4-carbaldehyde (**2''**)

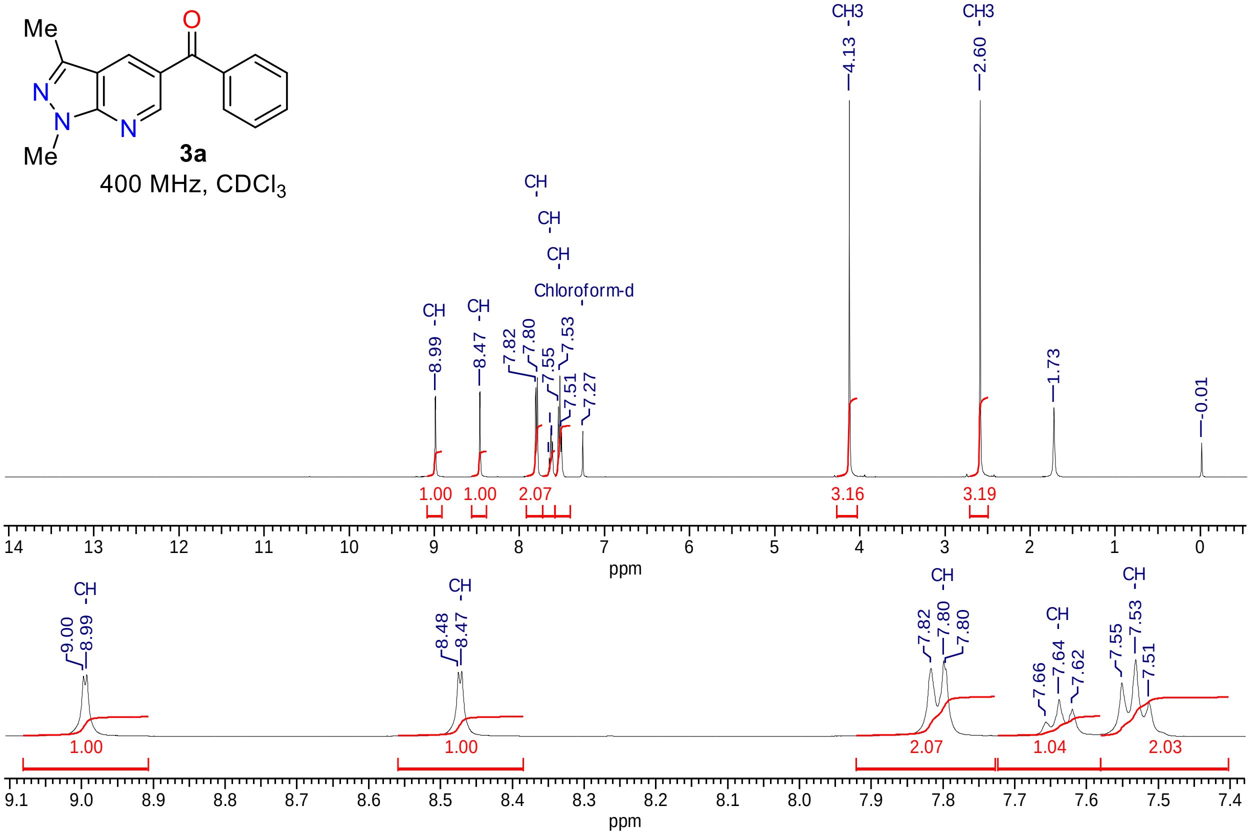

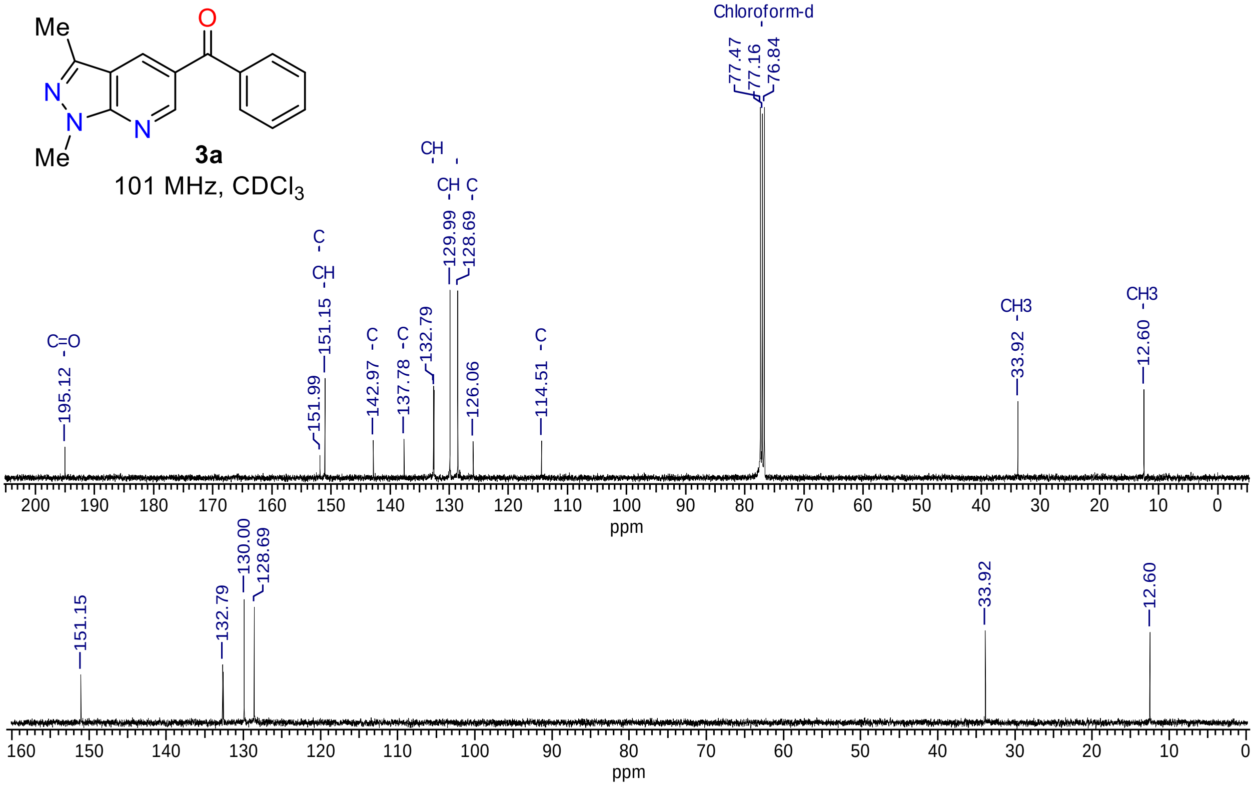

Fig. S38 ^{1}H, ^{13}C, and DEPT-135 spectra of 5-benzoyl-*N*-methyl derivative **3a**

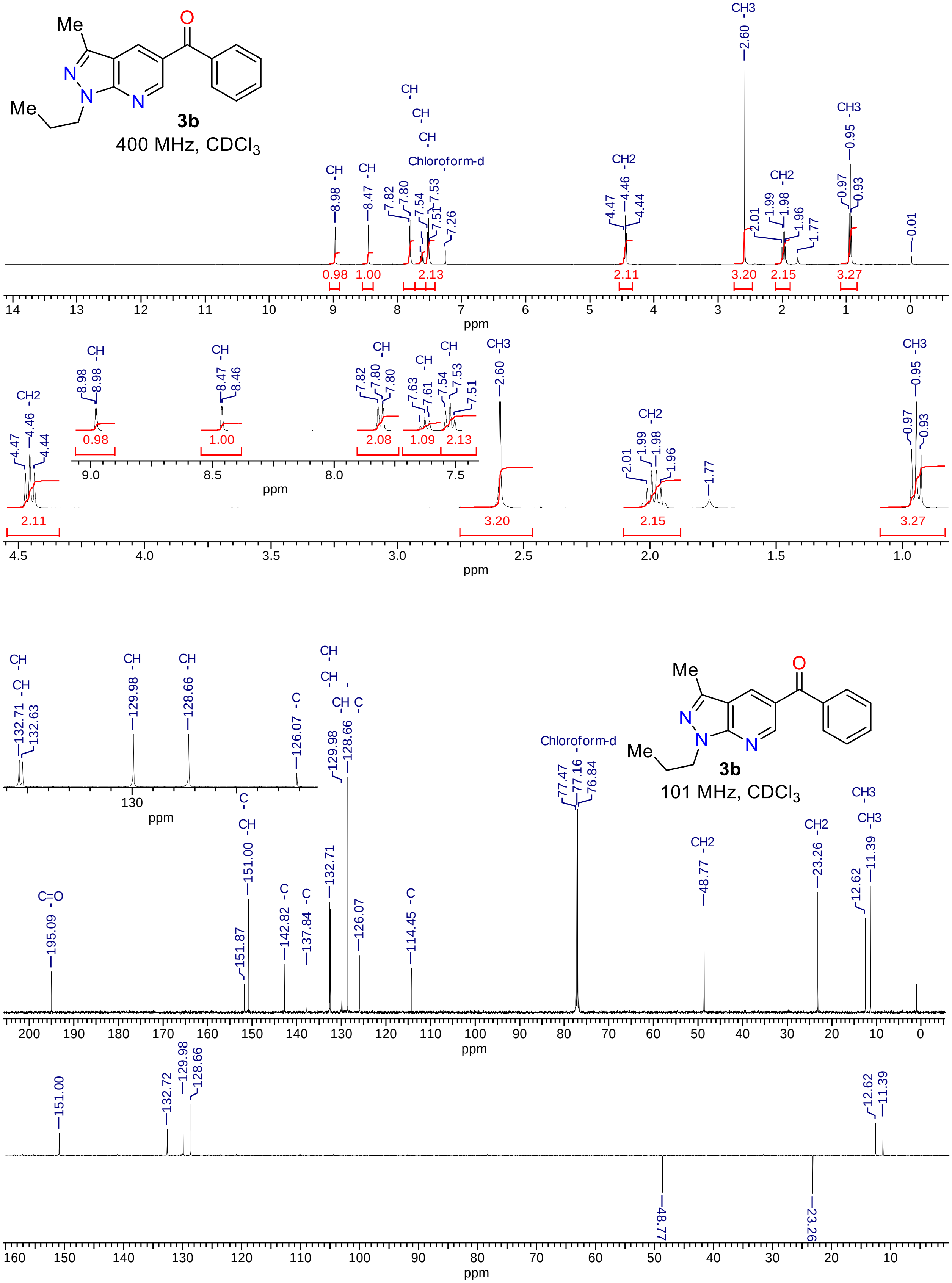

Fig. S39 ^{1}H, ^{13}C, and DEPT-135 spectra of 5-benzoyl-*N*-propyl derivative **3b**

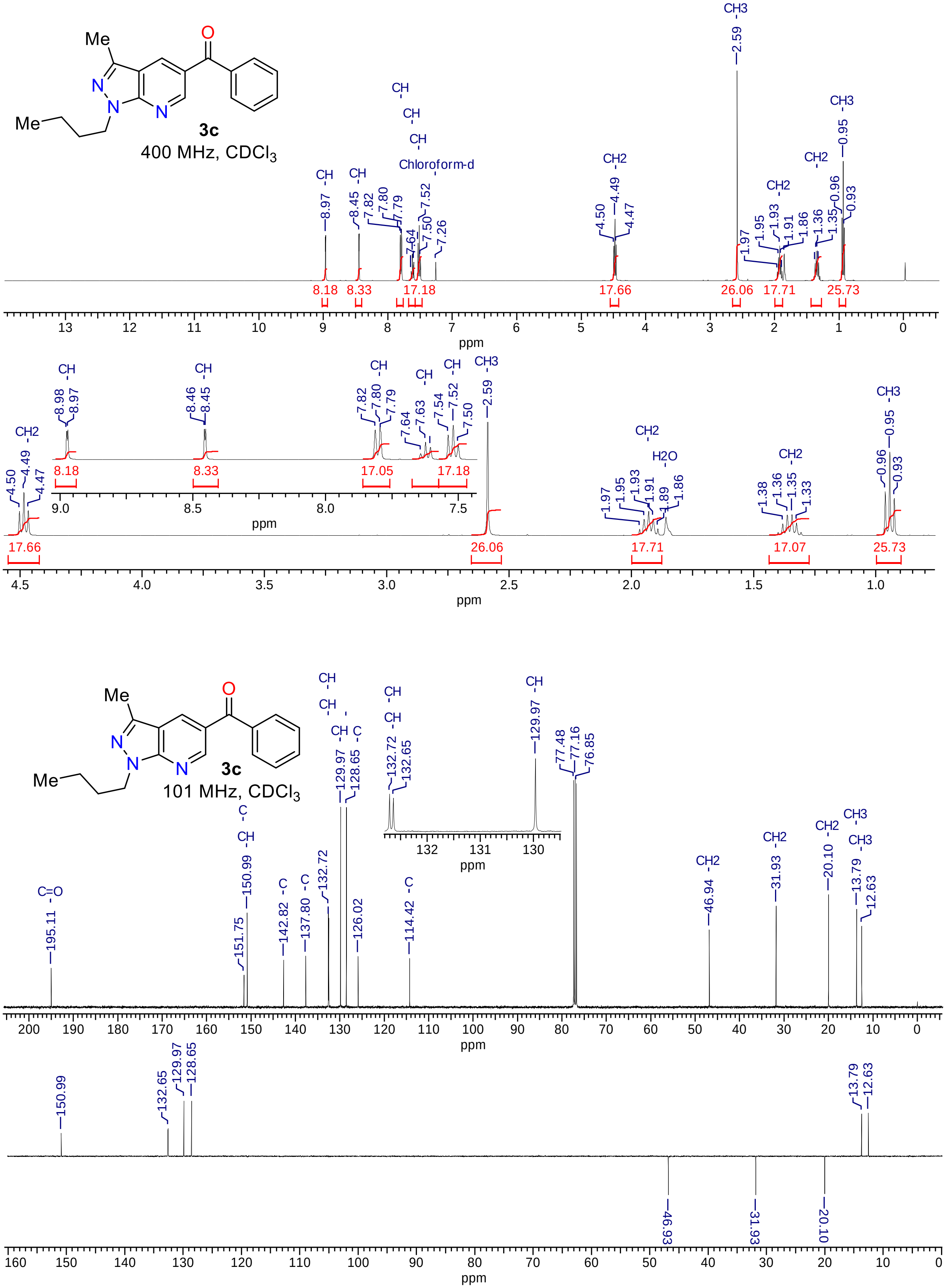

Fig. S40 ^{1}H, ^{13}C, and DEPT-135 spectra of 5-benzoyl-*N*-butyl derivative **3c**

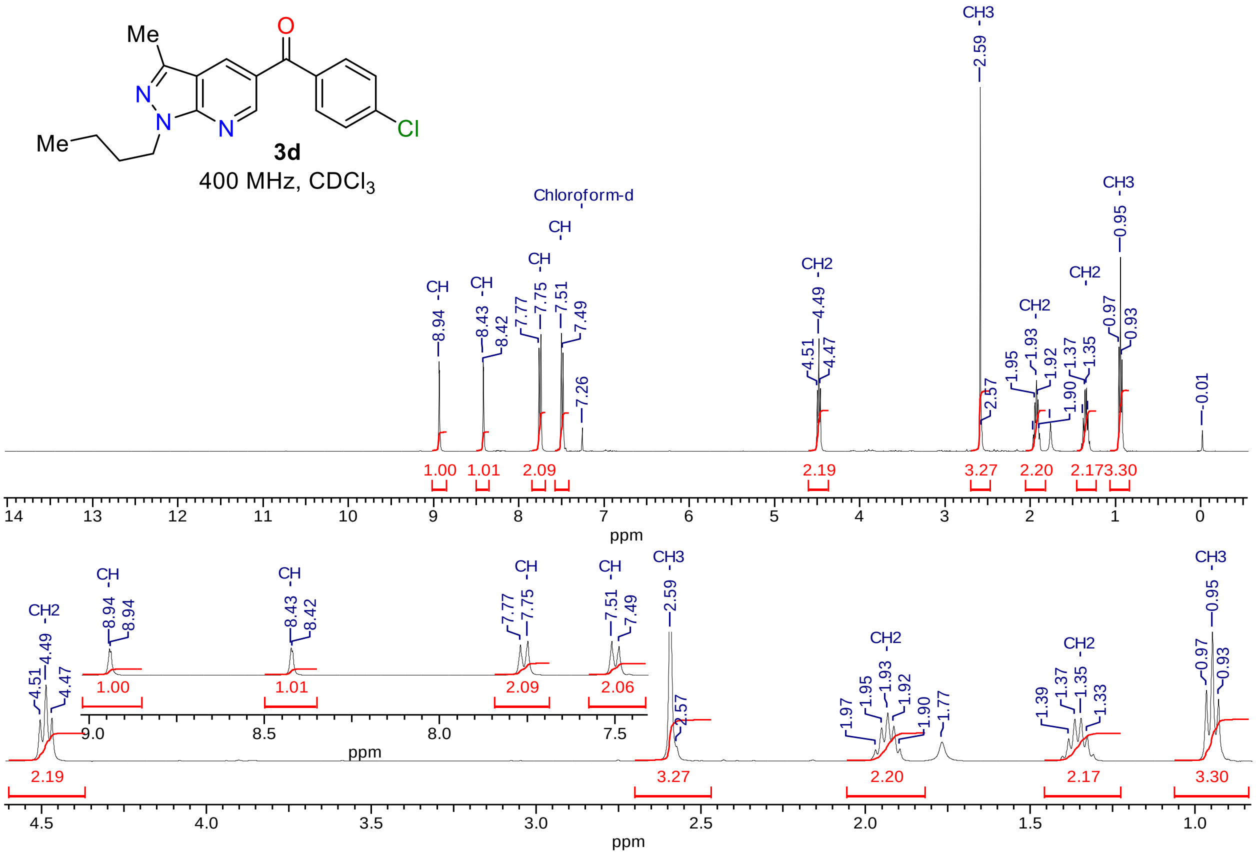

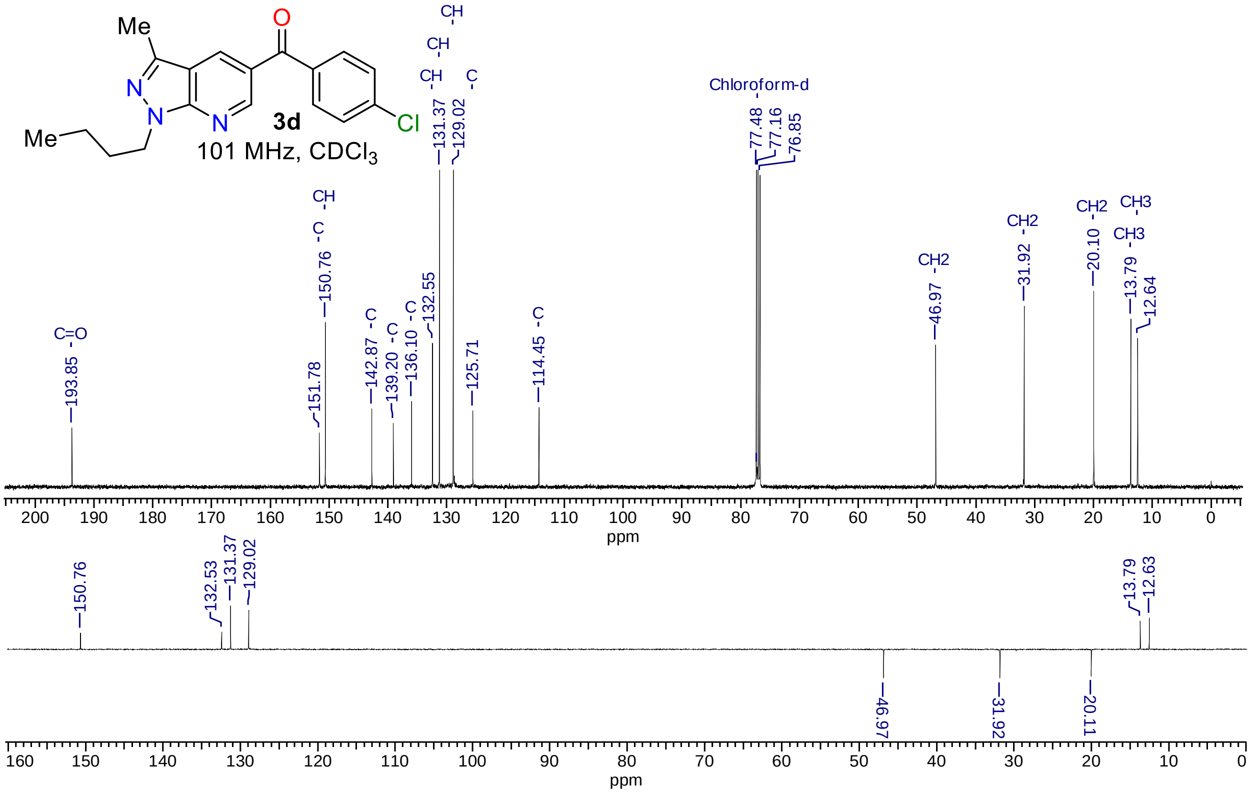

Fig. S41 ^{1}H, ^{13}C, and DEPT-135 spectra of 5-(4-chlorobenzoyl)-*N*-butyl derivative **3d**

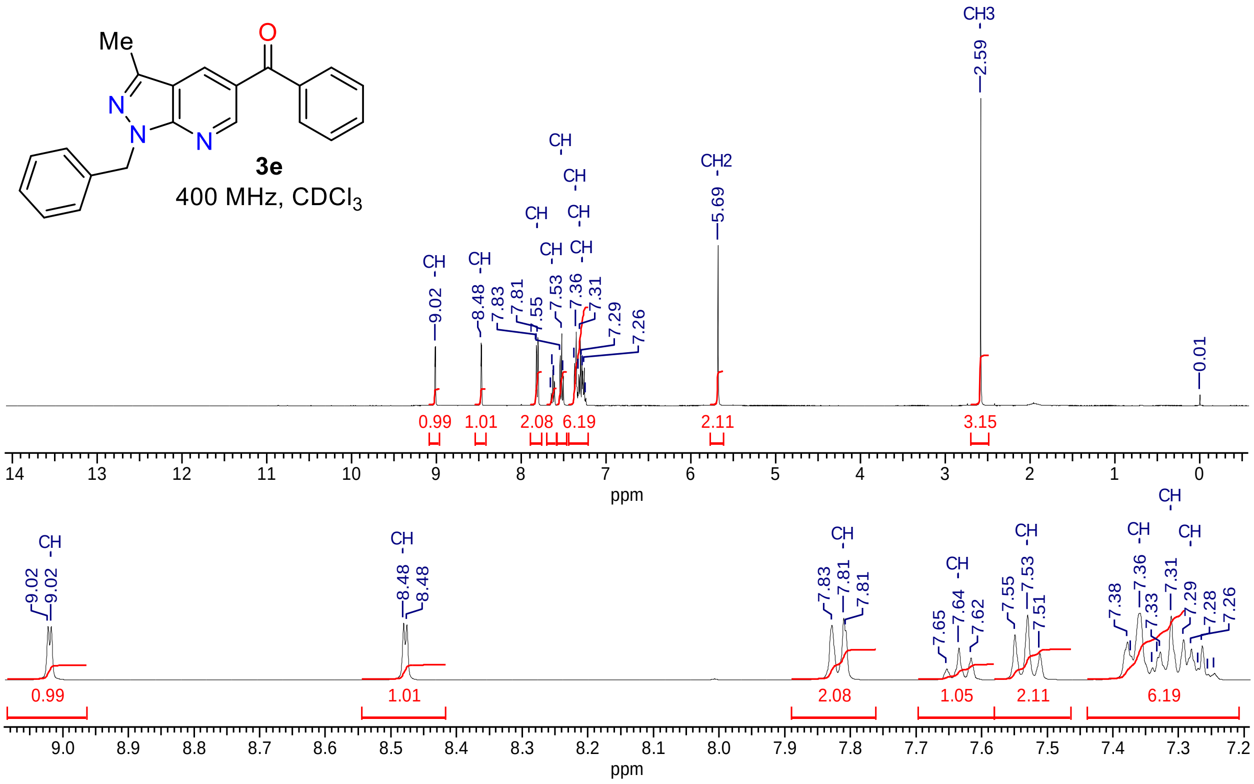

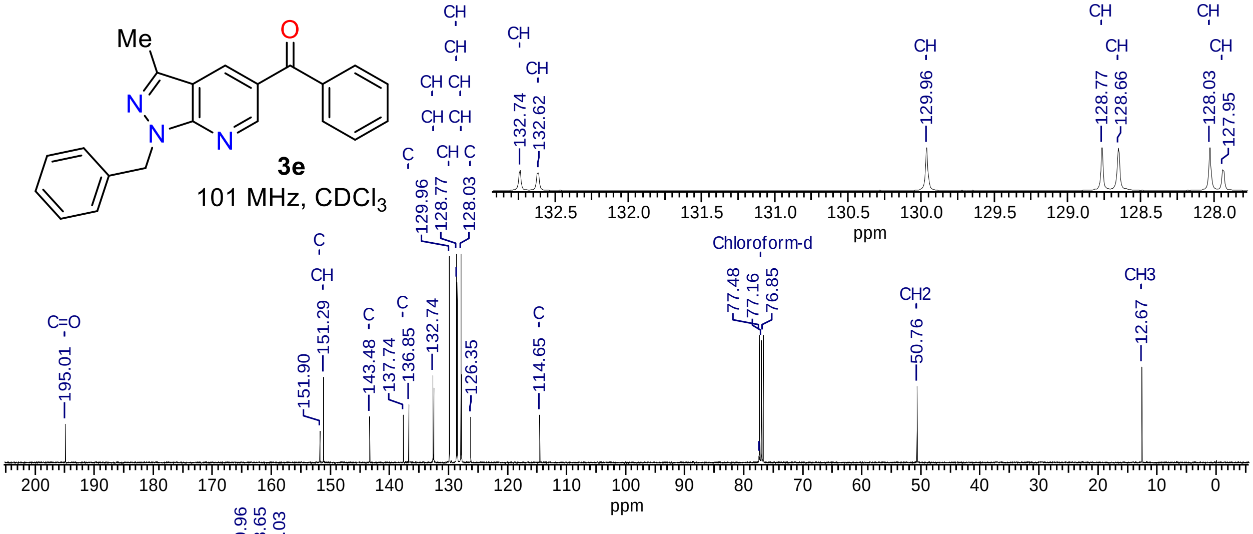

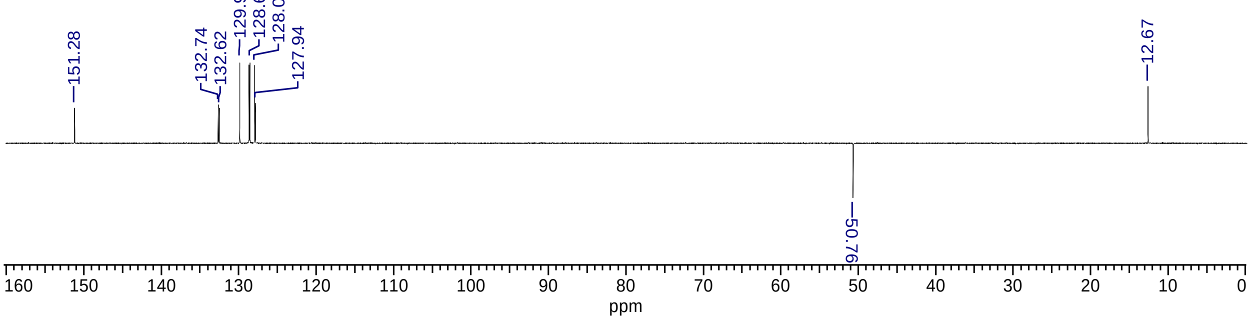

Fig. S42 ^{1}H, ^{13}C, and DEPT-135 spectra of 5-benzoyl-*N*-benzyl derivative **3e**

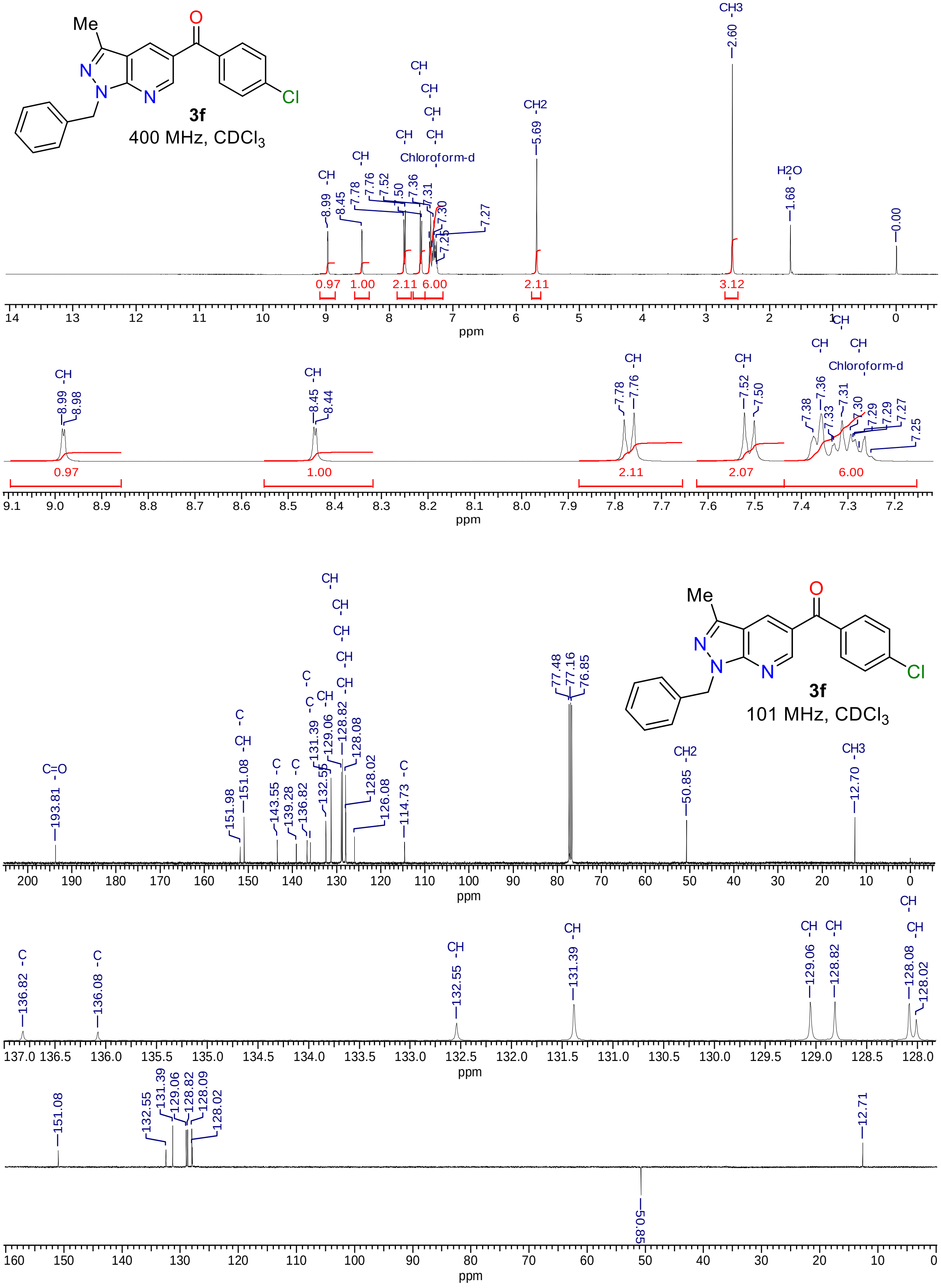

Fig. S43 ^{1}H, ^{13}C, and DEPT-135 spectra of 5-(4-chlorobenzoyl)-*N*-benzyl derivative **3f**

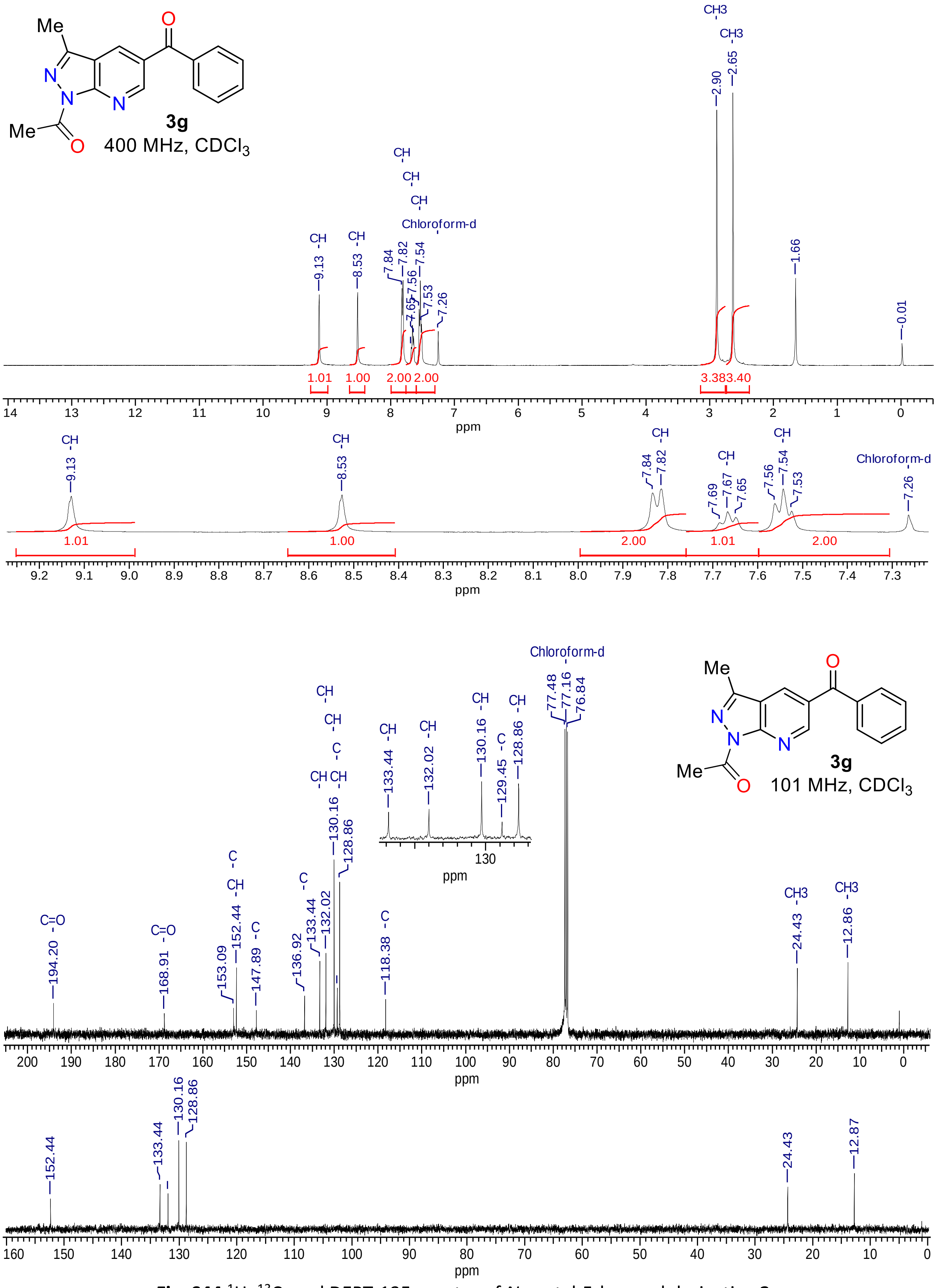

Fig. S44 ^{1}H, ^{13}C, and DEPT-135 spectra of *N*-acetyl-5-benzoyl derivative **3g**

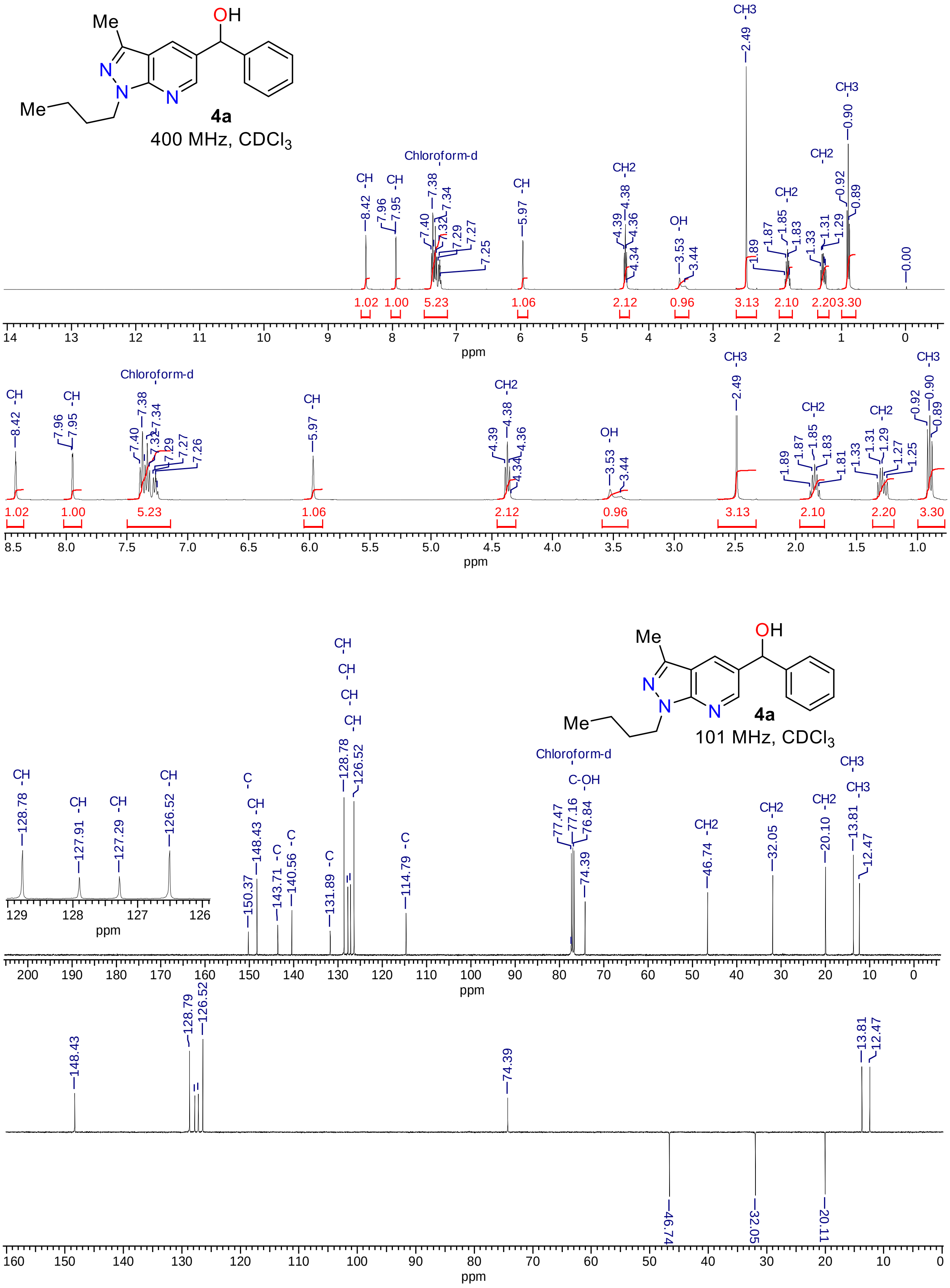

Fig. S45 ^{1}H, ^{13}C, and DEPT-135 spectra of the hetaryl phenyl methanol **4a**

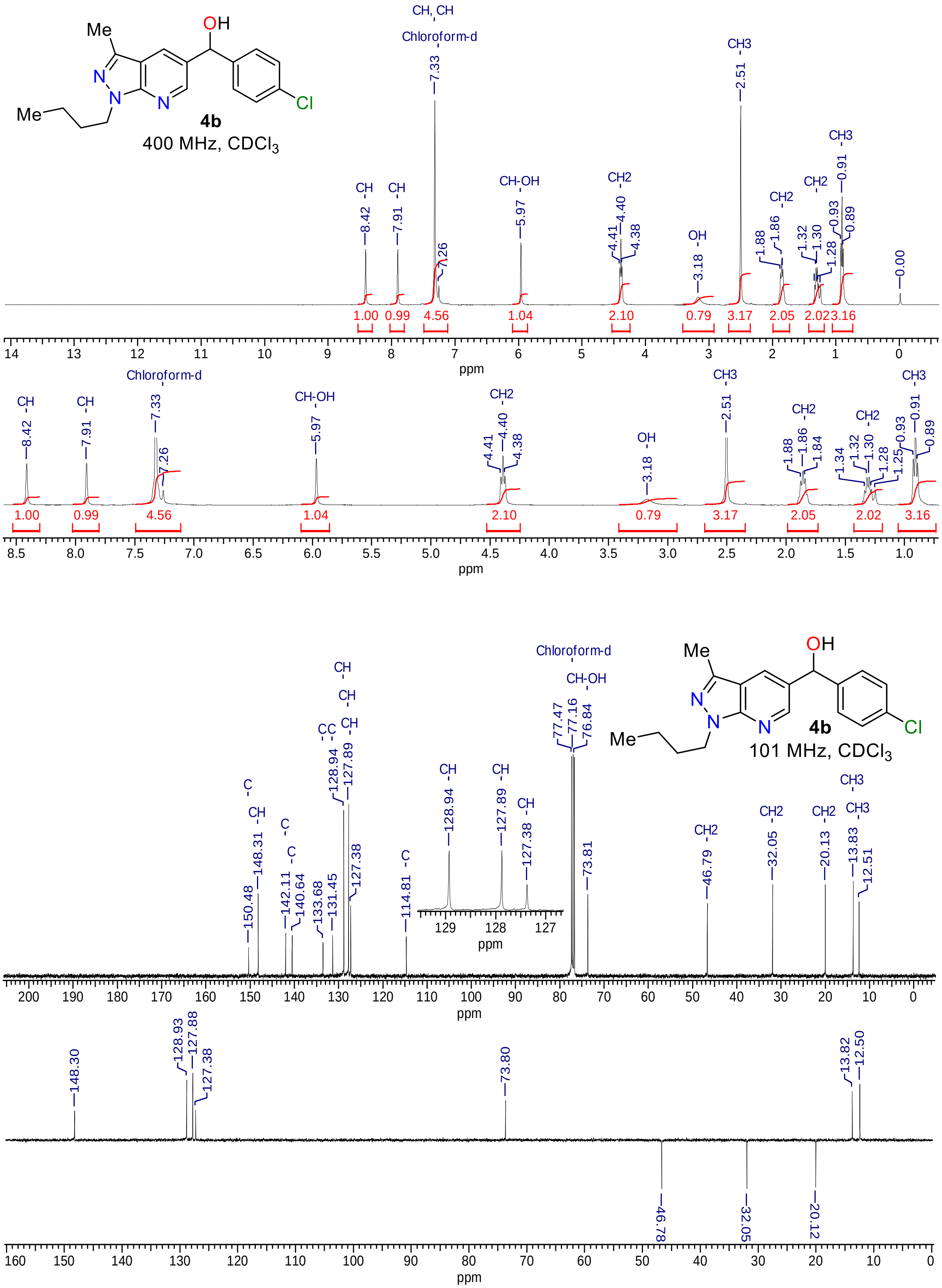

Fig. S46 ^{1}H, ^{13}C, and DEPT-135 spectra of the 4-chlorophenyl hetaryl methanol **4b**

6. Crystallographic and photophysical details

The X-ray intensity data were measured at 25(2) °C using CuKα radiation (λ = 1.54184 Å), by ω scans in an Agilent SuperNova, Dual, Cu at Zero, Atlas four-circle diffractometer equipped with a CCD plate detector. The collected frames were integrated with the CrysAlis PRO software package (CrysAlisPro 1.171.39.46e, Rigaku Oxford Diffraction, 2018). Data were corrected for the absorption effect using the CrysAlis PRO software package by the empirical absorption correction using spherical harmonics, implemented in the SCALE3 ABSPACK scaling algorithm. Crystal data, data collection, and structure refinement details are summarized in Table S1. H atoms were placed in calculated positions (C—H = 0.93 - 0.98 Å) and included as riding contributions, with isotropic displacement parameters set at 1.2–1.5 times the U_{eq} value of the parent atom. H atoms belonging to NH groups were in different density maps and were refined freely. The crystal structures were solved using an iterative algorithm[5] and subsequently completed by a different Fourier map and refined using the program SHELXL2014.[6] Mercury software generated Molecular and supramolecular graphics (Figures S47 and S48).[7]

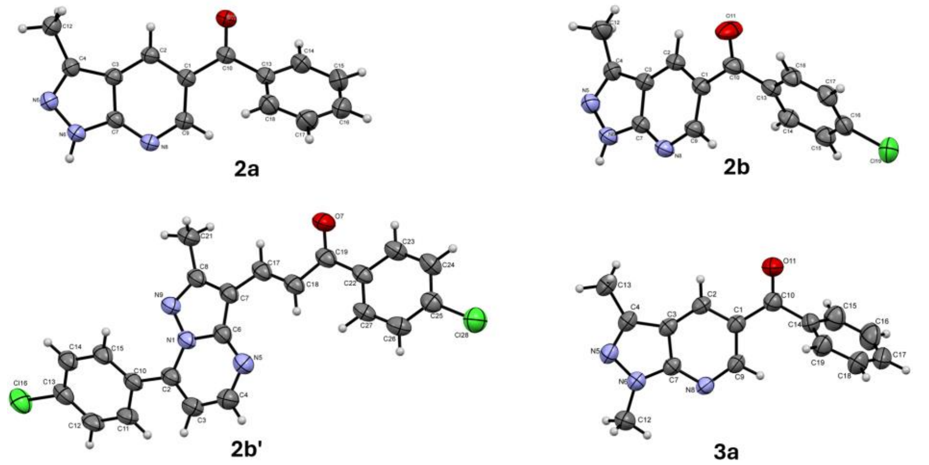

Fig. S47 Molecular structures of **2a**, **2b**, **2b'**, and **3a** obtained by X-ray diffraction data

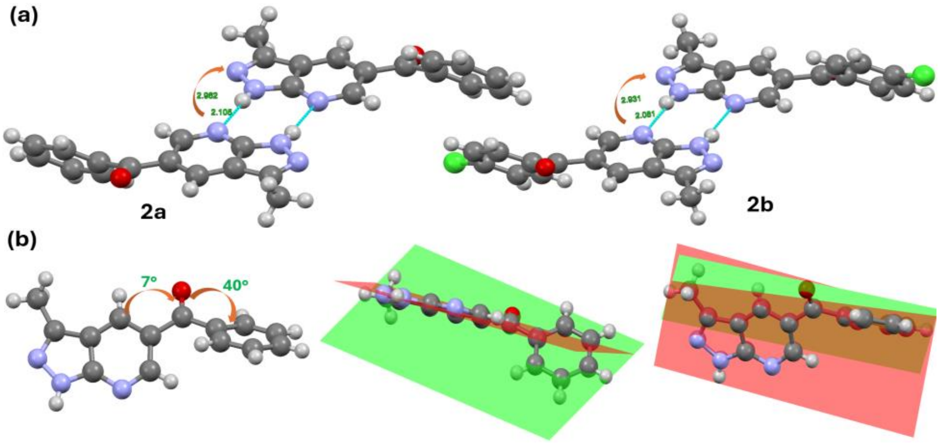

Fig. S48 (a) Hydrogen interactions (C-H···O) connect molecules in the crystal of N*H*-pyrazolo[3,4-*b*]pyridines **2a-b** and (b) dihedral angles between the planes of the heterocyclic nucleus and the aroyl group for **2a**.

The fluorescence quantum yields (φ_F) were determined using Prodan® as a reference standard and according to equation S1.

$$\phi_{f,x} = \phi_{f,st} \frac{F_x}{F_{st}} \frac{A_{st}}{A_x} \frac{n_x^2}{n_{st}^2} \qquad \text{Eq S1.}$$

F is the integral photon flux, A is the absorption factor, n is the refractive index of the solvent and ϕ_f is the quantum yield. The index x denotes the sample, and the index st denotes the standard.[8]

Lippert-Mataga correlation: The sensitivity to medium polarity is often interpreted in terms of the Lippert-Mataga equation (Eq S2).

$$\nu_{abs} - \nu_{em} = \frac{2(\mu_e - \mu_g)\Delta f}{hca^3} \quad \Delta f = \frac{(\varepsilon - 1)}{(2\varepsilon + 1)} - \frac{(n^2 - 1)}{(2n^2 + 1)} \qquad \text{Eq S2.}$$

$\Delta\mu$ are the Stokes shift derived from the difference between the wavenumbers of absorption and emission maxima, μ_g and μ_e are the ground state and excited state dipole moments, respectively. Likewise, ε is the solvent dielectric constant, η is the refractive index, *h* is Planck's constant, c is the light velocity, and *a* is Onsager cavity radius.[9]

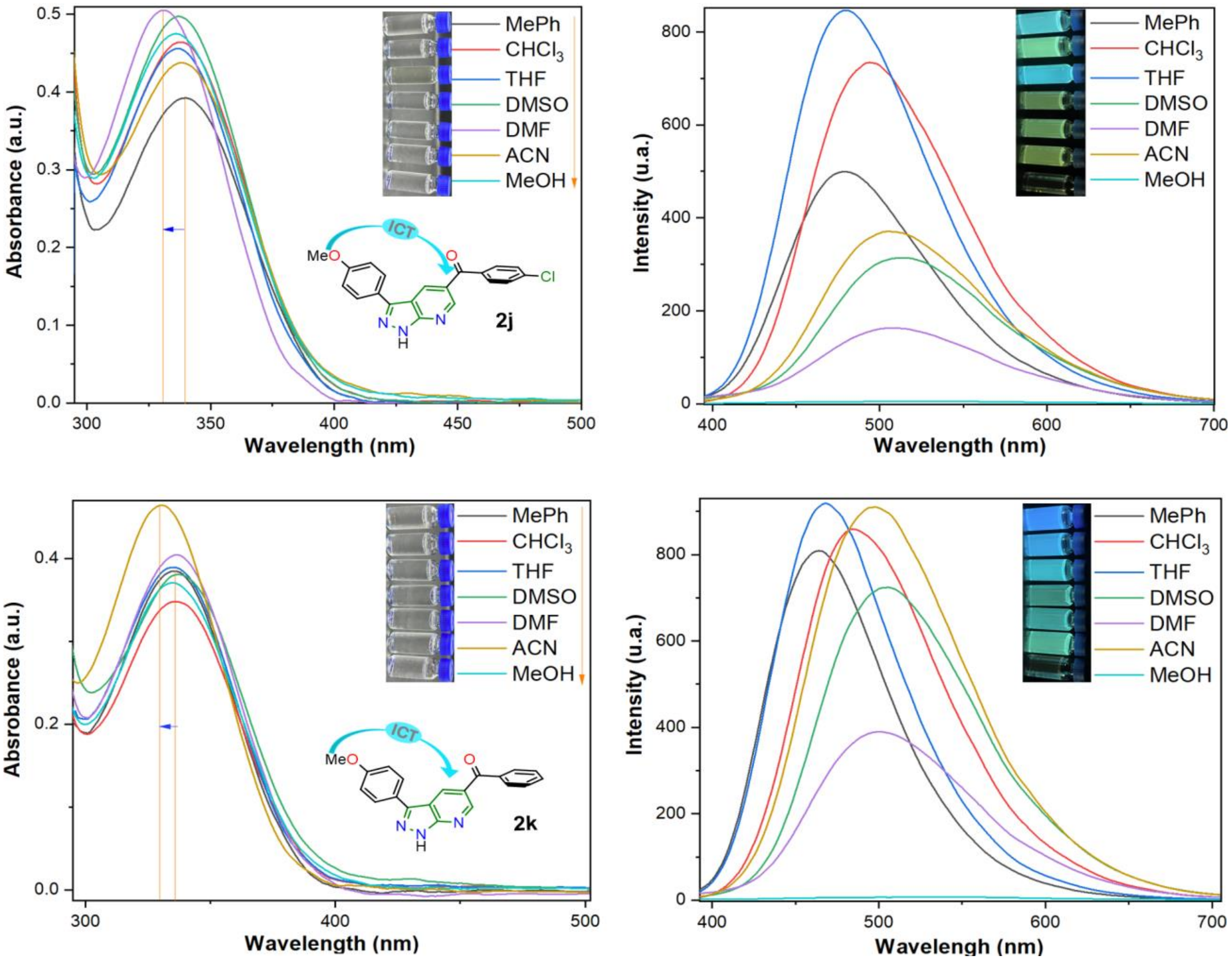

Fig. S49 UV-vis (left) and emission (right, λ_{ex} = 335 nm) spectra of **2j-2k** in different solvents (50 µM) at 20 °C.

7. Computational studies

All calculations were performed using the software Gaussian 16[10] with the B3LYP functional.[11] The basis set 6-311G +(d,p) was used for all atoms. The geometries of all critical points on the Potential Energy Surface (PES) were optimized using the gradient method available in Gaussian 16. Vibrational frequency analyses were carried out to characterize all critical points as minima or transition states. An aqueous medium was used as an implicit solvent model (dielectric constant ε = 78.39). Molecular graphics and orbital visualizations were generated with GaussView6.[12]

Compound	HOMO	LUMO
**1a**	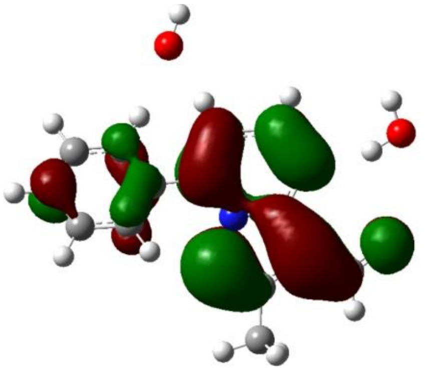	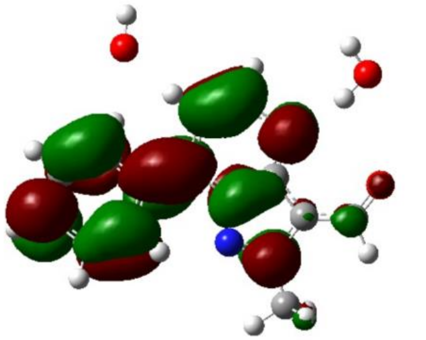
**2a**	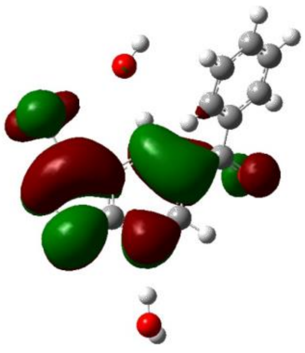	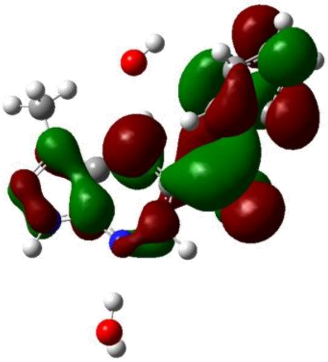
$\mathbf{I_3}$	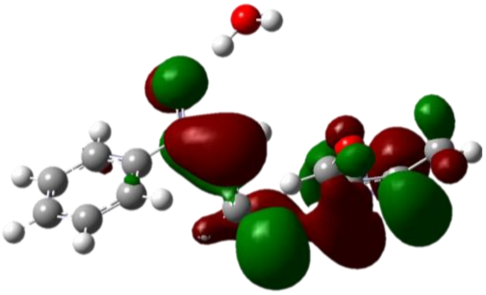	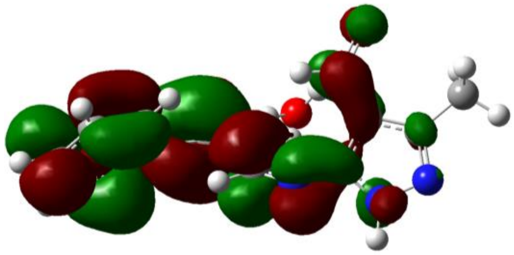

Fig. S50 Estimation of HOMO and LUMO of substrate **1a**, product **2a**, and intermediate $\mathbf{I_3}$.

Compound	DFT
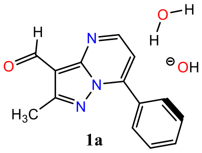 **1a**	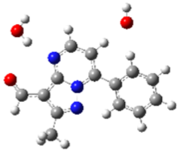
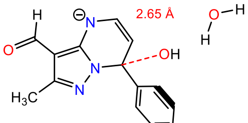 **TS_1**	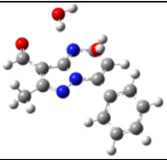
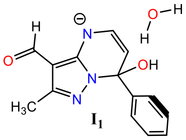 **I_1**	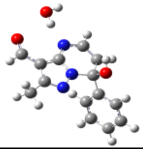
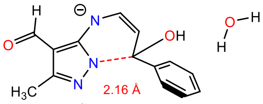 **TS_2**	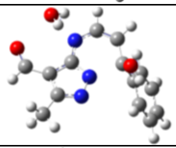
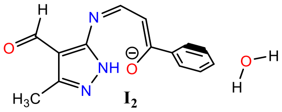 **I_2**	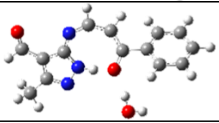
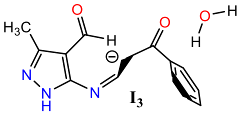 **I_3**	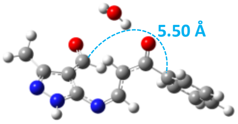
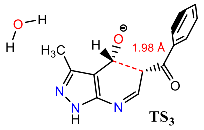 **TS_3**	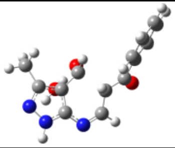
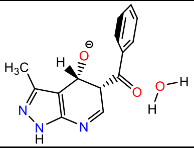	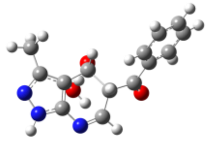
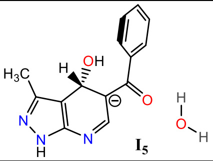 **I_5**	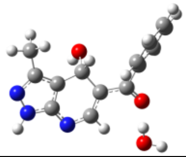
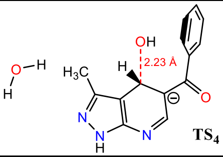 **TS_4**	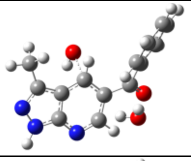
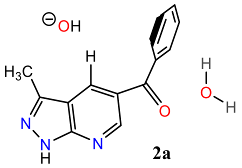 **2a**	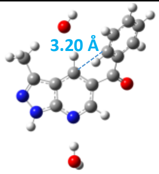

Figure S51. Structure for compounds **1a**, **2a**, intermediate **I_1** to **I_5** and transition states **TS_1** to **TS_4**

Cartesian coordinates of all the discussed critical points

Structure 1a

Charge:-1, Spin: 1, Energy: -932.224628 Hartree

C -1.60280400 0.04995100 -0.01044400
C -0.79036300 2.15748800 -0.13786600
C 0.53728300 1.71405000 -0.11328700
C 0.80719000 0.35346300 -0.05583000
C -1.56791500 -2.18430500 0.21453900
C -2.44960600 -1.07430800 0.10769500
H -0.98965000 3.22292800 -0.19693100
H 1.34259100 2.45499900 -0.11119000
N -1.85400600 1.35992700 -0.10571800
N -0.30034100 -0.45849800 0.01629500
N -0.29158400 -1.82177600 0.16058800
C 2.18167600 -0.18813700 -0.07593500
C 2.49819300 -1.45648500 -0.59580200
C 3.22691500 0.63442900 0.38750800
C 3.81975100 -1.89119900 -0.63807600
H 1.71434600 -2.09806900 -0.96668000
C 4.54236700 0.18169500 0.35582100
H 3.02300800 1.63859000 0.74678500
C 4.84579500 -1.08124500 -0.15283700
H 4.04565600 -2.86810900 -1.04994100
H 5.33438700 0.82340300 0.72501600
H 5.87266800 -1.42817600 -0.17764300
C -1.91624400 -3.62972800 0.37669900
H -2.50101700 -3.99113000 -0.47307400
H -1.00495100 -4.22344900 0.44858700
H -2.51028700 -3.79226400 1.27944900
C -3.88757200 -1.12693500 0.13745100
H -4.30247400 -2.14641700 0.24871700
O -4.65192400 -0.17008700 0.05195800
O 2.73555300 3.78974300 0.42675500
H 2.67382500 4.74482200 0.54326600
H -3.61154800 2.33390100 -0.28711400
O -4.28885400 3.02904500 -0.40649100
H -3.79123600 3.82938400 -0.60299600

Structure TS_1

Charge:-1, Spin: 1, Energy: -932.213951 Hartree

C 1.48650800 -0.06545000 -0.15973300
C 0.94386800 -2.24572500 -0.45314700
C -0.41908300 -1.97005800 -0.35693200
C -0.85266600 -0.67161000 -0.13019600
C 1.16409400 2.13100300 0.19471000
C 2.18325800 1.15473800 0.00289600
H 1.27366600 -3.26473300 -0.62855300
H -1.14460400 -2.76180800 -0.46945300
N 1.89963500 -1.32160400 -0.35873400
N 0.13582500 0.26685500 -0.07252900
N -0.05319200 1.60538200 0.15344800
C -2.28202600 -0.29704200 -0.17940800
C -2.72844100 0.79890000 -0.93226800
C -3.22685800 -1.12226500 0.44749000
C -4.09179300 1.06136800 -1.05055900
H -2.01425100 1.44057800 -1.42898700
C -4.58678700 -0.85170900 0.33257600
H -2.88261900 -1.95076300 1.05150200
C -5.02463700 0.24151700 -0.41696800
H -4.42318400 1.90826600 -1.64050000
H -5.30502500 -1.49042100 0.83393100
H -6.08434900 0.45238800 -0.50553000
C 1.32634900 3.60038800 0.42272900
H 1.83699500 4.07550900 -0.41889000
H 0.34783000 4.06567600 0.54269700
H 1.91804600 3.79669700 1.32041000
C 3.59788000 1.40264400 -0.02331100
H 3.87579700 2.46172300 0.13719500
O 4.48276800 0.56879800 -0.20497100
O -0.61290400 -1.02602800 2.48370600
H -1.09861800 -0.78879700 3.28360300
H 3.74085400 -2.07903700 -0.41861900
O 4.50710600 -2.68664800 -0.46765300
H 4.12983000 -3.56888100 -0.39002000

Structure I_1

Charge:-1, Spin: 1, Energy: -932.240727 Hartree

C 1.44468800 -0.11561300 0.09823400
C 0.83614800 -2.30363800 0.13525800
C -0.44346400 -2.04123200 0.50087400
C -0.91547400 -0.65389000 0.82793500
C 1.17483600 2.13676600 0.24385100
C 2.15133500 1.12430600 -0.04001500
H 1.12649500 -3.33737300 -0.03760300
H -1.16731700 -2.83894200 0.60945300
N 1.83455100 -1.38177600 -0.06300700
N 0.15834300 0.24858400 0.43057000
N -0.00116100 1.62232800 0.53059300
C -2.22605600 -0.30006600 0.11227500
C -2.21143600 -0.09518800 -1.27250000
C -3.44675100 -0.24598200 0.78825000
C -3.38972900 0.16641800 -1.96605000
H -1.27054200 -0.13863000 -1.80942000
C -4.63084200 0.01374400 0.09278700
H -3.47038800 -0.40498200 1.85756300
C -4.60766000 0.22167500 -1.28418500
H -3.35884800 0.32665200 -3.03814000
H -5.57060800 0.05376100 0.63275600
H -5.52651400 0.42404000 -1.82306600
C 1.36297500 3.62353100 0.24001900
H 1.67409800 3.98468200 -0.74441900
H 0.42513400 4.11357600 0.50569800
H 2.12904600 3.93152200 0.95718300
C 3.50819400 1.37184500 -0.38575500
H 3.76896200 2.44887100 -0.42169100
O 4.39303100 0.54199600 -0.64059900
O -1.17320300 -0.52324500 2.24754100
H -0.47509800 -1.01382000 2.69881300
H 3.40845600 -2.20590300 -0.51659100
O 4.14909400 -2.82812500 -0.73973100
H 3.77303000 -3.70847600 -0.64119500

Structure TS_2

Charge:-1, Spin: 1, Energy: -932.192072 Hartree

C -1.38810200 0.13674700 0.09264900
C -0.52983300 2.05739100 1.04731600
C 0.59788400 1.53703700 1.63538000
C 1.24448000 0.26116100 1.34731300
C -2.40396200 -0.82263400 -0.25900100
H -0.75753500 3.09181100 1.30589400
H 1.07025800 2.10682600 2.43085600
N -0.46926800 -1.92396900 0.21015100
N -1.47564200 1.48004800 0.26831800
C 2.29465700 0.12549600 0.27887900
C 2.70950800 1.24531500 -0.44805500
C 2.89537000 -1.11499500 0.01475500
C 3.70893300 1.13197300 -1.41418500
H 2.24925500 2.20431700 -0.24662500
C 3.89469000 -1.22631900 -0.94732600
H 2.56165500 -2.01118500 0.52689000
C 4.30639500 -0.10173000 -1.66493200
H 4.02264700 2.01067700 -1.96619300
H 4.34505200 -2.19251100 -1.14371500
H 5.08458900 -0.18962300 -2.41439000
C -1.73553200 -2.08681900 -0.14416700
N -0.26120800 -0.57298700 0.30133900
C -2.28789100 -3.45972300 -0.38808400
H -3.15045300 -3.66878200 0.25159500
H -2.61460500 -3.58733200 -1.42480000
H -1.51886500 -4.20560300 -0.17912600
C -3.78367000 -0.66364600 -0.56441300
O -4.44283600 0.38467500 -0.63713100
H -4.30641900 -1.62315800 -0.75732300
O 1.47979600 -0.46746900 2.46704900
H 2.18340300 -1.11543500 2.31857500
H -2.42702900 2.71561600 -0.60165000
O -2.83131800 3.50871000 -1.05203700
H -2.08181500 4.05989200 -1.29848100

Structure I_2

Charge:-1, Spin: 1, Energy: -932.243561 Hartree

C -2.21980600 -0.52792700 -0.06811500
C -0.49505700 -2.18545000 -0.38519100
C 0.82105300 -1.69353000 -0.17853500
C 1.44784900 -0.44027200 -0.34255600
C -3.56702700 1.19824100 0.51355100
C -3.59386500 -0.15884300 0.05967000
H -0.47145200 -3.27053400 -0.52293900
H 1.51546600 -2.50560100 0.00610200
N -2.32458100 1.63020000 0.64904100
N -1.52174300 0.56570500 0.32582200
N -1.74150400 -1.74761300 -0.42060300
C 2.91975600 -0.36134000 -0.03765900
C 3.73421100 0.47717300 -0.81212300
C 3.50406400 -1.07866200 1.01628700
C 5.09821600 0.58413800 -0.55160700
H 3.29236600 1.03421600 -1.62923000
C 4.86581400 -0.95937600 1.28796400
H 2.88895900 -1.71628800 1.64025300
C 5.66839300 -0.13156800 0.50224700
H 5.71631900 1.22572700 -1.16971700
H 5.29899800 -1.50949900 2.11581100
H 6.72860100 -0.04310300 0.71092300
O 0.89186000 0.61515100 -0.79265900
C -4.72053900 2.10142400 0.82681600
H -4.34683400 3.08275700 1.12266400
H -5.37644700 2.22920400 -0.03858300
H -5.33046200 1.70605200 1.64412400
C -4.79692600 -0.90652700 -0.16929500
H -5.71822000 -0.33042600 0.04931000
O -4.91316900 -2.06833300 -0.56437000
H -0.54192000 0.73245700 0.04604800
H 1.37743000 2.34199000 -0.74383000
O 1.49612800 3.31630700 -0.74988600
H 2.28661700 3.47511600 -0.22415900

Structure I_3

Charge:-1, Spin: 1, Energy: -932.251523 Hartree

C 2.11136700 -1.05200800 -0.79395100
C 2.83896300 -0.55566900 0.32228900
C 2.34731600 -0.48951300 1.66667200
C -0.43615200 0.36466700 -0.31460700
C -0.23866600 -0.98141700 -0.65216900
C 4.14498800 -0.24874300 -0.18841500
H -1.12649300 -1.60501500 -0.74022500
N 4.22762800 -0.52609600 -1.47687000
N 2.98967100 -1.01037500 -1.82099300
N 0.88075400 -1.61842900 -0.94855000
H 2.80574800 -1.29861400 -2.77039400
C 5.32346600 0.29778800 0.55082700
H 5.10018300 1.27975600 0.97583600
H 5.60073000 -0.35356300 1.38348900
H 6.17483200 0.39113600 -0.12544400
C -1.69197500 0.96727700 -0.08050500
O -1.83616200 2.21998900 0.04391400
C -2.94950700 0.14228100 0.03259000
C -4.07267300 0.50741000 -0.72265200
C -3.05585200 -0.93480900 0.92126900
C -5.26481900 -0.20388900 -0.61234000
H -4.00280800 1.35011200 -1.40089600
C -4.25652500 -1.63504300 1.04782500
H -2.20472900 -1.21416900 1.53133100
C -5.36111700 -1.27702700 0.27639100
H -6.12036300 0.08022100 -1.21509800
H -4.32749500 -2.45799800 1.75036900
H -6.29179700 -1.82539400 0.36948800
O 2.96298700 -0.06067600 2.64535700
H 0.41881000 1.03380500 -0.30299000
H 1.31982100 -0.87467900 1.80097900
O 0.02161600 4.18288000 -0.24893200
H 0.74922600 4.00781900 0.35624700
H -0.60245100 3.42551700 -0.12986200

Structure TS_3

Charge:-1, Spin: 1, Energy: -932.217505 Hartree

C -2.41856300 0.08646400 -1.14443800
C -1.93309100 -0.55109000 -0.00532500
C -0.65443300 -0.18757400 0.69885700
C 0.25194500 0.55278800 -0.90300800
C -0.67321200 1.44671900 -1.58888600
C -2.95516200 -1.46258600 0.36155600
H -0.30381900 2.41145700 -1.93491500
N -3.97152000 -1.42027100 -0.50767300
N -3.63353800 -0.46165500 -1.40675000
N -1.92574600 1.18767300 -1.81454400
C -2.98460300 -2.40178100 1.52718300
H -2.09271800 -3.03474800 1.54879600
H -3.01827100 -1.85664100 2.47516500
H -3.86381400 -3.04567000 1.47090600
H -4.25193500 -0.23907900 -2.17185300
C 1.51103800 1.12308800 -0.45524300
O 1.60242300 2.32757800 -0.16887800
C 2.69648200 0.22375600 -0.22858600
C 3.70605300 0.65797700 0.64382200
C 2.84600000 -1.01813100 -0.86273500
C 4.82735100 -0.12926300 0.88530600
H 3.59286600 1.61873300 1.13067800
C 3.97520700 -1.80260600 -0.63101500
H 2.09578300 -1.37398600 -1.55779300
C 4.96603400 -1.36324400 0.24623000
H 5.59345100 0.21641200 1.57033600
H 4.08090000 -2.75531200 -1.13741900
H 5.84115100 -1.97643200 0.42973000
H 0.05521000 -1.02681400 0.76162800
O -0.71557400 0.59150500 1.71852700
H 0.28642400 -0.43637000 -1.35124900
O -2.73358500 2.28707500 2.19788500
H -2.00982700 1.64131500 1.97125700
H -3.48215200 2.02055100 1.65491000

Structure I_4

Charge:-1, Spin: 1, Energy: -932.221445 Hartree

C -2.58965600 0.20857900 -0.98309800
C -1.98708700 -0.58970300 -0.02609700
C -0.58848900 -0.28367900 0.47566800
C 0.10146200 0.47404800 -0.80332600
C -0.80128400 1.48172700 -1.46020000
C -2.99451400 -1.50436400 0.36224600
H -0.35563200 2.38800400 -1.86421700
N -4.12529300 -1.29262800 -0.32785100
N -3.86485400 -0.23354500 -1.13286000
N -2.07031000 1.32639000 -1.61635100
C -2.91917800 -2.59421800 1.38592700
H -2.05431400 -3.24097700 1.21289600
H -2.81883200 -2.18288000 2.39501600
H -3.82127100 -3.20771600 1.35478000
H -4.56961200 0.11008700 -1.76755800
C 1.42598000 1.06257300 -0.40304000
O 1.52005100 2.25962200 -0.15157600
C 2.61944300 0.16781300 -0.24840300
C 3.75480400 0.68278200 0.39782700
C 2.65499000 -1.15125600 -0.72412800
C 4.89159900 -0.09831800 0.56788100
H 3.72644800 1.70092900 0.76522400
C 3.79746100 -1.93275700 -0.55925300
H 1.80231900 -1.57867300 -1.23555800
C 4.91596000 -1.41010700 0.08785000
H 5.75865600 0.31095800 1.07367600
H 3.81266500 -2.94846300 -0.93712900
H 5.80271700 -2.02014900 0.21765700
H -0.01708800 -1.22881000 0.53978600
O -0.55483300 0.41252900 1.63572700
H 0.23442800 -0.33480000 -1.53227900
O -1.93529700 2.52875300 2.18530800
H -1.40784100 1.70192400 1.90589100
H -1.59775700 3.24309500 1.63644700

Structure I_5	Structure TS4
Charge:-1, Spin: 1, Energy: -932.249777 Hartree	Charge:-1, Spin: 1, Energy: -932.229181 Hartree
C -2.77498700 0.74352100 -0.20128700	C -2.76285300 0.56290900 -0.54763300
C -2.14953700 -0.47467100 0.04841600	C -2.08117900 -0.53705500 0.00636800
C -0.69661000 -0.52229300 0.37329100	C -0.68904100 -0.42669200 0.22459400
C -0.06659200 0.80260600 -0.01101200	C -0.08608500 0.74625000 -0.30600000
C -0.88434800 1.94216700 -0.21346300	C -0.90783400 1.78116600 -0.81861500
C -3.18387400 -1.44272100 -0.00787800	C -3.09095100 -1.52586600 0.22470200
H -0.37943200 2.89447400 -0.34700300	H -0.42587300 2.68845800 -1.16863700
N -4.36116500 -0.86822500 -0.27075800	N -4.27465500 -1.06887300 -0.15916700
N -4.09453000 0.46741900 -0.37710600	N -4.06895500 0.20328500 -0.61879700
N -2.20743700 1.98299100 -0.31116300	N -2.22770200 1.73145000 -0.95896600
C -3.08542800 -2.92355200 0.19125200	C -2.93911000 -2.90156500 0.78833000
H -2.32977900 -3.36260400 -0.46691700	H -2.21117300 -3.48353800 0.21571700
H -2.79971100 -3.16846200 1.21882200	H -2.58540500 -2.86715300 1.82267400
H -4.04462900 -3.39902600 -0.02098500	H -3.89563300 -3.42573200 0.76643900
H -4.83179600 1.11453600 -0.60932900	H -4.84047200 0.73890500 -0.98555900
C 1.35195700 0.96977700 -0.06706100	C 1.36051500 0.97334600 -0.26257800
O 1.94724400 2.08163600 -0.08916400	O 1.84795500 2.11565200 -0.24999400
C 2.25538300 -0.24088600 -0.13362800	C 2.30545000 -0.19122700 -0.25058200
C 3.29451600 -0.38046100 0.79440100	C 3.47878100 -0.10139700 0.51152600
C 2.14123900 -1.17865900 -1.16657600	C 2.08833100 -1.33111600 -1.03632300
C 4.18399000 -1.44965200 0.70860200	C 4.40343800 -1.14116800 0.50862400
H 3.39996200 0.35089600 1.58784200	H 3.65239500 0.78470000 1.11015700
C 3.04446200 -2.23720300 -1.26686700	C 3.02697400 -2.36132000 -1.05747900
H 1.35403900 -1.07130600 -1.90496600	H 1.19954500 -1.40225900 -1.65173700
C 4.06320100 -2.38040700 -0.32497000	C 4.18073100 -2.27265300 -0.27903300
H 4.97452500 -1.55400800 1.44363200	H 5.29895200 -1.06949600 1.11534600
H 2.95223900 -2.94810000 -2.08057500	H 2.85730500 -3.23112200 -1.68170800
H 4.76047700 -3.20747200 -0.39783600	H 4.90528400 -3.07904800 -0.28837200
H -0.20222400 -1.37538000 -0.08975000	H -0.09817100 -1.28670700 0.48603200
O -0.48597100 -0.81546100 1.81147900	O -0.51250000 -0.18702300 2.43441500
H -0.88396500 -0.08671200 2.30438100	H -1.05989000 0.60481200 2.49449100
H 1.66177500 3.81131400 -0.01799200	H 1.30202800 3.76702200 0.30320200
O 1.50180800 4.78055400 0.02190600	O 1.14096300 4.68654500 0.59341100
H 2.34556600 5.18731600 -0.19922700	H 0.36594800 4.63705300 1.16239600

Structure 2b	
Charge:-1, Spin: 1, Energy: -932.240893 Hartree	C -2.48550100 -0.79241200 0.02703500
C 2.64237200 -0.07772900 -0.11532400	C -3.68326700 -0.97686400 0.73407400
C 1.64807200 0.91520400 0.05230000	C -2.49872000 -0.05054500 -1.16164100
C 0.32316100 0.51240100 0.24168900	C -4.86668700 -0.41263400 0.27200400
C 0.09016600 -0.86740200 0.25437300	H -3.67042800 -1.55771800 1.64832800
C 1.17452400 -1.76558700 0.08372300	C -3.69081000 0.49322800 -1.63628400
C 2.35940600 2.16158300 -0.00694500	H -1.58606000 0.08381900 -1.72855700
H 0.97554000 -2.83157200 0.10053200	C -4.87292100 0.32033500 -0.91761900
N 3.64869700 1.93630000 -0.18979100	H -5.78454000 -0.54490300 0.83317800
N 3.81592200 0.57870400 -0.25967500	H -3.69534700 1.05264200 -2.56451300
N 2.43812700 -1.40461000 -0.11097000	H -5.79742000 0.75335700 -1.28243900
C 1.79389200 3.54192900 0.08341700	H -0.45690700 1.25895800 0.44003400
H 1.60895900 3.94493200 -0.91837400	O -1.30026200 2.97369500 1.17908600
H 0.83805700 3.52773700 0.61539200	H -2.18618900 3.07976100 1.54545300
H 2.49326500 4.21266800 0.58688300	H 3.78197800 -2.71975700 -0.35726400
H 4.73954700 0.19090600 -0.37780800	O 4.46896100 -3.40178900 -0.52249500
C -1.24837600 -1.46630800 0.52624300	H 4.61336700 -3.82315900 0.33114800
O -1.33181300 -2.52335900 1.14700800	

8. References

1 J. C. Castillo, H. A. Rosero and J. Portilla, *RSC Adv*, 2017, **7**, 28483–28488.

2 J. C. Castillo, A. Tigreros and J. Portilla, *Journal of Organic Chemistry*, 2018, **83**, 10887–10897.

3 A. Tigreros, S. L. Aranzazu, N. F. Bravo, J. Zapata-Rivera and J. Portilla, *RSC Adv*, 2020, **10**, 39542–39552.

4 S. Aranzazu, A. Tigreros, A. Arias-g, J. Zapata-rivera and J. Portilla, *J Org Chem*, 2022, **87**, 9839–9850.

5 L. Palatinus and G. Chapuis, *J. Appl. Crystallogr.*, 2007, **40**, 786–790.

6 G. M. Sheldrick, *Acta Crystallogr., Sect. C: Struct. Chem.*, 2015, **C71**, 3–8.

7 C. F. Macrae, I. J. Bruno, J. A. Chisholm, P. R. Edgington, P. McCabe, E. Pidcock, L. Rodriguez-Monge, R. Taylor, J. van de Streek and P. A. Wood, *J. Appl. Crystallogr.*, 2008, **41**, 466–470.

8 C. Würth, M. Grabolle, J. Pauli, M. Spieles and U. Resch-Genger, *Nat. Protoc.*, 2013, **8**, 1535–1550.

9 T. N. Moshkina, E. V Nosova, A. E. Kopotilova, M. I. Savchuk, I. L. Nikonov, D. S. Kopchuk, P. A. Slepukhin, G. A. Kim, G. N. Lipunova and V. N. Charushin, *J. Photochem. Photobiol., A*, 2022, **429**, 113917.

10 M. J. Frisch, G. W. Trucks, H. B. Schlegel, G. E. Scuseria, M. A. Robb, J. R. Cheeseman, G. Scalmani, V. Barone, G. A. Petersson, H. Nakatsuji, X. Li, M. Caricato, A. V Marenich, J. Bloino, B. G. Janesko, R. Gomperts, B. Mennucci, H. P. Hratchian, J. V Ortiz, A. F. Izmaylov, J. L. Sonnenberg, D. Williams-Young, F. Ding, F. Lipparini, F. Egidi, J. Goings, B. Peng, A. Petrone, T. Henderson, D. Ranasinghe, V. G. Zakrzewski, J. Gao, N. Rega, G. Zheng, W. Liang, M. Hada, M. Ehara, K. Toyota, R. Fukuda, J. Hasegawa, M. Ishida, T. Nakajima, Y. Honda, O. Kitao, H. Nakai, T. Vreven, K. Throssell, J. A. Montgomery Jr., J. E. Peralta, F. Ogliaro, M. J. Bearpark, J. J. Heyd, E. N. Brothers, K. N. Kudin, V. N. Staroverov, T. A. Keith, R. Kobayashi, J. Normand, K. Raghavachari, A. P. Rendell, J. C. Burant, S. S. Iyengar, J. Tomasi, M. Cossi, J. M. Millam, M. Klene, C. Adamo, R. Cammi, J. W. Ochterski, R. L. Martin, K. Morokuma, O. Farkas, J. B. Foresman and D. J. Fox, .

11 A. D. Becke, *J. Chem. Phys.*, 1993, **98**, 5648–5652.

12 R. Dennington, T. A. Keith and J. M. Millam, *GaussView*, 2019.
